# *Kazachstania pintolopesii* accelerates splenic senescence in aged mice via immune cell reprogramming, immunosenescence and amelioration by Astragaloside IV

**DOI:** 10.3389/fimmu.2026.1917140

**Published:** 2026-09-18

**Authors:** Yanqing Zhang, Yinrui Guo, Lei Li, Xiao Zhang, Haiting Zhang, Diling Chen

**Affiliations:** 1 Department of Traditional Chinese Medicine, The Affiliated Guangdong Second Provincial General Hospital of Jinan University, Guangzhou, Guangdong, China; 2 Zhuhai Hospital of Integrated Traditional Chinese and Western Medicine, Zhuhai, Guangdong, China; 3 College of Information Engineering, Tarim University, Alaer, China; 4 School of Medicine, Tarim University, Alaer, China

**Keywords:** astragaloside, ferro-aging, immune-senescence, *Kazachstania pintolopesii*, splenic senescence

## Abstract

**Introduction:**

Splenic senescence represents a hallmark of aging-related immune dysfunction, and the fungus *Kazachstania pintolopesii* is capable of aggravating senescence-associated phenotypes in aged mice. As a natural bioactive ingredient, Astragaloside IV (AGS) possesses anti-aging and immunomodulatory capacities, whereas its protective mechanisms against *K. pintolopesii*-triggered splenic senescence remain poorly defined. This study aimed to clarify the molecular mechanisms underlying *K. pintolopesii*-induced splenic senescence and validate the protective efficacy of AGS.

**Methods:**

This study integrated *in vivo* interventions, multi-omics sequencing, comprehensive histopathological examination, multidimensional bioinformatics analysis, and cross-species conservation validation to systematically explore the pathogenic role of *K. pintolopesii* and the protective action of AGS. scRNA-seq and bulk RNA-seq were applied to profile splenic transcriptional alterations. Histopathological staining including H&E, Masson, Prussian blue, and IHC staining was performed to visualize splenic structural impairment, fibrosis, iron deposition, and target protein expression. Bioinformatics analyses containing UMAP dimensionality reduction, pseudotime trajectory analysis, DEG screening, and GO/KEGG functional enrichment were conducted to dissect key molecular pathways.

**Results:**

The results showed that *K. pintolopesii* induced characteristic splenic structural changes in aged mice, including white pulp lymphocyte rarefaction, mild interstitial fibrosis, and prominent iron overload, accompanied by immune subset remodeling. Mechanistically, K. pintolopesii promoted the differentiation of naive T cells into effector/memory phenotypes, drove B cell polarization toward a pro-senescent marginal zone signature, and elicited myeloid cell reprogramming with activated ferroptosis. These pathological events originated from the synergistic crosstalk between ferro-aging (iron overload and ACSL4-mediated lipid peroxidation) and chronic inflammation. Moreover, AGS intervention significantly attenuated KP-associated pathological changes by restoring splenic immune function, modulating iron metabolism and redox homeostasis, suppressing ferroptosis/ferro-aging signaling, and disrupting the senescence-inflammation positive feedback loop.

**Discussion:**

In conclusion, *K. pintolopesii* exacerbates splenic senescence in aged mice via the synergistic interaction between immune senescence and ferro-aging. AGS exerts protective effects on the spleen by targeting the iron-immune axis. This study demonstrates that AGS is a promising therapeutic candidate for the intervention of aging-associated splenic immune dysfunction, providing novel insights into the mechanism of fungal-induced splenic senescence and potential anti-aging therapeutic strategies.

## 1 Introduction

The spleen is a central immune organ and a core regulatory node for systemic iron metabolism (1–3), acting as an indispensable bridge connecting innate and adaptive immunity (4–6). Its structural integrity and functional homeostasis are fundamental to maintaining systemic immune balance and iron metabolic stability (7). During aging, the spleen undergoes characteristic senescent remodeling, manifested as immune cell subset dysregulation, impaired immune effector function, iron metabolic perturbation, and the onset of chronic low-grade inflammation, a pathological state defined as splenic senescence (7). Accumulating evidence has confirmed that iron accumulation and the pro-oxidative microenvironment in the aged spleen directly compromise T cell function; even naive T cells from young hosts lose immunocompetence upon transient exposure to the senescent splenic microenvironment (8–11). This pathological cascade not only impairs the body’s immune defense against pathogens and neoplasms but also exacerbates systemic aging via dysregulation of the immune-iron metabolism axis, making it a pivotal driver of immune senescence and the high incidence of metabolic disorders in the elderly (12, 13). Thus, elucidating the regulatory mechanisms of splenic senescence and identifying its key modulators hold profound theoretical value and clinical implications for delaying organismal aging and promoting healthy longevity.

The gut microbiota exerts remote regulatory effects on splenic structure and function through immune modulation and metabolite translocation via the gut-spleen axis, serving as a critical environmental determinant of immune senescence (14, 15). Among intestinal microbial constituents, fungi, an integral part of the gut microecosystem, have recently emerged as a burgeoning research focus due to their intricate interactions with host aging (16–18). Mounting studies have revealed that gut fungi modulate the phenotypic and functional profiles of splenic immune cells via innate immune recognition cascades and bioactive metabolite secretion (19, 20). For example, *Candida albicans* activates the splenic TLR2/Dectin-1/2 signaling pathway to drive myeloid cell subset reprogramming (21, 22), while intestinal fungal dysbiosis induces peripheral immune dysfunction via mononuclear phagocytes, indirectly disrupting splenic immune homeostasis (19, 23, 24). While some commensal fungi exert immunostimulatory effects on the spleen through metabolic products (e.g., *Flammulina velutipes* polysaccharides enhance splenic immunity by activating the NF-κB/MAPK cascade) (25), the regulatory effects of pathogenic or opportunistic fungi on the aged spleen remain largely uncharacterized. Furthermore, early-life exposure to the native mycobiota shapes the generation of splenic memory B cells, underscoring an underappreciated role of gut fungi in regulating splenic immune senescence that demands systematic investigation (26, 27).

Notably, our research team has conducted extensive preliminary studies on *Kazachstania pintolopesii* (*K. pintolopesii*), a characteristic fungus isolated from spontaneously occurring ankylosing spondylitis (AS) cynomolgus monkeys (28). We have confirmed that *K. pintolopesii* is a core signature fungus in the intestines of AS monkeys, with a higher enrichment degree than bacterial groups, and its fecal metabolites can activate the IL-17/IL-23 pathway and adipogenesis-related pathways in umbilical cord blood stem cells (28). In animal models, *K. pintolopesii* lysate (LKP) can induce severe inflammatory responses and autoimmune pathology in BALB/c ZAP70^W163C^ mutant mice: direct injection into paravertebral muscle tissues leads to skin ulceration, joint swelling, spinal deformation, and structural disorganization of articular regions (talocrural, metatarsophalangeal, interphalangeal joints), accompanied by significant upregulation of pro-inflammatory cytokines (IL-1β, IL-6, NF-κB) in spinal and joint tissues (29). Single-nucleus RNA sequencing of joint tissues from treated mice identified 10 major cell types, with altered interaction networks and enriched pathways including HIF-1 signaling, rheumatoid arthritis, IL-17 signaling, and ECM-receptor interaction in T cells. Primary spleen T cells from ZAP70^W163C^ mutant mice treated with LKP showed significant upregulation of inflammatory markers (Cxcl1, IL-22, IL-1β, Saa3, IL-6, TNF, IL-17F) and enrichment of TNF signaling, IL-17 signaling, and inflammatory bowel disease pathways (29).

At the cellular level, *K. pintolopesii* lysate and secreted proteins directly activate the macrophage pyroptosis pathway, induce high expression of pro-inflammatory factors such as iNOS and TNF-α, and interact with RAW264.7 cells and primary bone marrow macrophages to enrich multiple AS-related inflammatory pathways (28, 29). In-depth subpopulation analysis of mononuclear phagocytes revealed 11 distinct subsets, with Mac_Adam8 and Mac_mt-Co1 positively associated with arthritis and spondylitis; these subsets showed activated ferroptosis, TNF signaling, IL-17 signaling, and rheumatoid arthritis pathways, along with upregulated cholesterol metabolism. Bone marrow-derived macrophages from ZAP70^W163C^ mutant mice treated with *K. pintolopesii* lysate exhibited 2,622 differentially expressed mRNAs, including upregulated pro-inflammatory cytokines (IL-1β, IL-6, IL-18, TNF-α) and PANoptosome components (28, 29). Additionally, *K. pintolopesii* induces osteoblast fibrosis, abnormal proliferation of fibroblast-like synoviocytes (FLS), and significant changes in endothelial cell subsets, VECs_Mcam and VECs_Col15a1 subpopulations were enriched in focal adhesion, ECM-receptor interaction, and IL-17 signaling pathways, with downregulated mitochondrial genes (*mt-Co1*, *mt-Atp6*) indicating metabolic dysfunction. In normal C57BL/6 mice, *K. pintolopesii* induces AS-like symptoms (intestinal inflammation, spinal tissue inflammatory infiltration, IL-23/IL-17 pathway activation) and regulates spinal transcriptome changes via the gut-spinal cord axis; its fungal proteins also cause weight gain, hepatic lipid deposition, and abnormal spinal lipid metabolism in KunMing mice, with significant remodeling of intestinal flora structure (28, 29). These preliminary findings have clearly confirmed the close association between *K. pintolopesii* and inflammation, metabolic disorders, autoimmune pathology, and AS-like pathological changes, laying a solid experimental foundation for further exploring its systemic regulatory effects on the spleen.

Preliminary studies have demonstrated *K. pintolopesii’s* ability to modulate local intestinal immune responses and perturb metabolic homeostasis (30–33), yet its systemic effects on the spleen, a distal immune organ, and particularly its regulatory mechanisms underlying the structural and functional remodeling of the aged spleen (including immune cell subset dynamics and iron metabolic homeostasis) remain entirely unreported. Notably, *K. pintolopesii*-induced ferroptosis, IL-17/TNF pathway activation, and immune cell subset reprogramming in previous studies are closely linked to the core features of splenic senescence, while its role in iron metabolism disturbance (a key trigger of ferro-aging) has not been addressed in the context of splenic aging. Recently, the novel ferro-aging theory proposed in Cell Metabolism has established a causal link between iron overload, ACSL4-mediated lipid peroxidation, and organ senescence, providing a pioneering theoretical framework for deciphering metabolic dysregulation-driven splenic senescence (34). Ferro-aging is defined herein as an iron overload-driven aging framework integrating ferroptosis, lipid peroxidation, iron metabolism disorder, and cellular senescence. Operationally, ferro-aging is characterized by: (i) tissue iron accumulation (Prussian blue positivity); (ii) ACSL4-mediated lipid peroxidation; (iii) enrichment of ferroptosis-related pathways (KEGG mmu04216); and (iv) concurrent cellular senescence markers (p16/p21 upregulation). Ferroptosis represents the cell-autonomous regulated cell death pathway executing iron-dependent cell damage, while ferro-aging encompasses the broader systemic aging cascade initiated by iron dysregulation, of which ferroptosis is one downstream consequence. Whether *K. pintolopesii* accelerates splenic senescence by perturbing ferro-aging-related signaling cascades, and whether these effects are consistent with its previously observed pro-inflammatory and pro-fibrotic mechanisms, is thus an urgent unresolved question in the field.

Astragaloside (AGS), the principal bioactive constituent of the traditional Chinese medicinal herb *Astragalus membranaceus*, exhibits well-documented immunomodulatory, antioxidant, anti-senescence, and iron metabolism-regulating properties (35–40), with promising potential in delaying organ senescence and ameliorating immune deficiency. Our previous work has preliminary verified that natural products can intervene in *K. pintolopesii*-induced metabolic abnormalities and inflammatory responses, yet AGS’s therapeutic efficacy in reversing fungus-induced splenic senescence, as well as its specific molecular targets and regulatory pathways (especially in targeting ferroptosis, IL-17 signaling, and iron metabolism), remains elusive.

Against this backdrop, the present study used naturally aged mice as experimental models and integrated state-of-the-art techniques including single-cell RNA sequencing (scRNA-seq), histopathological staining, and multi-omics functional enrichment analysis to systematically elucidate the core molecular mechanisms by which *K. pintolopesii* aggravates senescence-associated phenotypes in aged mice. Concurrently, we investigated the protective effects and underlying molecular targets of AGS against *K. pintolopesii*-induced splenic senescence, and performed cross-species characteristic analysis of fungal exposure-mediated splenic senescence between humans and mice. This study aims to fill the critical knowledge gap regarding the systemic effects of *K. pintolopesii* on splenic aging, refine the gut-spleen axis-mediated microbiota-host aging interaction network, and provide robust experimental evidence and molecular insights for developing targeted anti-splenic senescence strategies targeting the gut mycobiota, iron metabolism, and immune cell senescence. It also lays a solid molecular foundation for the translational application of AGS in intervening in aging-related splenic dysfunction.

## 2 Materials and methods

### 2.1 Experimental animals

Specific pathogen-free (SPF) male C57BL/6 mice (20 months old ± 1 week, naturally aged) weighing 30–35 g were purchased from the Experimental Animal Center of Duangdong Province (License No.: SCXK-[Year]-0001). Mice were housed in a standardized SPF animal facility with a 12 h light/dark cycle (light from 8:00 to 20:00), constant temperature (22 ± 2°C), and relative humidity (50 ± 5%), with free access to sterile drinking water and standard laboratory chow.

A total of 45 mice were randomly divided into three groups (n=15 per group) using a random number table method, and all experimental operations (gavage, sample collection, staining) and result analyses were conducted in a double-blind manner to avoid subjective bias. All animal experiments were approved by the Institutional Animal Care and Use Committee (IACUC) of Bojin Biotechnology (Approval No.: BG-SMP-001V1-001) and strictly complied with the National Institutes of Health (NIH) Guide for the Care and Use of Laboratory Animals, as well as the 3R principles (Replacement, Reduction, Refinement). Refinement measures included optimizing gavage techniques to minimize animal stress and using non-invasive methods for health monitoring. During the 4-week intervention period, the general condition of mice (mental state, activity level), fecal traits (color, shape), and mortality were recorded daily; no mice died during the experiment, and no additional samples were supplemented. The 15 mice per group were allocated as follows: 3 mice for histopathological staining (H&E, Masson, Prussian blue, IHC, IF), 3 mice for snRNA-seq/scRNA-seq, 3 mice for bulk RNA-seq (KP vs. KP+AGS comparison), 3 mice for TCR repertoire analysis, and 3 mouse for metabolomic profiling. This allocation was designed to ensure adequate statistical power for each assay while minimizing animal use in accordance with the 3R principles.

### 2.2 Microbial strain and reagents

*Kazachstania pintolopesii* (identified and isolated by the laboratory in the early stage through its own cultivation and examination (28, 29)) and cultured in YPD medium (1% yeast extract, 2% peptone, 2% glucose) at 37°C with constant shaking (180 rpm) to the logarithmic growth phase. The bacterial suspension was centrifuged (5000 rpm, 10 min), washed three times with sterile phosphate-buffered saline (PBS), and adjusted to a final concentration of 1×10^9^ CFU/mL for animal intervention. Astragaloside IV (AGS, purity ≥98%) was purchased from MUST (Chengdu) Biotechnology CO., LTD (A0070) and dissolved in 0.5% carboxymethyl cellulose sodium (CMC-Na) to prepare a stock solution of 10 mg/mL. Hematoxylin-eosin (H&E) staining kit, Masson trichrome staining kit, and Prussian blue iron staining kit were purchased from Beyotime Biotechnology (China, Cat. Nos.: C0105, C0130, C0150). RNA extraction kits (TRIzol Reagent) were purchased from Thermo Fisher Scientific (USA, Cat. No.: 15596026). To verify successful gut delivery and exposure, fecal samples were collected weekly during the intervention period and cultured on Sabouraud dextrose agar (SDA) plates at 37 C for 48–72 h. Recovered colonies were confirmed as K. pintolopesii by ITS region sequencing. However, comprehensive quantitative colonization assessment (e.g., serial fecal ITS qPCR, CFU enumeration at multiple time points, and engraftment duration analysis) was not performed, which is acknowledged as a study limitation.

Astragaloside IV (AGS, purity ≥98%) was purchased from MUST (Chengdu) Biotechnology CO., LTD (Cat. No.: A0070), and its purity was verified by high-performance liquid chromatography (HPLC) with a detection wavelength of 203 nm. AGS was dissolved in 0.5% carboxymethyl cellulose sodium (CMC-Na) to prepare a 10 mg/mL stock solution, which was stored at 4°C and used within 1 week. The dosage of AGS (10 mg/kg body weight) was determined based on previous literature reports (AGS intervention dosage range for aging-related diseases: 5–20 mg/kg) and our pre-experiment results (dose gradient: 5 mg/kg, 10 mg/kg, 20 mg/kg), where 10 mg/kg showed optimal protective effects on splenic structure without obvious side effects.

Hematoxylin-eosin (H&E) staining kit (Cat. No. C0105), Masson trichrome staining kit (Cat. No. C0130), and Prussian blue iron staining kit (Cat. No. C0150) were purchased from Beyotime Biotechnology (Shanghai, China). RNA extraction kits (TRIzol Reagent, Cat. No.: 15596026) were obtained from Thermo Fisher Scientific (Waltham, USA). Magnetic-activated cell sorting (MACS) CD3^+^ T cell isolation kits (Cat. No. 130-096-433) were purchased from Miltenyi Biotec (Bergisch Gladbach, Germany). Primary antibodies against p16^INK4a^ (ab189034), p21^Waf1/Cip1^ (ab188224), GPX4 (ab125066), TFR2 (ab220115), and rabbit IgG isotype control (ab172730) were purchased from Abcam (UK). Horseradish peroxidase (HRP)-conjugated goat anti-rabbit secondary antibody (ab6721) and DAB substrate kit (ZLI-9018) were obtained from Abcam and ZSGB-BIO (China), respectively. All other reagents were of analytical grade or higher.

### 2.3 Isolation and stimulation of splenic T cells

Spleens were harvested under aseptic conditions and mechanically dissociated into single-cell suspensions using a sterile glass homogenizer. Red blood cells were lysed with RBC lysis buffer at room temperature for 5 min, and the reaction was terminated with an equal volume of RPMI 1640 medium. The cell suspension was filtered through a 70-μm cell strainer, and CD3^+^ T cells were purified via MACS according to the manufacturer’s instructions. The purity of sorted CD3^+^ T cells was verified by flow cytometry (BD FACSCanto II, USA) with an anti-mouse CD3-PE antibody, and only cell populations with CD3^+^ purity ≥90% were used for subsequent experiments.

Purified splenic T cells were cultured in RPMI 1640 medium supplemented with 10% fetal bovine serum (FBS, same batch), 100 U/mL penicillin, and 100 μg/mL streptomycin at 37°C with 5% CO_2_. *K. pintolopesii* cell lysate was prepared by ultrasonic disruption (300W, 3s working/5 s interval, total 15 min, 4°C) followed by centrifugation (12000rpm, 20min, 4°C) to collect the supernatant. The protein concentration of the lysate was determined using a BCA protein assay kit and adjusted to 100 μg/mL. T cells were seeded in 6-well plates at a density of 2×10^6^ cells/well and treated with 100 μg/mL *K. pintolopesii* cell lysate for 24 h; untreated T cells served as the control group. After incubation, cells were collected by centrifugation (800 rpm, 5 min, 4°C) for bulk RNA-sequencing (RNA-seq) analysis.

### 2.4 Isolation and culture of bone marrow-derived macrophages

Bone marrow cells were flushed from the femurs and tibias of mice with RPMI 1640 medium using a sterile syringe, filtered through a 70-μm cell strainer, and centrifuged at 800 rpm for 5 min at 4°C to collect the cell pellet. L929-conditioned medium was prepared by culturing L929 cells in DMEM medium containing 10% FBS for 72 h, filtering the supernatant through a 0.22-μm filter, and storing at -20°C. Bone marrow cells were resuspended in L929-conditioned medium supplemented with 20 ng/mL macrophage colony-stimulating factor (M-CSF) and seeded in 10-cm culture dishes at a density of 5×10^6^ cells/dish. The medium was changed every 3 days, and BMDM differentiation was induced for 7 days. Mature BMDMs were identified by flow cytometry using anti-mouse CD11b-APC and F4/80-PE antibodies, with a differentiation rate ≥95%.

Mature BMDMs were seeded in 6-well plates at a density of 1×10^6^ cells/well and allowed to adhere for 12 h. Cells were treated with 100 μg/mL *K. pintolopesii* lysate for 24 h at 37°C with 5% CO_2_; parallel untreated BMDMs were used as controls. After stimulation, cells were washed twice with PBS, and total RNA was extracted for transcriptomic profiling.

### 2.5 Animal grouping and intervention

A total of 45 aged C57BL/6 mice were randomly divided into three groups (n=15 per group), Control group: Mice were intragastrically administered 200 μL sterile PBS once daily for 4 weeks; *K. pintolopesii*-treated group: Mice were intragastrically administered 200 μL *K. pintolopesii* suspension (1×10^9^ CFU/mL) twice for week and last 4 weeks; *K. pintolopesii* + AGS-treated group: Mice were intragastrically administered 200 μL *K. pintolopesii* suspension (1×10^9^ CFU/mL) and 100 μL AGS solution (10 mg/kg body weight) twice for week and last 4 weeks.

The dosage of AGS was determined based on previous pre-experiments and relevant literature reports. During the 4-week intervention period, the general condition, body weight, and food/water intake of mice in each group were monitored daily. At the end of the intervention, mice were fasted for 12 h, anesthetized with 1% pentobarbital sodium (50 mg/kg body weight) via intraperitoneal injection, and sacrificed by cervical dislocation. Spleen tissues were rapidly dissected: part of the spleen was fixed in 4% paraformaldehyde for histopathological staining, and the remaining part was frozen in liquid nitrogen immediately and stored at -80°C for RNA sequencing and molecular experiments.

### 2.6 Histopathological staining and morphological analysis

#### 2.6.1 H&E staining

Fixed splenic tissues were dehydrated through a graded ethanol series (70%, 80%, 90%, 95%, 100% ethanol, 15 min each), cleared in xylene (twice, 15 min each), embedded in paraffin, and sectioned into 4 μm-thick slices using a microtome (Leica RM2255, Germany). Slices were deparaffinized with xylene (twice, 10 min each), rehydrated through a graded ethanol series (100%, 95%, 90%, 80%, 70% ethanol, 5 min each), and rinsed with distilled water for 5 min. Slices were stained with hematoxylin for 5 min, differentiated with 1% hydrochloric acid ethanol for 30 s, rinsed with tap water for 10 min to blue the nucleus, counterstained with eosin for 2 min, dehydrated, cleared in xylene, and mounted with neutral balsam.

The morphological structure of the spleen (white pulp, red pulp, lymphocyte density, inflammatory infiltration) was observed under a light microscope (Olympus BX53, Japan) at 200× magnification. Five non-overlapping random visual fields per slice were captured, and ImageJ software (v1.8.0, NIH, USA) was used to quantify the lymphocyte density (number of lymphocytes per mm²) and the ratio of white pulp area to total spleen area.

#### 2.6.2 Masson trichrome staining

Paraffin sections were deparaffinized and rehydrated as described above. Slices were stained with Weigert iron hematoxylin for 10 min, rinsed with distilled water for 5 min, stained with ponceau fuchsin for 15 min, differentiated with 1% phosphomolybdic acid for 10 min, rinsed with distilled water, stained with aniline blue for 5 min, dehydrated, cleared, and mounted. Collagen fiber deposition (blue staining) was observed at 200× magnification.

#### 2.6.3 Prussian blue iron staining

Paraffin sections were deparaffinized and rehydrated, then incubated with freshly prepared Prussian blue staining solution (2% potassium ferricyanide + 2% hydrochloric acid, 1:1) for 30 min at room temperature. Slices were rinsed with distilled water three times (5 min each), counterstained with nuclear fast red for 5 min, dehydrated, cleared, and mounted. Iron deposition (blue-green granules) was observed at 400× magnification.

### 2.7 Immunohistochemical staining

#### 2.7.1 Sample preparation and antigen retrieval

Paraffin-embedded spleen sections (4 μm-thick) were deparaffinized and rehydrated as described in 2.6.1. After rehydration, sections were incubated in 3% hydrogen peroxide (H_2_O_2_) solution at room temperature for 10 min to block endogenous peroxidase activity, then rinsed with PBS-T (PBS containing 0.05% Tween-20, pH 7.4) three times (5 min each). Antigen retrieval was performed by immersing sections in citrate buffer (10 mM sodium citrate, pH 6.0) and heating in a microwave oven (700 W) for 10 min, followed by natural cooling to room temperature and rinsing with PBS-T.

#### 2.7.2 Antibody incubation and color development

Sections were blocked with 5% bovine serum albumin (BSA) at room temperature for 30 min to reduce non-specific binding. Excess BSA was drained without rinsing, and sections were incubated overnight at 4°C in a humidified chamber with primary antibodies diluted in 1% BSA: anti-p16^INK4a^ (1:200, GB151143), anti-p21^Waf1/Cip1^ (1:250, GB155313), anti-GPX4 (1:300, GB154327), anti-TFR2 (1:200, GB113731), anti-ACSL4 (1:1000, GB155608), anti-FTH1 (1:200, GB115535), anti-IL-1β (1:1000, GB11113), anti-IL-17 (1:700, GB11110-1), anti-NF-kB p65 (1:2500, GB15997), anti-TNF-a (1:1000, GB115702), and rabbit IgG isotype control (1:200, negative control). After overnight incubation, sections were rinsed with PBS-T three times (5 min each), then incubated with HRP-conjugated goat anti-rabbit secondary antibody (1:500) at room temperature for 1 h. Sections were rinsed with PBS-T again, and color development was performed using DAB substrate kit (incubation for 3–5 min, monitored under a microscope to avoid over-staining). The reaction was terminated by rinsing with distilled water.

#### 2.7.3 Counterstaining and quantification

Sections were counterstained with hematoxylin for 30 s, differentiated with 1% hydrochloric acid ethanol for 10 s, rinsed with tap water to blue the nucleus, dehydrated, cleared, and mounted. Stained sections were observed under a light microscope at 200× and 400× magnifications. Five non-overlapping random visual fields per section were captured, and immunohistochemical images were analyzed using ImageJ software. The integrated optical density (IOD) of positive staining and the area of the region of interest (ROI) were measured. The mean optical density (MOD) was calculated as IOD/Area, which was used to represent the relative expression level of the target protein.

### 2.8 Multiplex immunofluorescence staining for CD4/S100A4/EGR1

#### 2.8.1 Sample preparation and antigen retrieval

Paraffin-embedded tissue sections (4 μm-thick) were deparaffinized and rehydrated as described in 2.7.1. After rehydration, sections were rinsed with PBS-T (PBS containing 0.05% Tween-20, pH 7.4) three times (5 min each). Antigen retrieval was performed by immersing sections in citrate buffer (10 mM sodium citrate, pH 6.0) and heating in a microwave oven (700 W) for 10 min, followed by natural cooling to room temperature and rinsing with PBS-T.

#### 2.8.2 Antibody incubation and fluorescent labeling

Sections were blocked with 5% bovine serum albumin (BSA) at room temperature for 30 min to reduce non-specific binding. Excess BSA was drained without rinsing, and sections were incubated overnight at 4°C in a humidified chamber with a cocktail of primary antibodies diluted in 1% BSA: anti-CD4 (1:200, green channel, GB15110), anti-S100A4 (1:300, red channel, GB11397), and anti-EGR1 (1:250, orange/yellow channel, GB11853), with matched isotype IgG included as negative control. After overnight incubation, sections were rinsed with PBS-T three times (5 min each), then incubated with fluorophore-conjugated secondary antibodies (Alexa Fluor 488, Cy3, Alexa Fluor 594, 1:500) at room temperature for 1 h in the dark.

#### 2.8.3 Nuclear counterstaining and imaging

Sections were rinsed with PBS-T again, then incubated with DAPI (4′,6-diamidino-2-phenylindole) at room temperature for 5 min to counterstain nuclei. After washing, sections were mounted with anti-fade mounting medium. Stained sections were observed under a fluorescence or confocal laser scanning microscope at 200× and 400× magnifications. Five non-overlapping random visual fields per section were captured, and the fluorescence intensity and co-localization of CD4, S100A4, and EGR1 were analyzed using ImageJ software, with DAPI as the nuclear reference.

### 2.9 TCR repertoire analysis (TRA vs. TRB)

Total RNA was extracted from splenic tissues of Control and KP groups (n=3 per group) using TRIzol reagent. RNA purity (A260/A280 = 1.8–2.0) and integrity (RNA integrity number, RIN ≥8.0) were verified using a NanoDrop 2000 spectrophotometer and an Agilent 2100 Bioanalyzer, respectively. cDNA was synthesized from 1 μg total RNA using a PrimeScript RT reagent kit. Multiplex PCR was performed to amplify V/J regions of TRA and V/D/J regions of TRB using specific primers. PCR products were purified to remove non-specific bands, then subjected to high-throughput sequencing on the Illumina NovaSeq 6000 platform (paired-end 150 bp).

Raw reads were filtered using Fastp (v0.23.0) to remove low-quality reads and adapters. Clean reads were aligned to the IMGT/V-QUEST database for TCR gene annotation and clonal identification (a clone was defined as a unique combination of V, D, J genes and CDR3 amino acid sequence; clones with frequency ≥0.1% were considered dominant). Bioinformatics analysis was conducted using the R package “immunarch” (v0.9.1) to evaluate TCR repertoire characteristics (CDR3 length distribution, V-J rearrangement, diversity indices, V gene subfamily usage, clonal size distribution). Statistical analysis was performed using GraphPad Prism 9.0 software.

### 2.10 Single-nucleus RNA sequencing and single-cell RNA sequencing

#### 2.10.1 Single-nucleus isolation and scRNA-seq library preparation

Frozen splenic tissues from the Control group and *K. pintolopesii* group were used. For each group, three mice were randomly selected, and their spleens were ground into powder in liquid nitrogen. Single nuclei were isolated using a Nuclear Isolation Kit (Miltenyi Biotec, Germany, Cat. No.: 130-109-198) following the manufacturer’s instructions. The nuclear suspension was filtered through a 40 μm cell strainer, and nuclear viability and concentration were determined using a hemocytometer and trypan blue staining (viability ≥ 85% required). For scRNA-seq of immune cells, splenic single-cell suspensions were prepared by mechanical dissociation and collagenase IV digestion (0.5 mg/mL, 37 °C, 30 min), and immune cells were enriched using a mouse immune cell isolation kit (Miltenyi Biotec, Germany).

Biological samples were processed individually: single-cell/nucleus suspensions were prepared and quality-controlled separately for each of the three randomly selected mice per group. After individual QC, equal numbers of cells/nuclei from the three QC-passed mice within each group were pooled to form one pooled sample per group, which was then loaded onto the Chromium Controller (10x Genomics) for independent library construction and sequencing. No individual mouse was sequenced separately. This pooling design averages inter-individual variation within each group. To address residual technical batch effects, the Control and KP libraries were processed and sequenced in parallel, and the Harmony algorithm (v0.1.5) integrated within the Seurat (v4.3.0) pipeline was applied for cross-dataset integration, with batch correction verified by pre- and post-correction PCA plots. It should be noted that this pooling strategy generated only one pooled library per condition and therefore does not provide independent animal-level biological replication; accordingly, the single-cell/single-nucleus analyses should be regarded as primarily exploratory, intended to identify candidate cell states and pathways rather than to provide rigorous group-level statistical inference.

Single-nucleus/cell RNA sequencing libraries were constructed using the Chromium Single Cell 3′ Reagent Kit v3.1 (10x Genomics, USA) following the manufacturer’s protocol. Briefly, single nuclei/cells were loaded onto a Chromium Controller to generate single-cell gel beads-in-emulsion (GEMs), followed by reverse transcription to synthesize cDNA, cDNA amplification, library construction, and quality control. Library quality was assessed using an Agilent 2100 Bioanalyzer (Agilent, USA), and qualified libraries were sequenced on an Illumina NovaSeq 6000 platform (Illumina, USA) with a sequencing depth of ~50,000 reads per nucleus/cell.

#### 2.10.2 Bulk RNA-seq library preparation and sequencing

Total RNA was extracted from splenic tissues of the Control group, *K. pintolopesii* group, and *K. pintolopesii* +AGS group (n=3 per group) using TRIzol Reagent following the manufacturer’s instructions. RNA purity, concentration, and integrity were detected using a NanoDrop 2000 spectrophotometer (Thermo Fisher Scientific, USA) and an Agilent 2100 Bioanalyzer (RNA integrity number, RIN ≥8.0 required). Bulk RNA-seq libraries were constructed using the TruSeq Stranded mRNA LT Sample Prep Kit (Illumina, USA), and qualified libraries were sequenced on an Illumina NovaSeq 6000 platform with a paired-end sequencing read length of 150 bp (PE150).

### 2.11 Bioinformatics analysis

#### 2.11.1 SnRNA-seq/scRNA-seq data processing

Raw sequencing data were filtered to remove low-quality reads, adapter sequences, and rRNA reads using Fastp (v0.23.0) (41, 42) with parameters: Q20 ≥95%, Q30 ≥90%, and GC content 40–60%. Clean reads were aligned to the mouse reference genome (Mus musculus, GRCm38) using STAR (v2.7.10a) (43). Cell barcode and UMI counting were performed using Cell Ranger (v7.1.0), and low-quality cells/nuclei were filtered out with criteria: number of genes per cell 200–5000, UMI count per cell <50,000, and mitochondrial gene ratio <10%.

Uniform Manifold Approximation and Projection (UMAP) was used for dimensionality reduction and cell clustering, and cell types were annotated based on lineage-specific marker genes using Seurat (v4.3.0) software (44). Pseudotime trajectory analysis was performed to explore cell differentiation dynamics using Monocle3 (v1.2.0) software (45). Differential gene expression (DEG) analysis between groups was performed using the Wilcoxon rank-sum test, with screening criteria: |log2 fold change (FC)| ≥1 and adjusted *p* < 0.05.

#### 2.11.2 Bulk RNA-seq data processing

Raw sequencing data were filtered using Fastp, and clean reads were aligned to the GRCm38 reference genome using HISAT2 (v2.2.1) (46). Gene expression levels were quantified as Fragments Per Kilobase of transcript per Million mapped reads (FPKM) using StringTie (v2.2.1). DEGs between groups were screened using DESeq2 (v1.38.0) with criteria: |log2FC| ≥1 and *p* < 0.05. Principal component analysis (PCA) and hierarchical clustering were performed to evaluate the repeatability and separation between groups.

#### 2.11.3 Functional enrichment analysis

GO (Gene Ontology) (47) and KEGG (Kyoto Encyclopedia of Genes and Genomes) (48) pathway enrichment analyses of DEGs were performed using clusterProfiler (v4.6.0) software with the criterion of adjusted *p* < 0.05. Wikipathway analysis was performed using the Wikipathways database (https://www.wikipathways.org/), and ferroptosis/ferro-aging-related pathway analysis was conducted based on the latest literature reports and the MsigDB database. GSEA was conducted against the Molecular Signatures Database (MSigDB) with a normalized enrichment score (NES) and nominal p-value to evaluate pathway enrichment. Heatmaps of representative DEGs were generated using pheatmap with a significance threshold of *p* < 0.05.

### 2.12 *In silico* virtual knockout analysis

To infer the regulatory roles of senescence-associated transcription factors (TFs) *Atf3*, *Bach2*, and *Elf1* in splenic B cell dysfunction, in silico virtual knockout was performed based on bulk RNA-seq data. A gene regulatory network (GRN) was constructed using normalized expression matrices, with TF-target gene interactions inferred via mutual information and elastic net regression (α = 0.5). Virtual knockout was simulated by nullifying the expression of target TFs, and differentially expressed genes (DEGs) were defined as those with a predicted fold change (FC) ≥ 2 or ≤ 0.5 and false discovery rate (FDR) < 0.05. Top perturbed genes and functional enrichment analyses (GO/KEGG) were conducted to identify core regulatory programs mediated by each TF.

### 2.13 Cell-type deconvolution analysis

Cell-type deconvolution of bulk RNA-seq data was performed using the CIBERSORTx algorithm with a mouse splenic immune cell signature matrix (adapted from the LM22 signature set). The analysis was run in absolute mode with 1000 permutations to assess statistical significance, aiming to estimate the relative abundance of 12 major immune cell subsets (including B cells, T cells, neutrophils, monocytes, plasma cells, erythrocytes, and erythroid precursors). The resulting cell subset proportions were used for subsequent comparative analysis between experimental groups.

### 2.14 Statistical analysis

All experimental data were expressed as the mean ± standard deviation (x ± s) and analyzed using SPSS 26.0 software (IBM, USA) and R software (v4.3.1). Graphs were generated using GraphPad Prism 9.0 software. Differences between two groups were compared using the independent samples t-test, and differences among multiple groups were compared using one-way analysis of variance (ANOVA) followed by the LSD *post-hoc* test. *p* < 0.05 was considered statistically significant.

## 3 Results

### 3.1 Single-cell transcriptomic atlas of mouse spleen and cell subpopulation shifts following *K. pintolopesii* intervention

To dissect the cellular and molecular landscape of splenic aging and its response to *K. pintolopesii* intervention, Single-cell RNA sequencing (scRNA-seq) was performed on spleen tissues from aged mice. After stringent quality control, uniform manifold approximation and projection (UMAP) analysis resolved 11 discrete cell clusters in the integrated dataset (**Figure 1A–a**), encompassing erythrocytes, erythroid progenitor cells, plasmacytoid dendritic cells, neutrophils, mononuclear phagocytes, proliferating cells, plasma cells, B cells, T cells, natural killer (NK) cells, and doublets. Separate UMAP visualization of the control and *K. pintolopesii*-treated groups confirmed a conserved clustering architecture (**Figures 1A–b, c**), ensuring valid cross-group comparisons.

**Figure 1:**
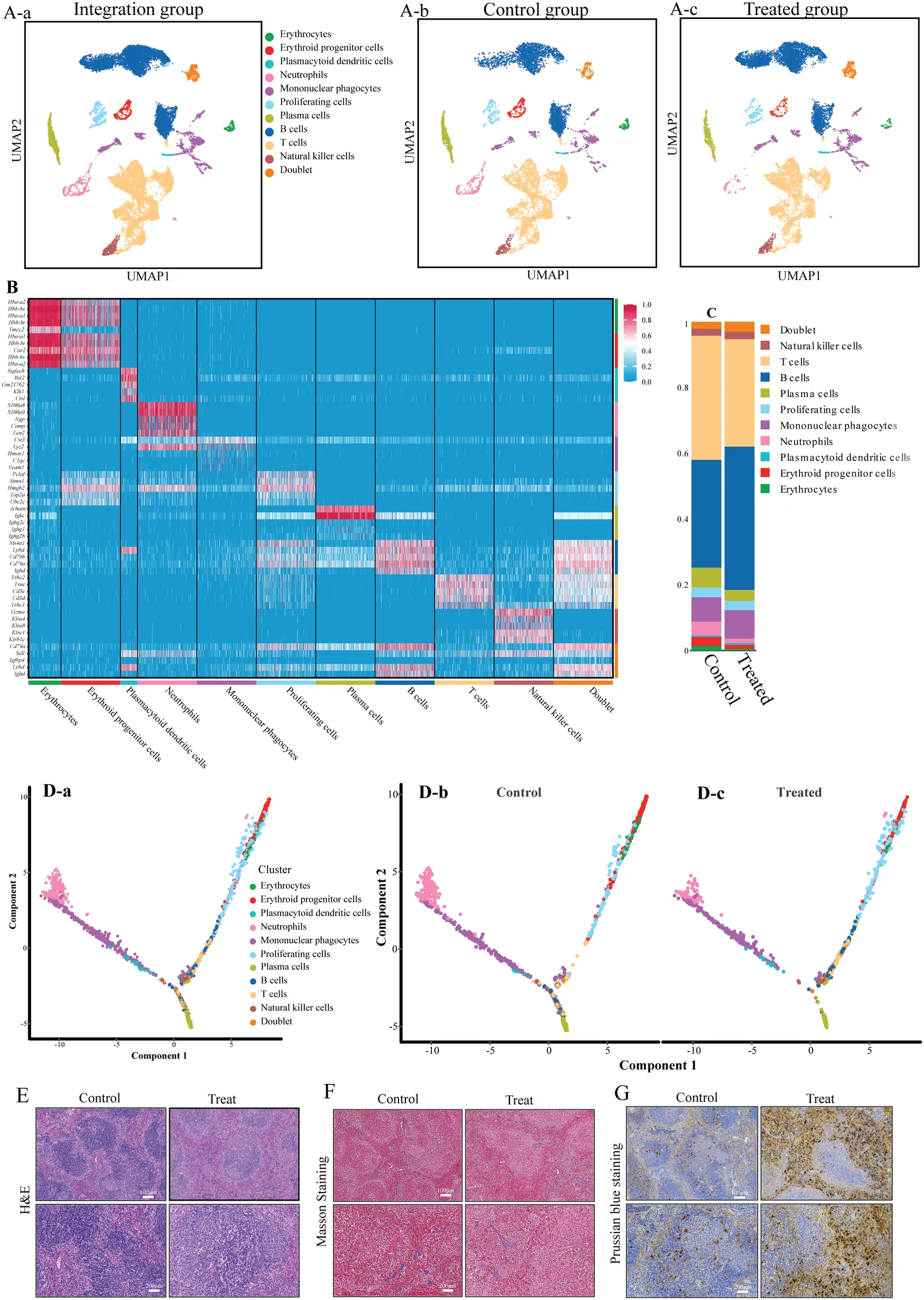
Single-nucleus RNA sequencing (snRNA-seq) reveals the splenic cellular atlas and histopathological changes in aged mice following *K. pintolopesii* intervention. **(A)** UMAP visualization of integrated splenic cell clusters from control and *K. pintolopesii*-treated mice **(a)**, and separate UMAP plots for the control group **(b)** and *K. pintolopesii*-treated group **(c)**; 11 major cell clusters are annotated. **(B)** Heatmap of lineage-specific marker genes for each splenic cell cluster, validating cell type identity. **(C)** Proportional distribution of major splenic immune cell subsets in the control and *K. pintolopesii*-treated groups. **(D)** Pseudotime trajectory analysis of splenic cells from control and *K. pintolopesii*-treated mice, showing altered differentiation dynamics upon fungal intervention. **(E)** H&E staining of splenic tissues (200×) showing white pulp lymphocyte rarefaction and mild inflammatory infiltration in the *K. pintolopesii*-treated group compared with the control group; scale bar = 50 μm. **(F)** Masson trichrome staining (200×) showing mild collagen fiber hyperplasia in the red pulp and white pulp marginal zones of *K. pintolopesii*-treated mice; scale bar = 50 μm. **(G)** Prussian blue iron staining (400×) showing prominent iron granule accumulation in *K. pintolopesii*-treated splenic tissues (blue-green granules); scale bar = 20 μm. Data are presented as mean ± SD, *p* < 0.05 vs. control group.

A heatmap of lineage-specific marker genes validated the identity of each cell cluster (**Figure 1B**). Erythrocytes selectively expressed hemoglobin genes (e.g., *Hba-a1*, *Hbb-bs*), T cells exhibited high expression of T cell receptor complex components (e.g., *Cd3d*, *Cd3e*), B cells were defined by B cell antigen receptor-associated genes (e.g., *Cd79a*, *Cd79b*), and mononuclear phagocytes expressed myeloid lineage determinants (e.g., *Cd14*, *Csf1r*). These lineage-restricted expression profiles confirmed accurate cell-type annotation, establishing a robust cellular atlas for downstream analyses.

Quantification of the splenic immune landscape revealed minimal compositional shifts following *K. pintolopesii* intervention (**Figure 1C**). In the control group, B cells were the most abundant population (~58%), followed by T cells (~37%), while doublets constituted only a small fraction (<5%) of total cells. Treatment with *K. pintolopesii* led to a slight increase in the B cell proportion (~62%) and a minor reduction in T cells (~32%), with no substantial changes in the relative abundance of doublets. The fractions of other lineages, including natural killer cells, mononuclear phagocytes, neutrophils, and plasmacytoid dendritic cells, remained low and largely unchanged between groups. These results indicate that, at the global lineage level, *K. pintolopesii* induces only modest compositional shifts in the splenic immune compartment. However, as detailed in subsequent sections, subpopulation-level analysis within individual immune cell lineages (T cells, B cells, myeloid cells) revealed profound remodeling, indicating that *K. pintolopesii* exerts lineage-specific rather than global compositional effects.

Pseudotime trajectory analysis revealed a Y-shaped branching topology rooted in erythroid lineage cells and extending toward terminally differentiated immune populations (**Figure 1D**). Control cells exhibited a broad, dispersed trajectory, reflecting dysregulated age-related cellular dynamics. In contrast, *K. pintolopesii*-treated cells displayed a compact, converged trajectory with smoother state transitions, reflecting pathological clonal convergence and reduced cellular heterogeneity rather than beneficial stabilization - a pattern consistent with immune aging and loss of repertoire diversity.

To further characterize the molecular changes underlying *K. pintolopesii*-mediated immune modulation, we performed subpopulation-specific gene regulation and differential expression analyses. Violin plots of cluster-specific gene expression (**Supplementary Figure 1A**) and a heatmap of top cluster-defining genes (**Supplementary Figure 1B**) confirmed the stability of cell-type identity across groups, while regulon specificity scoring revealed distinct regulatory programs in control and treated subsets (**Supplementary Figure 1C**). Notably, genes such as *Spi1*, *Irf8*, and *Gata1* exhibited altered specificity scores following treatment (**Supplementary Figure 1D**), suggesting rewiring of lineage-specific transcriptional networks.

Volcano plot analysis (**Supplementary Figure 1E**) identified 238 significantly differentially expressed genes (DEGs) between control and treated spleens. Genes involved in MHC class II antigen processing and presentation (e.g., *H2-DMb2*, *H2-Eb1*, *H2-Ab1*, *H2-Aa*, *Cd74*) were prominently upregulated, alongside genes encoding antimicrobial peptides (e.g., *Camp*, *Ltf*, *Lcn2*, *S100a8*, *S100a9*) and immunoglobulin heavy chains (e.g., *Ighg1*, *Ighg2c*). Conversely, genes associated with immunoregulation and lipid metabolism (e.g., *Apoe*, *Ccl4*, *Cd72*) were downregulated. Gene Ontology (GO) enrichment analysis (**Supplementary Figure 1F**) revealed that these DEGs were highly enriched in biological processes related to antigen processing and presentation, cellular components including endocytic vesicles and MHC class II protein complexes, and molecular functions involving peptide binding and cysteine-type peptidase activity. Disease association analysis (**Supplementary Figure 1G**) further linked these DEGs to pathways involved in tuberculosis, autoimmune thyroid disease, and rheumatoid arthritis, suggesting a connection between *K. pintolopesii*-induced immune activation and chronic inflammatory senescence phenotypes.

Cell–cell interaction network analysis (**Supplementary Figure 1H**) revealed that *K. pintolopesii* treatment reshaped intercellular communication patterns, with enhanced interactions between immune subsets (e.g., T cells, NK cells, and plasmacytoid dendritic cells) and erythroid lineage cells, consistent with the observed expansion of immune surveillance populations and stabilized differentiation trajectories. Collectively, these findings indicate that *K. pintolopesii* induces a robust and persistent immune response characterized by enhanced antigen presentation and chronic inflammation, which likely is associated with the observed splenic senescence-like changes.

### 3.2 Histopathological changes in the spleen tissues of aged mice following *K. pintolopesii* intervention

Hematoxylin and eosin (H&E) staining revealed clear demarcation between white and red pulp in control spleens, with dense lymphocyte aggregates in the white pulp and intact splenic cords and sinuses in the red pulp (**Figure 1E**). *K. pintolopesii*-treated spleens exhibited reduced lymphocyte density and loose cellular arrangement in the white pulp, accompanied by mild inflammatory cell infiltration in the red pulp; the overall histological structure remained intact, with no significant disruption or necrosis observed.

Masson’s trichrome staining showed minimal delicate blue collagen fibers around blood vessels and beneath the splenic capsule in control mice (**Figure 1F**). In *K. pintolopesii*-treated mice, collagen fiber distribution and density were slightly increased, particularly in the red pulp and white pulp marginal zones, though only mild interstitial hyperplasia was present, with no evidence of extensive fibrotic scarring.

Prussian blue staining demonstrated scattered blue-green iron granules within red pulp macrophages in control spleens, consistent with physiological iron storage (**Figure 1G**). *K. pintolopesii*-treated spleens showed significantly increased iron granule accumulation and enhanced staining intensity, with deposits extending to the white pulp marginal zones, indicating prominent splenic iron overload.

These findings together suggested that *K. pintolopesii* intervention induced mild splenic pathological changes in aged mice, characterized by white pulp lymphocyte rarefaction, mild interstitial collagen hyperplasia, and prominent iron deposition, without severe structural impairment.

### 3.3 *K. pintolopesii* remodels T cell subset composition and promotes effector/memory differentiation in the aging spleen

scRNA-seq was performed to characterize the landscape of splenic T cells in control and *K. pintolopesii*-treated aged mice. UMAP dimensionality reduction (**Figure 2A**) resolved eight distinct T cell subsets: naive CD4^+^/CD8^+^ T cells, effector/memory CD4^+^/CD8^+^ T cells, regulatory T cells (Tregs), follicular helper T cells (Tfh), and NKT cells. A marker gene heatmap (**Figure 2C**) validated subset identities via canonical lineage markers: naive T cells expressed *Ly6c1* and *Sell*; effector CD8^+^ T cells expressed *Gzmk* and *Klrk1*; Tregs expressed *Tnfrsf4*; Tfh cells expressed *Pdcd1*; and NKT cells expressed *Cxcr6*.

**Figure 2:**
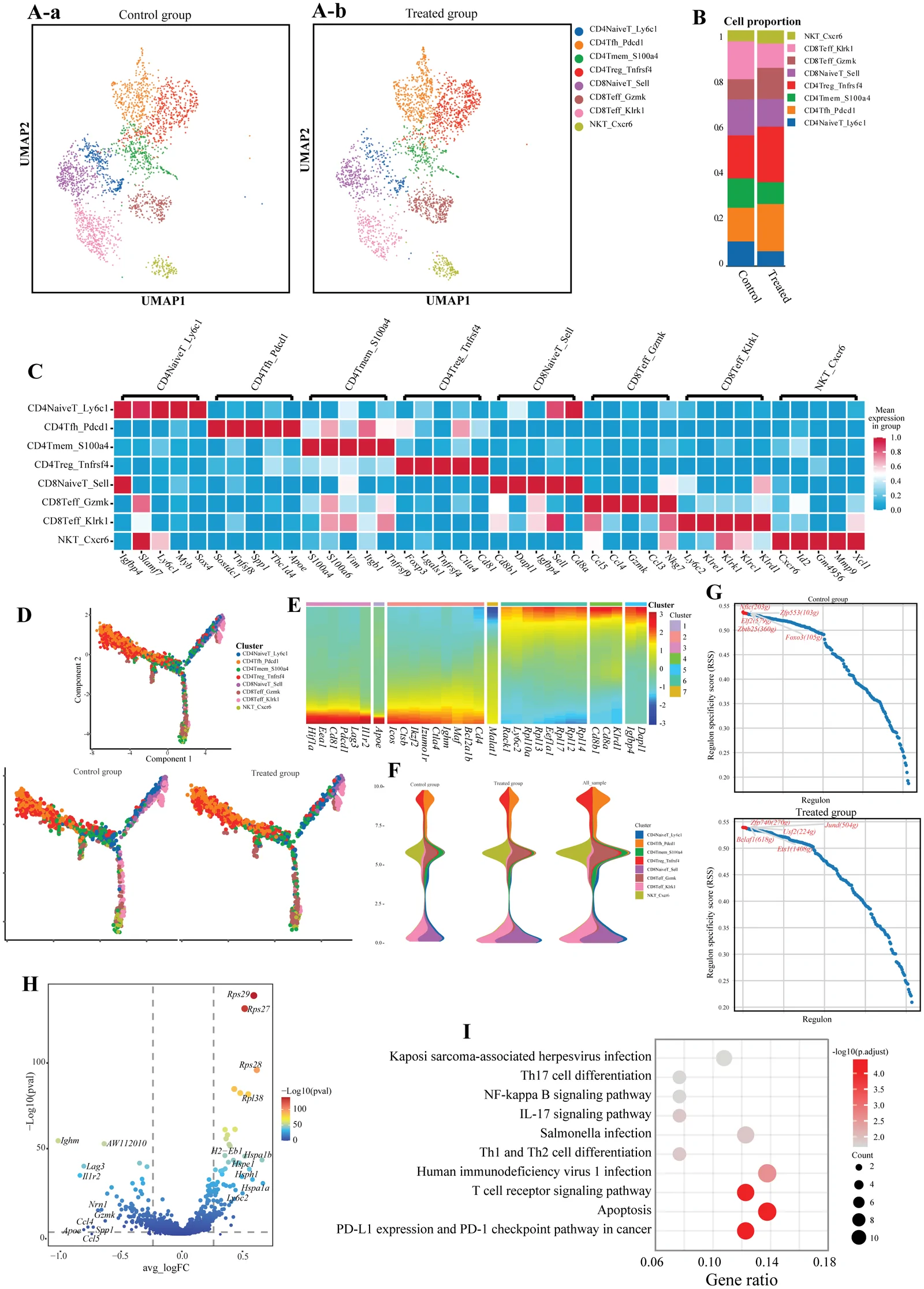
K*. pintolopesii* remodels splenic T cell subsets and accelerates naive T cell differentiation into effector/memory phenotypes in aged mice. **(A)** UMAP visualization of 8 distinct splenic T cell subsets identified by scRNA-seq in control and *K. pintolopesii*-treated mice. **(B)** Proportional changes in T cell subsets following *K. pintolopesii* intervention, showing depletion of naive T cells and expansion of effector/memory subsets. **(C)** Heatmap of canonical marker genes for each T cell subset, validating subset annotation. **(D)** Pseudotime trajectory analysis of T cells, revealing a rewired differentiation hierarchy in *K. pintolopesii*-treated mice. **(E)** Dynamic expression of naive and effector/memory T cell signature genes along the pseudotime trajectory. **(F)** Distribution of T cell subsets along the pseudotime trajectory, with naive T cells in early stages and effector/memory subsets in late stages. **(G)** Transcription factor (TF) regulon activity analysis, showing enhanced activity of activation-associated TFs (*Egr1*, *Jun*, *Fos*) in *K. pintolopesii*-treated T cells. **(H)** Volcano plot of differentially expressed genes (DEGs) in T cells between control and *K. pintolopesii*-treated groups (|log_2_FC| ≥1, adjusted *p* < 0.05). **(I)** KEGG pathway enrichment analysis of upregulated DEGs in *K. pintolopesii*-treated T cells, highlighting immune activation and inflammation-related pathways.

Subset proportion analysis (**Figure 2B**) revealed profound remodeling of T cell homeostasis following *K. pintolopesii* treatment: relative to controls, naive CD4^+^ T cells (CD4NaiveT_Ly6c1) were markedly depleted, while effector/memory subsets expanded significantly, including CD4^+^ memory T cells (CD4Tmem_S100a4), effector CD8^+^ T cells (CD8Teff_Gzmk, CD8Teff_Klrk1), Tregs (CD4Treg_Tnfrsf4), and NKT cells (NKT_Cxcr6). These data indicate that *K. pintolopesii* drives aging splenic T cells from a quiescent naive state toward an activated effector/memory phenotype.

Pseudotime trajectory analysis (**Figure 2D**; **Supplementary Figure 2A**) uncovered a Y-shaped bifurcation representing two primary differentiation paths. In the control group (**Supplementary Figures 2A**, left), the trajectory origin was dominated by CD4Treg_Tnfrsf4 and CD4Tfh_Pdcd1, with naive T cells (CD4NaiveT_Ly6c1, CD8NaiveT_Sell) confined to terminal segments, reflecting a homeostatic, quiescent differentiation hierarchy. In contrast, *K. pintolopesii*-treated cells (**Supplementary Figures 2A**, right) exhibited a rewired trajectory, as the origin shifted to CD4Tmem_S100a4 and NKT_Cxcr6, while naive T cells were integrated into a continuous differentiation chain linking NKT cells, effector CD8^+^ T cells (CD8Teff_Klrk1), and naive T cells. Treated cells were enriched in mid-to-late trajectory segments, confirming accelerated differentiation from naive to effector/memory states. Dynamic gene expression along pseudotime (**Figure 2E**) validated this transition: naive T cell-associated genes (*Ly6c1*, *Sell*, *Ccr7*) were downregulated, while effector/memory signature genes (*Gzmk*, *S100a4*, *Tnfrsf4*, *Ifng*) were upregulated. Subset distribution along pseudotime (**Figure 2F**) further confirmed this hierarchy, with naive T cells occupying early stages and effector/memory subsets occupying late stages. Region-specific gene expression analysis (**Figure 2G**) showed control cells enriched in quiescence-related genes (*Nkg7*, *Ccl5*), while treated cells activated immediate-early genes (*Egr1*, *Jun*, *Fos*), corroborating T cell activation.

To dissect intercellular communication and regulatory changes, we analyzed cell-cell interaction networks and ligand-receptor signaling (**Supplementary Figure 2**). Cell-cell interaction chord diagrams (**Supplementary Figure 2B**) revealed that *K. pintolopesii* treatment reshaped the splenic immune communication landscape: interactions between naive T cells and other myeloid/lymphoid subsets were weakened, while interactions between effector/memory T cells, Tregs, NKT cells, and proliferating cells were markedly enhanced. Interaction counts networks (**Supplementary Figure 2E**) confirmed this shift, demonstrating increased connectivity among activated T cell subsets, particularly between effector CD8^+^ T cells and Tregs, and between NKT cells and memory CD4^+^ T cells.

Ligand-receptor interaction heatmaps (**Supplementary Figures 2C, D**) further validated these changes: in the treated group, pro-inflammatory signaling pathways (e.g., *Tnf*-*Tnfrsf1b*, *Ifng*-*Ifngr1*, *Ltb*-*Ltbr*) and T cell co-stimulation (e.g., *Cd28*-*Cd80/CD86*, *Icos*-*Icosl*) were significantly upregulated, alongside enhanced expression of immune checkpoint molecules (e.g., *Pdcd1*-*Cd274*, *Ctla4*-*Cd80/CD86*, *Lag3*-*MhcII*). Additionally, integrin-mediated adhesion and extracellular matrix remodeling pathways (e.g., *Itga4*-*Icam1*, *Itgb1*-*Fn1*) were activated, indicating enhanced T cell migration and tissue residency. Transcription factor (TF) regulon analysis (**Figure 2G**) identified enhanced activity of activation-associated TFs (*Egr1*, *Jun*, *Fos*, *Atf3*) in treated cells, consistent with the observed T cell effector/memory transition and rewired intercellular communication.

Differential gene expression and functional enrichment analyses (**Figures 2H, I**; p < 0.05) showed that *K. pintolopesii* upregulated numerous genes, including Rgs family members Rgs29, Rgs27, and Rgs28, associated with core immune activation pathways (Th17 cell differentiation, NF-κB signaling, IL-17 signaling, TCR signaling), immune checkpoint regulation (PD-L1/PD-1), and apoptosis pathways. These findings suggest that *K. pintolopesii* activates T cell effector programs in aging T cells, which may initially enhance immune responsiveness but ultimately drives exhaustion and senescence through chronic activation, while concurrently engaging checkpoint and apoptotic mechanisms as compensatory feedback.

These scRNA-seq data suggest that *K. pintolopesii* remodels the composition of aging splenic T cell subsets, accelerates naive T cell differentiation toward effector/memory phenotypes, and activates key immune signaling pathways. Notably, this sustained T cell activation may contribute to age-related immune exhaustion and chronic inflammation, thereby exacerbating splenic aging. The rewired intercellular communication and enhanced checkpoint signaling further highlight a complex adaptive response that balances immune activation with homeostatic restraint in the aged spleen, potentially linking microbial perturbation to T cell senescence and functional decline.

Regarding the apparent discrepancy between the scRNA-seq data (**Figure 2B**), which showed expansion of the CD4Tmem_S100a4 subset, and the immunofluorescence data (**Figures 3D, E**), which showed reduced per-cell protein expression of CD4, S100A4, and EGR1 in the treated group: these observations are not contradictory. The scRNA-seq captures the proportional expansion of this subset at the transcriptomic level, while the IF analysis measures per-cell protein expression intensity. The expanded CD4Tmem_S100a4 population in KP-treated mice may exhibit reduced per-cell protein expression of these markers, a phenomenon characteristic of T cell exhaustion where cells proliferate but lose functional protein expression (‘paralyzed expansion’). Additionally, transcript-protein divergence due to post-transcriptional regulation may contribute to this pattern. This interpretation is consistent with the known biology of chronically activated T cells in aging.

**Figure 3:**
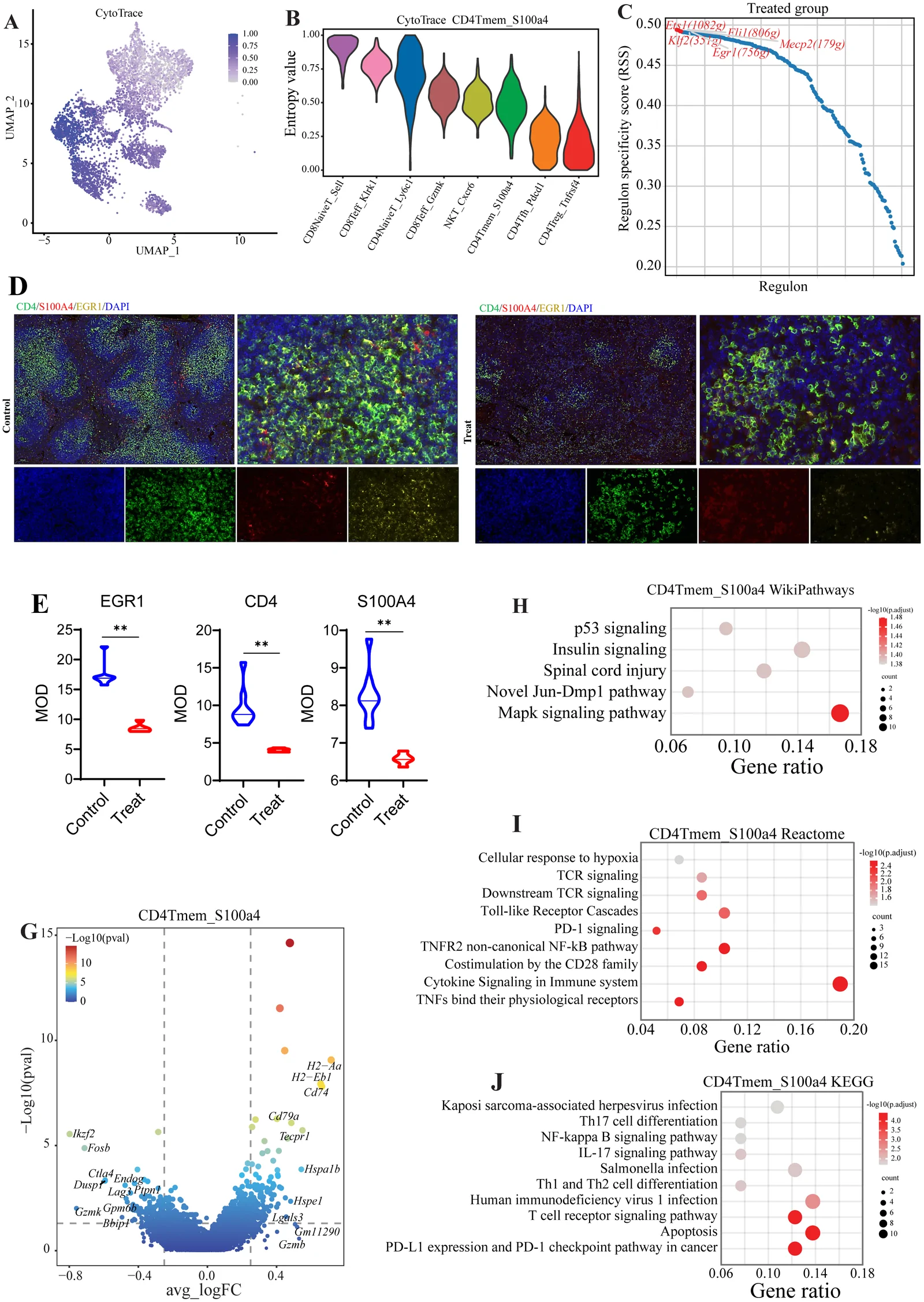
Single-cell transcriptomic profiling of the transitional CD4^+^ memory T cell subset (CD4Tmem_S100a4). **(A)** UMAP projection showing CytoTRACE-predicted differentiation potential across all T cells, with color gradients from high (blue) to low (purple). **(B)** Violin plots of CytoTRACE entropy across T cell subsets, ordered by increasing entropy (decreasing differentiation potential). CD4Tmem_S100a4 occupies an intermediate position between naive and terminally differentiated subsets, indicating a transitional state. **(C)** Regulon specificity score (RSS) ranking showing top transcription factors (TFs) with highest specificity for CD4Tmem_S100a4, including *Ets1*, *Fli1*, *Klf2*, *Egr1*, and *Mecp2*, with target gene numbers in parentheses. **(D)** Representative immunofluorescence staining of splenic tissues showing CD4 (green), S100A4 (yellow), and EGR1 (red) expression in control vs. treated mice, scale bars = 50 μm. **(E)** Quantitative analysis of MOD (mean optical density) for EGR1, CD4, and S100A4, confirming significant downregulation in the treated group (*p* < 0.01). **(F)** Volcano plot of DEGs in CD4Tmem_S100a4 (vs. other subsets), highlighting key markers of memory (*S100a4*), activation (*Cd74*, *Cd79a*), antigen presentation (*H2-Aa*, *H2-Eb1*), and reduced effector function (low *Gzmk*/*Gzmb*). **(G–I)** Bubble plots of pathway enrichment in CD4Tmem_S100a4 using WikiPathways **(G)**, Reactome **(H)**, and KEGG **(I)**. Enriched pathways include TCR signaling, NF-κB activation, Th1/Th2 differentiation, PD-1 checkpoint signaling, and apoptosis, supporting the subset’s transitional and immunomodulatory roles.

### 3.4 Receptor repertoire remodeling upon treatment (TRA vs. TRB)

The CDR3 length distribution and TR V-J rearrangement patterns of TRA and TRB repertoires were first analyzed to evaluate the overall remodeling characteristics upon treatment (**Figures 4A, B**). For CDR3 length distribution (**Figure 4A**), the Control group exhibited a broad-peaked and long-tailed distribution (20–60 bp) with a peak at 38–40 bp, where others clones accounted for the absolute majority and dominant clones showed extremely low proportions, reflecting a state of high clonal diversity under basal conditions. In contrast, the Treated group displayed a similarly centered CDR3 length distribution (peak still at 38–40 bp), but with a substantial increase in the proportion of dominant clones and a concomitant decrease in others clones, indicating a shift toward focused clonal expansion induced by treatment without altering the inherent CDR3 length preference. Regarding TR V-J rearrangement patterns (**Figure 4B**), the Control group showed scattered and uniform V-J rearrangement connections without dominant pairings, reflecting the random diversity of V(D)J rearrangement. In the Treated group, however, specific V-J pairings were significantly amplified and concentrated to form dominant rearrangement clusters (e.g., TRAV13-2-TRAJ37 and TRBV13-3-associated pairings), suggesting a biased shift in V-J rearrangement and oligoclonalization of the repertoire after treatment.

**Figure 4:**
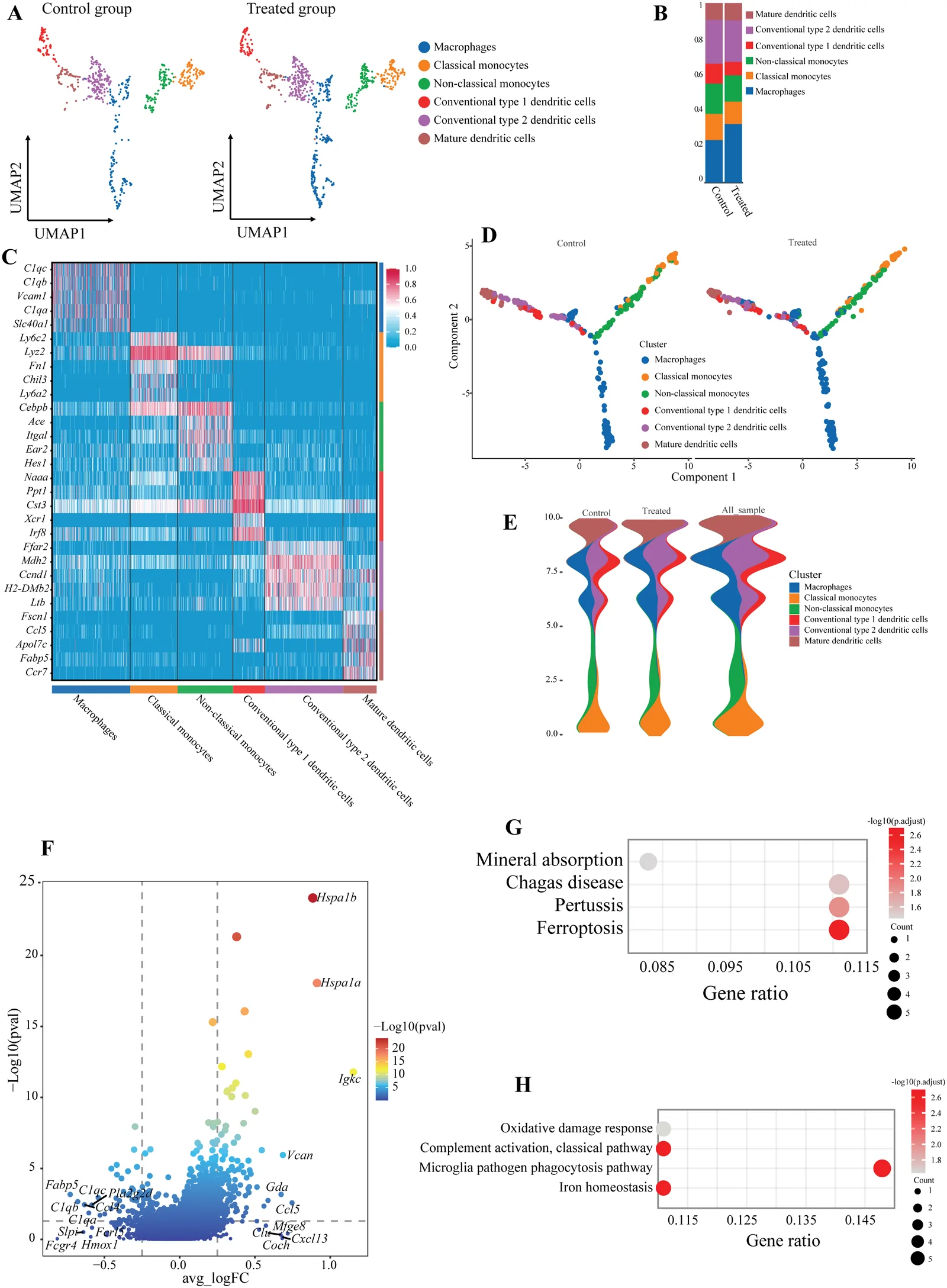
K*. pintolopesii* induces remodeling of the splenic TCR (TRA and TRB) repertoire in aged mice. **(A)** CDR3 length distribution of TRA and TRB repertoires in control and *K. pintolopesii*-treated groups, showing a shift toward dominant clone expansion in the treated group. **(B)** TR V-J rearrangement heatmaps for TRA and TRB, revealing concentrated, dominant V-J pairings in *K. pintolopesii*-treated mice compared with scattered pairings in controls. **(C)** Diversity indices (Chao1, D50/D60, Inverse Simpson) and Hill number curves for TRA and TRB repertoires, showing enhanced clonal richness but reduced overall diversity in the treated group. **(D)** Clonal abundance stratification of TRA and TRB repertoires, showing expansion of high-frequency clones (≥10/Large) and reduction of single-copy clones in *K. pintolopesii*-treated mice. **(E)** Clonal size distribution analysis, confirming the enrichment of large clones and depletion of small/medium clones in the treated group, with a more pronounced effect in TRB.

To quantify the clonal diversity of TRA and TRB repertoires, multiple diversity indices were calculated (**Figure 4C**). For both TRA (top row) and TRB (bottom row), the Chao1 and D50/D60 indices were significantly higher in the Treated group compared with the Control group, indicating enhanced clonal richness (total clone count) and evenness (more uniform distribution of non-dominant clones) following treatment. In contrast, the inverse Simpson index was consistently lower in the Treated group for both T cell receptor chains, which reflects a reduction in overall repertoire diversity driven by the expansion of high-frequency clones. Consistently, Hill number curves revealed that while Treated group showed higher diversity at q=0 (richness), it exhibited lower diversity at q≥2 (Simpson index), confirming a distinct shift toward oligoclonality. Notably, the magnitude of changes in these diversity indices was more pronounced in TRB than in TRA, suggesting that the β-chain is more sensitive to treatment-induced clonal selection.

Subsequently, clonal abundance stratification was analyzed (**Figures 4D, E**). In the Control group, single-copy (Unique/Single) clones dominated both TRA and TRB repertoires, with high-frequency clones (≥10/Large) representing a minor fraction. In sharp contrast, the Treated group exhibited a marked expansion of high-frequency clones (≥10/Large), accompanied by a reduction in the proportion of single-copy clones and an increase in low-abundance novel clones (black others category). This shift was more evident in TRB, where the expansion of large clones was far more dramatic than in TRA, indicating a stronger proliferative response of β-chain-bearing clones. Concurrently, V gene subfamily usage was biased toward specific subtypes in the Treated group, with a contraction of the others clone pool and enrichment of particular V gene segments, again a more pronounced effect was observed in TRB.

Clonal size distribution analysis (**Figure 4E**) further validated these observations: small clones (Single) dominated both repertoires in the Control group, while treatment led to a significant increase in large clones and a reduction in medium/small clones. This shift was most striking in TRB, where large clones became a major component of the repertoire. Collectively, these results demonstrate that treatment drives a selective remodeling of the TCR repertoire, characterized by enhanced clonal richness, reduced overall diversity, and oligoclonal expansion of specific clones, with the TRB repertoire exhibiting a more sensitive and robust response to treatment-induced clonal selection, ultimately becoming the dominant clonal population in the remodeled TCR landscape.

### 3.5 Remodeling of splenic myeloid cell subpopulation composition following *K. pintolopesii* intervention

scRNA-seq of splenic myeloid cells resolved six distinct clusters (**Figure 5A**), including macrophages, classical monocytes, non-classical monocytes, conventional type 1 dendritic cells (cDC1), conventional type 2 dendritic cells (cDC2), and mature dendritic cells (mDCs). A marker gene heatmap (**Figure 5C**) validated cell types via canonical lineage-defining genes, as *Cd68* (macrophages), *Ly6c2* (classical monocytes), *Ace* (non-classical monocytes), *Xcr1/Clec9a* (cDC1/cDC2), and *Cd80* (mDCs).

**Figure 5:**
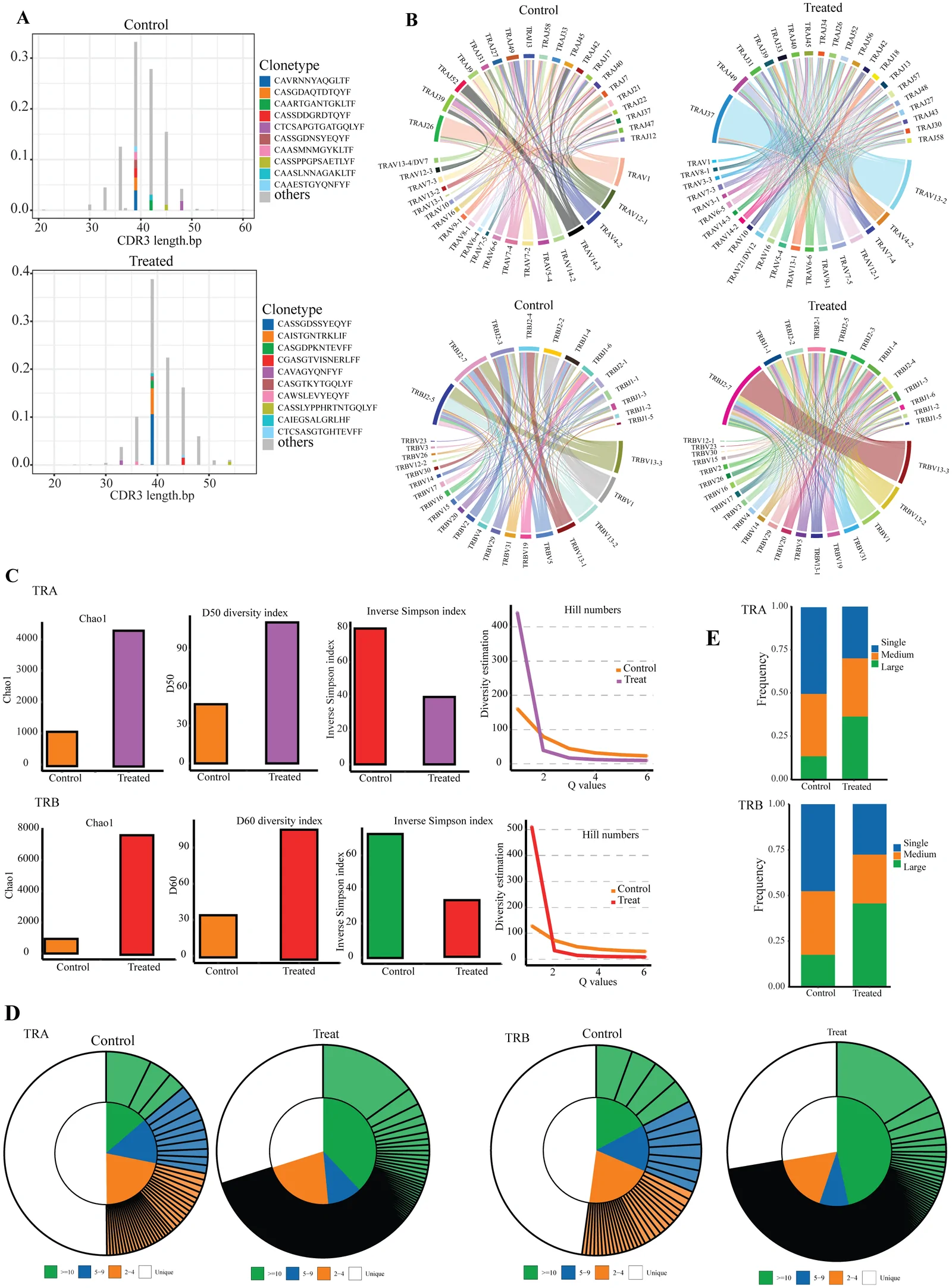
K*. pintolopesii* modulates splenic myeloid cell subset composition and activates ferroptosis in aged mice. **(A)** UMAP visualization of 6 distinct splenic myeloid cell subsets identified by scRNA-seq in control and *K. pintolopesii*-treated mice. **(B)** Proportional changes in myeloid subsets, showing increased macrophages/non-classical monocytes and depleted classical monocytes in the treated group. **(C)** Heatmap of canonical marker genes for each myeloid subset, validating cell type identity. **(D)** Pseudotime trajectory analysis, showing preserved myeloid developmental lineages with altered subset frequency in the treated group. **(E)** Violin plots of core lineage marker gene expression, confirming retained transcriptional identity across groups. **(F)** Volcano plot of DEGs in myeloid cells between control and *K. pintolopesii*-treated groups (|log_2_FC| ≥1, adjusted *p* < 0.05). **(G)** KEGG pathway enrichment analysis of DEGs, identifying ferroptosis as the top enriched pathway. **(H)** Wikipathway analysis, highlighting enrichment of oxidative damage response, iron homeostasis, and complement activation pathways in treated myeloid cells.

Stacked bar chart analysis (**Figure 5B**) showed *K. pintolopesii*-treated mice had elevated relative abundance of macrophages and non-classical monocytes, with depleted classical monocytes (relative to controls); cDC1, cDC2, and mDC proportions remained unaltered. Pseudotime trajectory analysis (**Figure 5D**) confirmed preserved core myeloid developmental lineages in both groups, with no major trajectory disruption. Violin plots (**Figure 5E**) showed cells retained core transcriptional identities across conditions, indicating treatment modulates subset frequencies rather than driving lineage trans-differentiation.

Differential expression analysis (**Figure 5F**, p < 0.05) identified widespread transcriptional dysregulation: upregulated genes included heat shock proteins (Hspalb, Hspala, markers of cellular stress), Igkc (immunoglobulin-related immune regulation), and Vcan (extracellular matrix remodeling); key myeloid effector genes (C1qc, Lyz2, Fabp5) were downregulated, indicating attenuated pro-inflammatory and phagocytic activity. KEGG pathway enrichment analysis (**Figure 5G**) identified enrichment of ferroptosis-related pathways as the most significantly enriched, along with mineral absorption, Chagas disease, and pertussis-related pathways. Wikipathway analysis (**Figure 5H**) confirmed enrichment of oxidative damage response, classical complement activation, microglial pathogen phagocytosis, and iron homeostasis pathways, indicating *K. pintolopesii* induces robust oxidative stress and disrupts iron metabolic homeostasis in splenic myeloid cells.

Focusing on monocytes, pseudotime trajectory analysis (**Figure 5D**) showed classical monocytes occupied a trajectory branch markedly reduced in *K. pintolopesii*-treated samples (consistent with depleted proportions). Non-classical monocytes expanded along a separate branch, with sustained expression of patrolling-associated markers (*Itgal*, *Ace*). Classical monocytes showed reduced pro-inflammatory gene expression (*Ly6c2*, *Chil3*), while both subsets contributed to oxidative stress and ferroptosis pathway activation.

To further dissect the underlying regulatory and cellular communication changes, we analyzed transcription factor (TF) regulon activity and intercellular signaling networks (**Supplementary Figure 3**). Regulon specificity scoring (**Supplementary Figure 3A**) revealed enhanced activity of stress- and iron metabolism-associated TFs (e.g., *Egr1*, *Mafb*, *Bola1*) in the treated group, alongside diminished activity of pro-inflammatory TFs (e.g., *Zbtb7a*), aligning with the observed attenuation of myeloid effector function. Cell-cell interaction networks (**Supplementary Figures 3C–G**) demonstrated that *K. pintolopesii* treatment reshaped myeloid communication: interactions between classical monocytes and other splenic myeloid subsets were weakened, while non-classical monocytes and macrophages established more frequent connections, particularly via pathways linked to oxidative stress and complement activation. Ligand-receptor interaction heatmaps (**Supplementary Figures 3E, F**) further validated this shift, showing enhanced signaling in classical complement activation (e.g., *C1qb*-*Cd14*) and oxidative stress response (e.g., *Tnf*-*Tnfrsf1a*) pathways, while pro-inflammatory ligand-receptor pairs (e.g., *IL-1b*-related complexes) were suppressed.

This monocyte compartment remodeling, depletion of pro-inflammatory classical monocytes and expansion of stress-adapted non-classical monocytes, likely reshapes the splenic immune microenvironment by modulating inflammatory tone and cellular clearance capacity, potentially exacerbating age-related splenic dysfunction. The concurrent enrichment of ferroptosis-related pathways and iron homeostasis disruption, coupled with rewired intercellular communication and TF regulon activity, underscores a multi-layered mechanism by which *K. pintolopesii* drives splenic myeloid aging, linking microbial perturbation to accelerated immune senescence.

### 3.6 *K. pintolopesii* intervention induces B cell immuno-senescence in the aging spleen

*K. pintolopesii* intervention profoundly reshaped B cell subset composition in the aging mouse spleen. UMAP visualization (**Figure 6A**) resolved seven distinct B cell subsets: marginal zone B cells (MZBCells_Cr2), two mature B cell subsets (MatureB_Aff3, MatureB_Tex14), memory B cells (MemoryB_Ighg2b), and three naive B cell subsets (NaiveB_Ccr1, NaiveB_Pmna, NaiveB_Vpreb3). Quantitative subset analysis (**Figure 6B**) revealed a dramatic redistribution following *K. pintolopesii* treatment: MZBCells_Cr2 frequency was markedly elevated, functionally competent mature B cell proportions were significantly reduced, MemoryB_Ighg2b showed mild expansion, while naive B cell subsets remained largely stable. Subset-specific gene expression profiling (**Figures 6C, D**) validated the identity of each cluster, confirming robust clustering and annotation fidelity.

**Figure 6:**
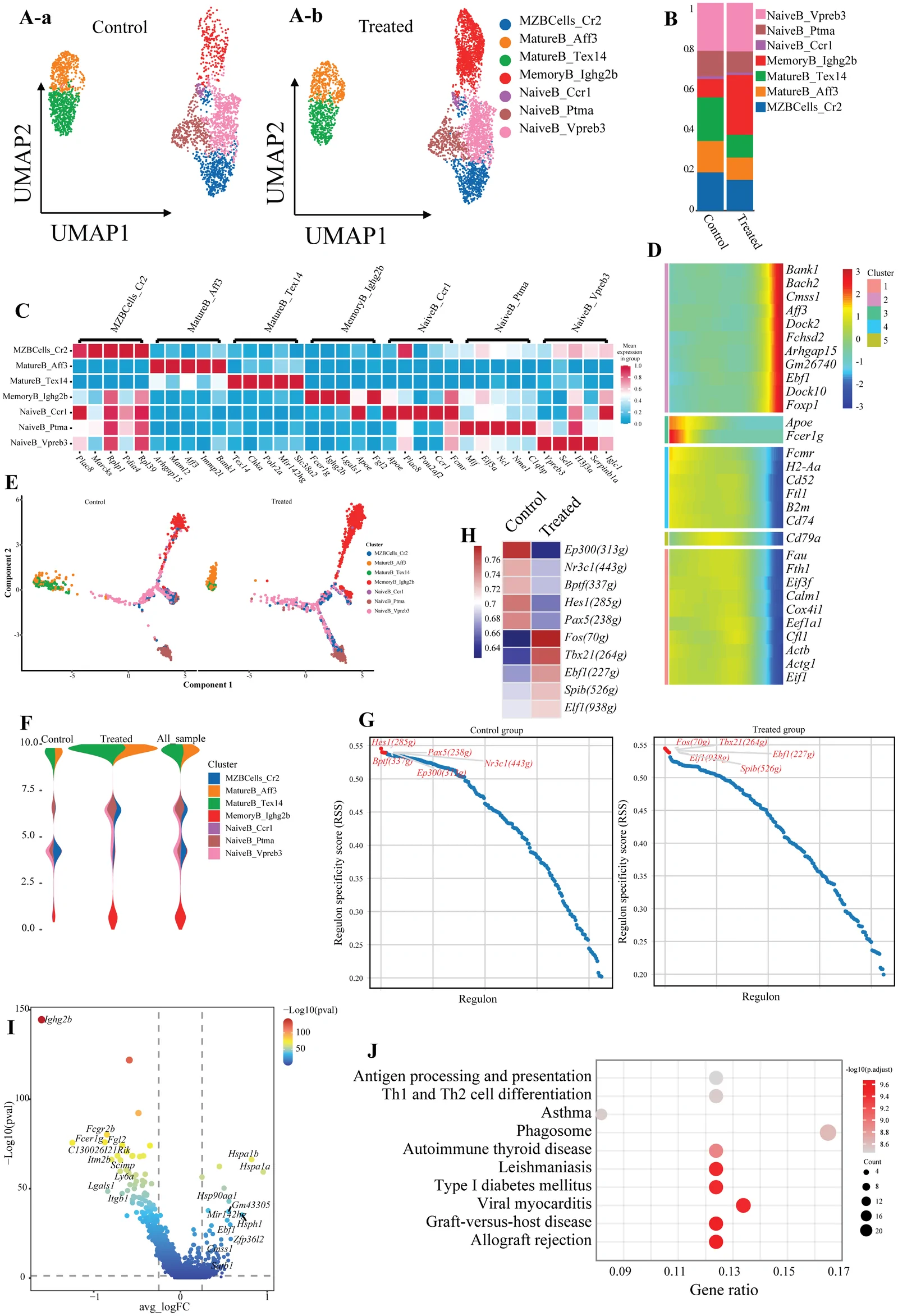
K*. pintolopesii* induces B cell immune senescence via accelerated terminal differentiation to a pro-senescent marginal zone phenotype in aged mice. **(A)** UMAP visualization of 7 distinct splenic B cell subsets identified by scRNA-seq in control and *K. pintolopesii*-treated mice. **(B)** Proportional changes in B cell subsets, showing increased marginal zone B cells (MZBCells_Cr2) and depleted mature B cells in the treated group. **(C, D)** Co-expression module analysis of B cell genes, identifying three modules associated with senescence/inflammation (Module 1), metabolic dysregulation (Module 2), and B cell receptor signaling (Module 3); *K. pintolopesii* activates Module 1 and suppresses Module 3. **(E)** Pseudotime trajectory analysis of B cells, showing enrichment of treated B cells at the MZBCells_Cr2 terminal branch. **(F)** Distribution of B cell subsets along the pseudotime trajectory. **(G)** Region-specific score (RSS) analysis, confirming enhanced MZBCell-associated TF enrichment at the trajectory terminus in the treated group. **(H)** Differential TF expression heatmap, showing downregulation of B cell maturation TFs and upregulation of senescence/activation TFs in the treated group. **(I)** Volcano plot of DEGs in B cells between control and *K. pintolopesii*-treated groups (|log_2_FC| ≥1, adjusted *p* < 0.05). **(J)** KEGG pathway enrichment analysis of DEGs, linking *K. pintolopesii*-induced B cell changes to senescence-related inflammatory diseases.

Pseudotime trajectory analysis (**Figures 6E, F**) reconstructed a continuous developmental trajectory originating from naive B cells, progressing through memory and mature B cell compartments, and culminating in MZBCells_Cr2 and MatureB_Tex14 as terminal states. *K. pintolopesii*-treated B cells were predominantly enriched at the MZBCells_Cr2 terminal branch, whereas control B cells localized to intermediate naive/mature compartments. Region-specific score (RSS) analysis (**Figure 6G**) confirmed that *K. pintolopesii* enhanced the enrichment of MZBCell-associated transcription factors (TFs) at the trajectory terminus, while suppressing the accumulation of mature B cell-related TFs in intermediate segments. These findings indicate accelerated B cell terminal differentiation toward a pro-senescence, exhausted-like marginal zone phenotype.

Differential TF expression analysis (**Figure 6H**, *p* < 0.05) demonstrated that control B cells highly expressed TFs critical for B cell lineage maintenance and maturation (e.g., *Pax5*, *Ep300*, *Nrs1*), while *K. pintolopesii*-treated B cells upregulated TFs linked to B cell activation, marginal zone differentiation, and senescence-related stress (e.g., *Spdb*, *Elf1*, *Thx2l*). Co-expression module analysis (**Figures 6C, D**) identified three gene clusters: Module 1 (e.g., *Bank1*, *Bach2*, *Atf3*) associated with pro-senescence and inflammation; Module 2 (e.g., *Apoe*, *Fcrlg*) involved in age-related metabolic dysregulation; and Module 3 (e.g., *Cd79a*, *Cd74*) linked to functional B cell receptor signaling. *K. pintolopesii* strongly activated Module 1 and suppressed Module 3, indicating a transcriptomic switch from a youthful, functional B cell program to a senescence-associated pro-inflammatory program.

Differential gene expression analysis (**Figure 6I**, *p* < 0.05) corroborated these subset shifts: *K. pintolopesii*-treated B cells upregulated memory/activated B cell markers (e.g., *Ighg2b*, *Fcrla*) and downregulated core functional B cell signature genes (e.g., *Cd19*, *Cd79b*). Pathway enrichment analysis (**Figure 6J**) showed these differentially expressed genes (DEGs) were significantly enriched in senescence-related inflammatory diseases (e.g., autoimmune thyroid disease, asthma, graft-versus-host disease), establishing a direct link between *K. pintolopesii*-induced B cell alterations and pathological inflammation, a hallmark of immune-senescence. WikiPathway analysis further revealed activation of Type II interferon signaling, marked by decreased expression of *Cybb* and *Psmb9*, alongside increased expression of *Irf8*, *Socs1*, and *Stat1* in *K. pintolopesii*-treated B cells.

Cell–cell communication analysis (**Supplementary Figure 4A**) revealed that *K. pintolopesii* intervention remodeled the B cell-centric splenic interaction network: treated B cells exhibited enhanced interactions with T cells, neutrophils, and plasma cells, while interactions with erythrocytes and monocytes were diminished relative to controls. Ligand–receptor (L–R) pair analysis (**Supplementary Figures 4B, C**) further demonstrated that control B cells predominantly expressed ligands/receptors associated with homeostatic B cell maintenance and immune quiescence (e.g., *LTA*/*TNFRSF1B*, *TGFB1*/*TGFBR*, *PTPRC*/*CD22*), whereas *K. pintolopesii*-treated B cells upregulated pro-inflammatory and senescence-associated L–R pairs (e.g., *ProstaglandinE2*/*PTGER*, *integrin αIIbβ3 complex*/*ICAM1*, *CD48*/*CD244*), alongside activation-related ligands (e.g., *APP*/*CD74*, *APP*/*SORL1*). Quantitative interaction counts analysis (**Supplementary Figure 4D**) confirmed that *K. pintolopesii* increased the total number of B cell interactions, particularly strengthening connections between MZBCells_Cr2 and MemoryB_Ighg2b, while reducing interactions between naive B cell subsets (NaiveB_Ccr1, NaiveB_Pmna, NaiveB_Vpreb3) and mature B cell subsets (MatureB_Aff3, MatureB_Tex14). These L–R changes indicate that *K. pintolopesii* rewires the splenic immune microenvironment to promote pro-senescence, pro-inflammatory signaling cascades that reinforce B cell terminal differentiation and immune exhaustion.

Synthesizing these results position *K. pintolopesii* as a potent driver of B cell immune-senescence, orchestrating a coordinated shift in subset identity, transcriptomic programming, and intercellular signaling that undermines adaptive immune competence while amplifying inflammatory pathology. This work provides a mechanistic framework for understanding how microbial interventions modulate age-related immune decline, and highlights the splenic B cell compartment as a critical target for interventions aimed at mitigating senescence-associated inflammation.

### 3.7 Core mechanisms of *K. pintolopesii*-induced splenic senescence integrating the ferro-aging concept

A recent Cell Metabolism study proposed ferro-aging, a programmed aging pathway triggered by iron overload, driven by ACSL4, and mediated by lipid peroxidation (34). This framework provides a novel perspective for interpreting iron metabolism-related organ aging, and ferroptosis, a regulated cell death closely linked to ferro-aging, may also participate in this process. Our snRNA-seq, scRNA-seq, and histopathological findings suggest that *K. pintolopesii* is associated with aggravated splenic aging, potentially mediated by ferro-aging activation and/or ferroptosis. This is supported by dysregulated splenic structure, immune cell subsets (T cells, B cells, myeloid cells), and molecular pathways (ferroptosis, oxidative stress, antigen presentation), reflecting systemic remodeling of the splenic immune microenvironment where ferro-aging and ferroptosis may act as key bridging mechanisms. The core mechanisms are as follows:

Notably, our previously published study (29) further confirms *K. pintolopesii*’s regulatory role in immune cell fatty acid metabolism, ferroptosis, and ferro-aging using BALB/c ZAP70^W163C^ mutant mice. Consistent with the aforementioned published findings, *K. pintolopesii* intervention in these mice disrupts fatty acid metabolism in splenic T cells, B cells, and myeloid cells, accompanied by ferroptosis induction and ferro-aging progression. Specifically, *K. pintolopesii* imbalances fatty acid synthesis and oxidation, downregulates fatty acid metabolism-related genes (e.g., *Fabp5*, *Apoe*), and upregulates the key ferro-aging/ferroptosis driver ACSL4, promoting lipid peroxidation, a common core process of both pathways. *K. pintolopesii*-induced iron overload further exacerbates these dysregulations, forming an amplification loop among iron homeostasis disorder, fatty acid metabolism disorder, ferroptosis, and ferro-aging, ultimately accelerating splenic immune senescence (29). These results verify the universality of *K. pintolopesii*’s regulatory role in splenic immune cell fatty acid metabolism, ferroptosis, and ferro-aging.

#### 3.7.1 Structural and pathological deterioration: premature aging of splenic parenchyma

H&E, Masson’s trichrome, and Prussian blue staining (**Figures 1E–G**) confirm that *K. pintolopesii*-induced histopathological alterations directly reflect accelerated splenic aging, consistent with ferro-aging characteristics and potentially linked to ferroptosis. White pulp lymphocyte rarefaction indicates reduced splenic immune reserve, a typical aging feature, while the overall splenic architecture remains intact. Mild interstitial collagen hyperplasia signals early splenic fibrosis, with *K. pintolopesii* modestly increasing collagen deposition, particularly in the red pulp and white pulp marginal zones, which is associated with cellular senescence and chronic inflammation. Notably, prominent iron overload (evidenced by Prussian blue staining), a key ferro-aging initiator, may trigger ferroptosis: excess iron induces reactive oxygen species (ROS) via the Fenton reaction, promoting lipid peroxidation, a core mediator of both ferro-aging and ferroptosis, leading to oxidative stress, DNA damage, cellular senescence, and/or ferroptotic cell death. Collectively, these processes exacerbate splenic structural and functional decline.

To further validate the activation of ferroptosis and inflammation in *K. pintolopesii*-treated spleens, immunohistochemistry (IHC) was performed to detect key protein markers, with integrated optical density (IOD) values calculated for semi-quantitative analysis (**Figures 7A, B**). Consistent with ferroptosis activation, the expression of ACSL4, a critical driver of lipid peroxidation and ferroptosis, was significantly elevated in the treated group compared to controls. Concurrently, the iron metabolism regulator FTH1, which sequesters excess free iron to mitigate ferroptosis risk, showed increased expression, likely reflecting a compensatory response to iron overload. Meanwhile, pro-inflammatory markers including IL-1β, IL-17, NF-κB p65, and TNF-α were also upregulated, indicating robust activation of inflammatory signaling cascades in the splenic parenchyma following *K. pintolopesii* challenge. These IHC results, supported by IOD quantification (**Figure 7B**, *p* < 0.05), provide direct evidence linking ferroptosis-related molecular events (iron dyshomeostasis, ACSL4 upregulation) with heightened inflammation, which collectively contribute to splenic premature aging.

**Figure 7:**
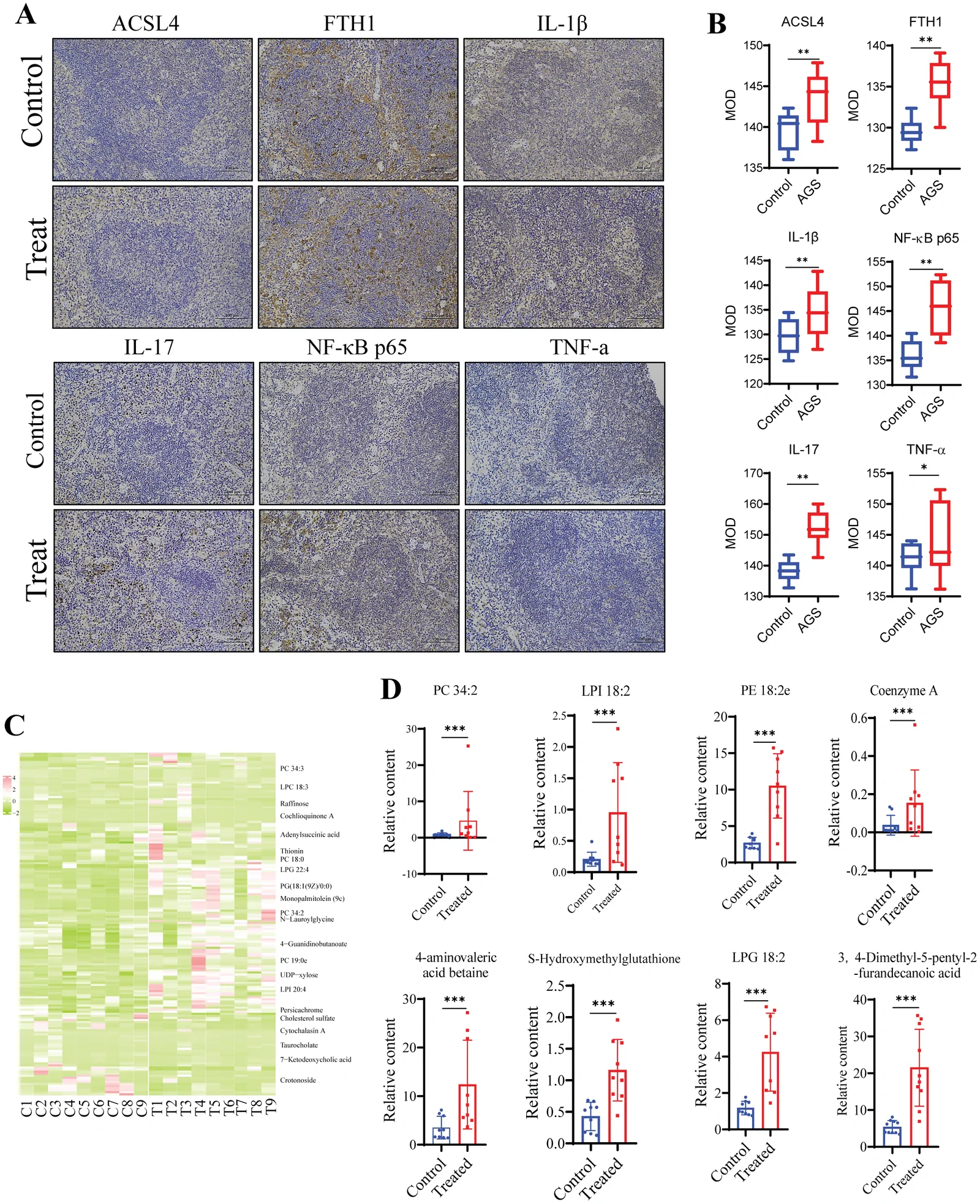
K*. pintolopesii* triggers ferroptosis and inflammatory responses in aged mouse spleens while altering the metabolomic landscape. **(A)** Representative immunohistochemical (IHC) staining of key markers related to ferroptosis (ACSL4, FTH1) and inflammation (IL-1β, IL-17, NF-κB p65, TNF-α) in spleen tissues from control and **(K)** pintolopesii-treated aged mice. Scale bars are included in the original images. **(B)** Quantification of the mean optical density (MOD) of the IHC staining in **(A)**, showing significantly increased expression of all tested markers in the treated group compared to controls. Data are presented as box-and-whisker plots, with n = 6 mice per group. Statistical significance was determined by Student’s t-test (*p < 0.05, **p < 0.01). **(C)** Heatmap visualization of differentially abundant metabolites in the spleen of control versus **(K)** pintolopesii-treated aged mice. Color intensity represents relative metabolite levels (green: downregulated; red: upregulated). **(D)** Box plots showing the relative content of representative significantly altered metabolites in the treated versus control groups, including lipids (PC 34:2, LPI 18:2, PE 18:2e, LPG 18:2), coenzyme A, and other metabolic intermediates (4-aminovaleric acid betaine, S-hydroxymethylglutathione, 3,4-Dimethyl-5-pentyl-2-furandecanoic acid). Data are presented as mean ± SD, with n = 6 per group. ***p < 0.001 by Student’s t-test.

Metabolomic profiling of 546 metabolites in splenic tissues from *K. pintolopesii*-treated mice identified 61 differential metabolites (35 upregulated, 26 downregulated; **Figure 7C**; *p* < 0.05; **Supplementary Table 1**), with robust associations with lipid metabolism, oxidative stress, and ferroptosis pathways. In lipid metabolism, 26 differential metabolites included significantly upregulated glycerophospholipids (PC 34:2, log2FC=2.34; LPI 18:2, log2FC=2.05, **Figures 7C, D**, *p* < 0.05) and polyunsaturated fatty acids (conjugated linoleic acid, log2FC=1.94). Combined with elevated levels of coenzyme A (**Figures 7C, D**, *p* < 0.05), a key mediator of lipid synthesis, these changes directly support ACSL4-driven lipid metabolic reprogramming and lipid peroxidation activation. For oxidative stress, 5 differential metabolites featured upregulated glutathione system molecules (e.g., S-hydroxymethylglutathione, **Figures 7C, D**, *p* < 0.05), reflecting a compensatory cellular response to lipid peroxidation-induced damage. The 8 ferroptosis-related differential metabolites were characterized by the accumulation of polyunsaturated fatty acids (conjugated linoleic acid, arachidonic acid), which, together with splenic iron overload confirmed by Prussian blue staining, form a complete evidence chain that iron overload to lipid peroxidation and then ferroptosis. These metabolic alterations are highly consistent with single-cell sequencing findings, including ACSL4 upregulation, activation of iron metabolism-related transcription factors (*Egr1*, *Mafb*), and downregulation of pro-inflammatory transcription factors (*Zbtb7a*). Collectively, they validate at the metabolic level the molecular mechanism by which *K. pintolopesii* regulates myeloid function and accelerates premature splenic aging through the ACSL4-lipid peroxidation-ferroptosis pathway.

#### 3.7.2 Immune cell subset remodeling: immune homeostasis imbalance and accelerated cellular senescence

*K. pintolopesii* -induced splenic aging is characterized by comprehensive splenic immune cell subset remodeling, with B cells, T cells, and myeloid cells showing senescence-related phenotypic shifts that disrupt immune homeostasis, validated by scRNA-seq, UMAP, and pseudotime trajectory analyses.

For T cells, *K. pintolopesii* profoundly remodels T cell homeostasis, accelerating naive T cell (CD4NaiveT_Ly6c1, CD8NaiveT_Sell) differentiation into effector/memory subsets (CD4^+^ memory T cells, CD8^+^ effector T cells, Tregs, NKT cells). While this enhances activation, it causes T cell exhaustion and senescence by depleting the naive T cell pool. Sustained activation, mediated by upregulated activation TFs (*Egr1*, *Jun*, *Fos*, *Atf3*) and immune pathways (Th17, NF-κB, IL-17, TCR signaling), triggers chronic inflammation, further accelerating senescence. *K. pintolopesii* also reshapes T cell intercellular communication, enhancing effector/memory T cell interactions and upregulating pro-inflammatory signaling (*Tnf-Tnfrsf1b*, *Ifng-Ifngr1*) and immune checkpoint molecules (*Pdcd1-Cd274*, *Ctla4-Cd80/CD86*), balancing activation and homeostasis but contributing to senescence.

Bulk RNA-seq profiling of splenic T cells *in vitro* (induced by lysate of *K. pintolopesii*) corroborated these single-cell findings at the population level. Pathway enrichment analysis revealed that *K. pintolopesii* treatment significantly upregulated pathways linked to oxidative phosphorylation, neurodegenerative disease, ribosome biogenesis, apoptosis, and p53 signaling, while downregulating viral infection response, JAK-STAT signaling, and ubiquitin-mediated proteolysis (**Figure 8B**). KEGG analysis of downregulated DEGs further highlighted impaired cellular homeostasis, with suppressed processes including apoptosis, adherens junction assembly, endocytosis, RNA processing, and DNA mismatch repair (**Figure 8C**). Expression heatmaps (**Figure 8A**, *p* < 0.05) confirmed elevated levels of mitochondrial oxidative phosphorylation genes (e.g., *mt-Co2*, *mt-Atp6*, *mt-Nd1/2*) and key T cell activation transcription factors (e.g., *Atf3*, *Egr1*) in treated samples. Gene set enrichment analysis (GSEA) validated robust enrichment of the oxidative phosphorylation pathway (**Figure 8D**, NES = 1.97, *p* = 0.001499), indicating a metabolic shift toward mitochondrial respiration to sustain prolonged T cell activation. Together, these bulk transcriptomic changes align with our single-cell data, confirming that *K. pintolopesii* drives T cell activation and metabolic reprogramming while promoting exhaustion and senescence via dysregulated energy metabolism and compromised cellular repair.

**Figure 8:**
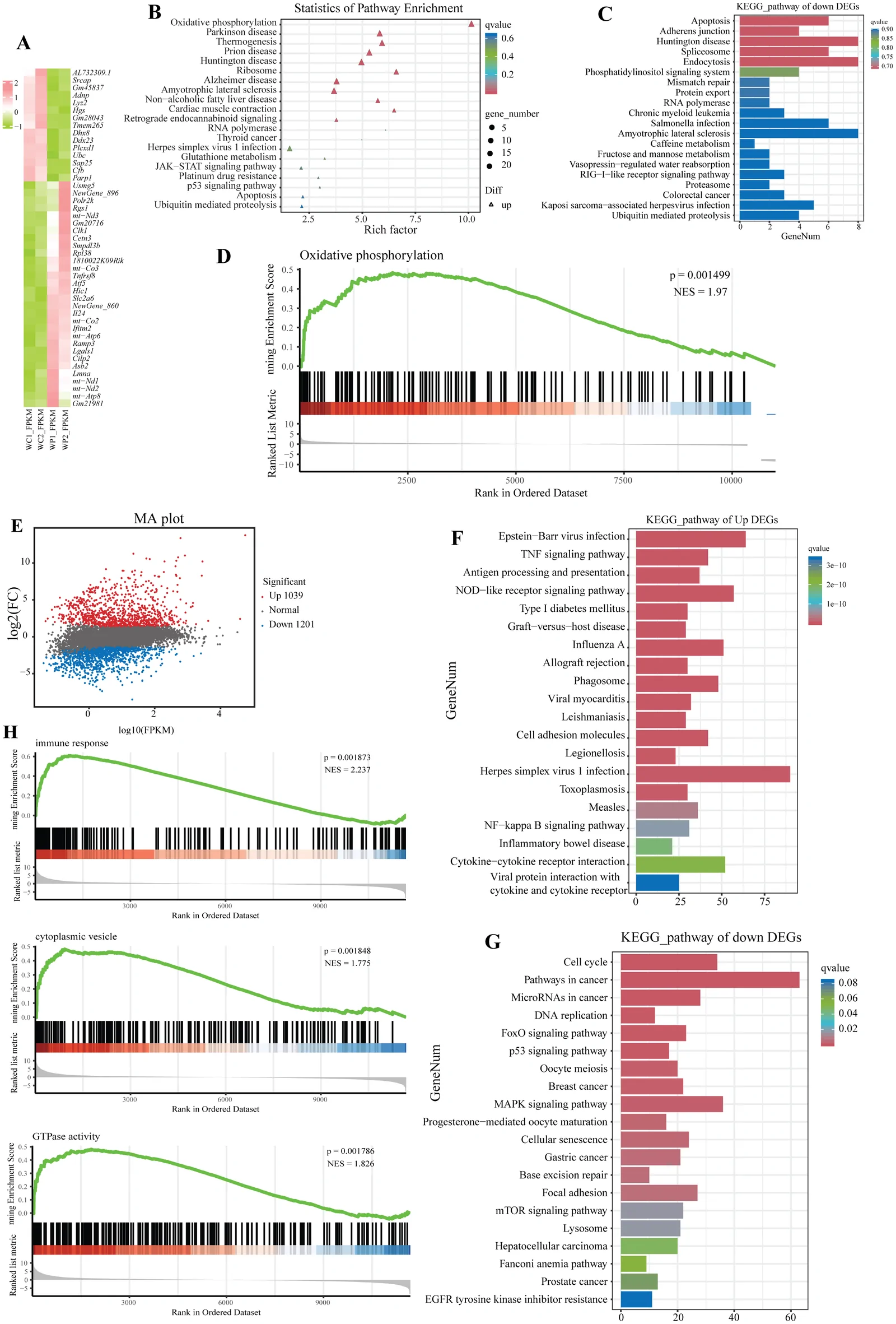
Bulk RNA-seq reveals transcriptomic reprogramming of splenic T cells and bone marrow-derived macrophages (BMDMs) upon *K. pintolopesii* stimulation. **(A)** Heatmap of DEGs in splenic T cells from control and *K. pintolopesii*-treated groups, showing upregulation of mitochondrial oxidative phosphorylation and activation-related genes. **(B)** KEGG pathway enrichment analysis of upregulated DEGs in T cells, highlighting oxidative phosphorylation and apoptosis pathways. **(C)** KEGG pathway enrichment analysis of downregulated DEGs in T cells, showing impaired cellular homeostasis and repair pathways. **(D)** GSEA analysis validating enrichment of the oxidative phosphorylation pathway in *K. pintolopesii*-treated T cells (NES = 1.97, *p* = 0.001499). **(E)** Volcano plot of DEGs in BMDMs between control and *K. pintolopesii*-treated groups (FDR < 0.01, *p* < 0.05), with 1039 upregulated and 1201 downregulated genes. **(F)** KEGG pathway enrichment analysis of upregulated DEGs in BMDMs, highlighting pro-inflammatory and anti-infective signaling pathways. **(G)** KEGG pathway enrichment analysis of downregulated DEGs in BMDMs, showing suppressed cell cycle and proliferation pathways. **(H)** GSEA analysis revealing enrichment of immune response, cytoplasmic vesicle trafficking, and GTPase activity pathways in treated BMDMs.

Integrated UMAP visualization and CytoTRACE-based differentiation potential analysis revealed a continuous T cell differentiation trajectory, with distinct populations occupying defined positions along the axis. CytoTRACE entropy analysis (**Figures 3A, B**) demonstrated that naive T cells exhibited the highest differentiation potential (lowest entropy values), while terminally differentiated effector and regulatory subsets displayed the lowest potential (highest entropy values). Notably, the CD4Tmem_S100a4 subset was characterized by significantly elevated entropy values, indicating enhanced cellular plasticity relative to terminal effector/regulatory subsets. This places it at a critical intermediate state along the differentiation trajectory. Regulon specificity score (RSS) analysis (**Figure 3C**) identified a unique transcription factor regulon signature governing the CD4Tmem_S100a4 state, with Egr1 emerging as the top-ranked and most specific regulon (**Figure 3D**), alongside key co-regulators including *Fli1*, *Klf2*, and *Mecp2*. Subsequent transcriptomic profiling (**Figure 3G**) further revealed that CD4Tmem_S100a4 co-expressed memory-associated genes (S100a4), antigen presentation machinery (*H2-Aa*, *H2-Eb1*, *Cd74*), and activation markers, while maintaining low expression of terminal effector genes such as *Gzmk* and *Gzmb*. Functional enrichment analysis using WikiPathways, Reactome, and KEGG databases (**Figures 3H–J**) demonstrated that CD4Tmem_S100a4 was centrally involved in key pathways including TCR signaling, NF-κB activation, cytokine signaling, PD-1 checkpoint signaling, and apoptosis, reflecting its dual capacity for activation and immunomodulation. Notably, in the treated group, both the abundance of CD4Tmem_S100a4 cells and the expression levels of its signature markers, CD4, S100A4, and the master regulator EGR1, were significantly downregulated (**Figures 3D, E**; *p* < 0.01), as validated by immunofluorescence and quantitative analysis. Functional enrichment further highlighted suppressed MAPK signaling, cytokine responses, and T cell activation pathways in the treated state. Collectively, these findings identify CD4Tmem_S100a4 as the core transitional subset and pivotal regulatory node in T cell fate determination, highlighting its critical role as a target for therapeutic immunomodulation.

For myeloid cells, *K. pintolopesii* reshapes the composition of splenic myeloid subsets while preserving lineage integrity: it increases the proportions of macrophages and non-classical monocytes, depletes classical monocytes, and maintains stable frequencies of conventional dendritic cells (cDC1, cDC2) and myeloid dendritic cells (mDC). Pseudotime trajectory analysis confirms that these changes reflect modulated subset frequencies rather than induced trans-differentiation, ruling out lineage reprogramming. Functionally, splenic myeloid cells exhibit attenuated pro-inflammatory and phagocytic capacities, as evidenced by downregulated expression of key functional genes (*C1qc*, *Lyz2*, *Fabp5*). Conversely, stress-responsive heat shock proteins (*Hspa1b*, *Hspa1a*) are upregulated, indicating a shift toward stress adaptation. KEGG pathway enrichment analysis identifies ferroptosis as the top enriched pathway, alongside mineral absorption and iron homeostasis, suggesting ferroptosis contributes to myeloid senescence and potential crosstalk with ferroptosis-associated aging. Both pathways, tightly regulated by ACSL4-driven lipid peroxidation, may collectively impair myeloid function, reduce debris/pathogen clearance, and promote senescent cell accumulation. Transcriptionally, this functional attenuation aligns with enhanced expression of stress/iron metabolism-related transcription factors (*Egr1*, *Mafb*, *Bola1*) and diminished levels of pro-inflammatory transcription factor *Zbtb7a*.

Transcriptomic profiling of bone marrow-derived macrophages (BMDMs) treated with *K. pintolopesii* versus untreated controls uncovered extensive, functionally coordinated remodeling of the myeloid cell transcriptome (**Figures 8E–H**). A total of 2,240 differentially expressed genes (DEGs) were identified, with 1,039 genes significantly upregulated and 1,201 genes downregulated (FDR < 0.01, *p* < 0.05; **Figure 8E**; **Supplementary Table 2**), reflecting profound shifts in the molecular pathways governing macrophage activation and homeostasis. Gene set enrichment analysis (GSEA) delineated three core, interconnected regulatory axes: first, the immune response pathway was robustly activated (NES = 2.237, *p* = 0.001873), establishing a dominant pro-inflammatory program; second, cytoplasmic vesicle trafficking (NES = 1.775, *p* = 0.001848) and GTPase activity (NES = 1.826, *p* = 0.001786) pathways were significantly upregulated (**Figure 8H**), which synergistically support enhanced intracellular transport, cytokine secretion, and signal transduction that key effector processes for macrophage-mediated immunity.

KEGG pathway enrichment further refined these functional trends: upregulated DEGs converged on pro-inflammatory and anti-infective signaling networks, including *Epstein-Barr virus infection*, TNF signaling pathway, antigen processing and presentation, NOD-like receptor signaling, and inflammatory bowel disease-related pathways (**Figure 8F**). These pathways collectively drive M1 macrophage polarization, as evidenced by the marked upregulation of core pro-inflammatory mediators: pro-inflammatory cytokines (*Il6*, log_2_FC≈13.58; *Csf3*, log_2_FC=14.13), chemotactic chemokines (*Ccl5*, log_2_FC=10.35), acute-phase response proteins (*Saa3*, log_2_FC=13.39), complement system components (*Cfb*, log_2_FC=10.40), and antiviral defense genes (*Ifitm3*, log_2_FC=3.74; *Bst2*, log_2_FC=3.98). Concurrent upregulation of antigen presentation-related genes (e.g., *H2-Ab1*, *Tapbp*) further validated the enhancement of immune effector function in *K. pintolopesii*-treated BMDMs. In striking contrast, downregulated DEGs were strongly enriched in pathways governing cell growth and proliferation, including cell cycle progression, DNA replication, p53 signaling, and mTOR pathway (**Figure 8G**). This was accompanied by reduced expression of critical regulators of cell proliferation and signal transduction, such as the transcription factor *Fosb* (log_2_FC=-12.27), cytokine receptor *Lifr* (log_2_FC=-10.41), and cyclin *Ccnd1* (log_2_FC=-5.66), which reflecting a coordinated suppression of cell growth to prevent excessive immune activation and preserve cellular homeostasis. Metabolic reprogramming further supported the pro-inflammatory phenotype, with upregulation of lipid metabolism enzymes (e.g., *Acsl1*, log_2_FC=4.40; *Acsl4*, log_2_FC=0.84) that prioritize energy production to meet the heightened metabolic demands of immune effector functions.

All these data demonstrate that *K. pintolopesii* elicits a robust pro-inflammatory activation with compensatory homeostatic restraint in BMDMs: it drives robust pro-inflammatory activation, enhances antimicrobial defense via the coordinated upregulation of immune signaling and vesicle transport, and reshapes metabolism to support effector function, while the concurrent suppression of cell proliferation reflects pathological growth arrest rather than beneficial immune homeostasis maintenance. Notably, this regulatory pattern is consistent with the transcriptional changes observed in splenic myeloid cells *in vivo*, highlighting a conserved mechanism by which *K. pintolopesii* modulates myeloid cell function across physiological contexts.

For B cells, *K. pintolopesii* induces a pro-senescence shift in splenic B cells, with increased marginal zone B cells (MZBCells_Cr2, a senescence-associated subset), mild expansion of memory B cells (MemoryB_Ighg2b), and depletion of functionally competent mature B cells (MatureB_Aff3, MatureB_Tex14), impairing humoral immunity. Pseudotime analysis confirms accelerated B cell differentiation toward an exhausted marginal zone phenotype. Transcriptionally, B cells downregulate lineage maintenance/maturation TFs (*Pax5*, *Ep300*, *Nrs1*) and upregulate activation, marginal zone differentiation, and senescence-related TFs (*Spdb*, *Elf1*, *Thx2l*), activating pro-senescence/inflammatory gene modules (e.g., *Bank1*, *Bach2*, *Atf3*) and suppressing B cell receptor signaling genes (e.g., *Cd79a*, *Cd74*), locking B cells in a senescent, pro-inflammatory state.

Virtual knockout analysis of the senescence-associated transcription factors (TFs) *Atf3*, *Bach2*, and *Elf1* further confirms their regulatory roles in driving B cell dysfunction. *Atf3* knockout (**Figures 9A–a, b**) triggers robust upregulation of cytoskeletal remodeling (*Tmsb4x*), ribosomal biogenesis (*Rpl4*, *Pohr2a*), and mitochondrial respiratory chain genes (*mt-Co1*, *mt-Nd1*), consistent with its role as a stress-responsive repressor that normally restrains pro-inflammatory and metabolic activation. These upregulated genes after Atf3 virtual knockout are involved in transcription/translation, mitochondrial metabolism and oxidative stress response (49–51), which suggested that Atf3 may act as a transcriptional repressor; its deficiency triggers a compensatory cascade to maintain cellular homeostasis through coordinated regulation of multiple pathways. *Bach2* knockout (**Figures 9B–a, b**; induces dramatic overexpression of neuro-immune metabolic regulators (*Adrb2*, *Hlf2*) and spliceosomal components (*Lsm5*), reinforcing its function as a key suppressor of B cell activation and marginal zone differentiation. And *Elf1* knockout (**Figure 9C–a, b**) drives the strongest upregulation of epigenetic modifiers (*Kmt1*) and membrane trafficking genes (*Anxa6*), alongside mitochondrial genes (*mt-Co2*), aligning with its role in repressing cell cycle and senescence-associated programs.

**Figure 9:**
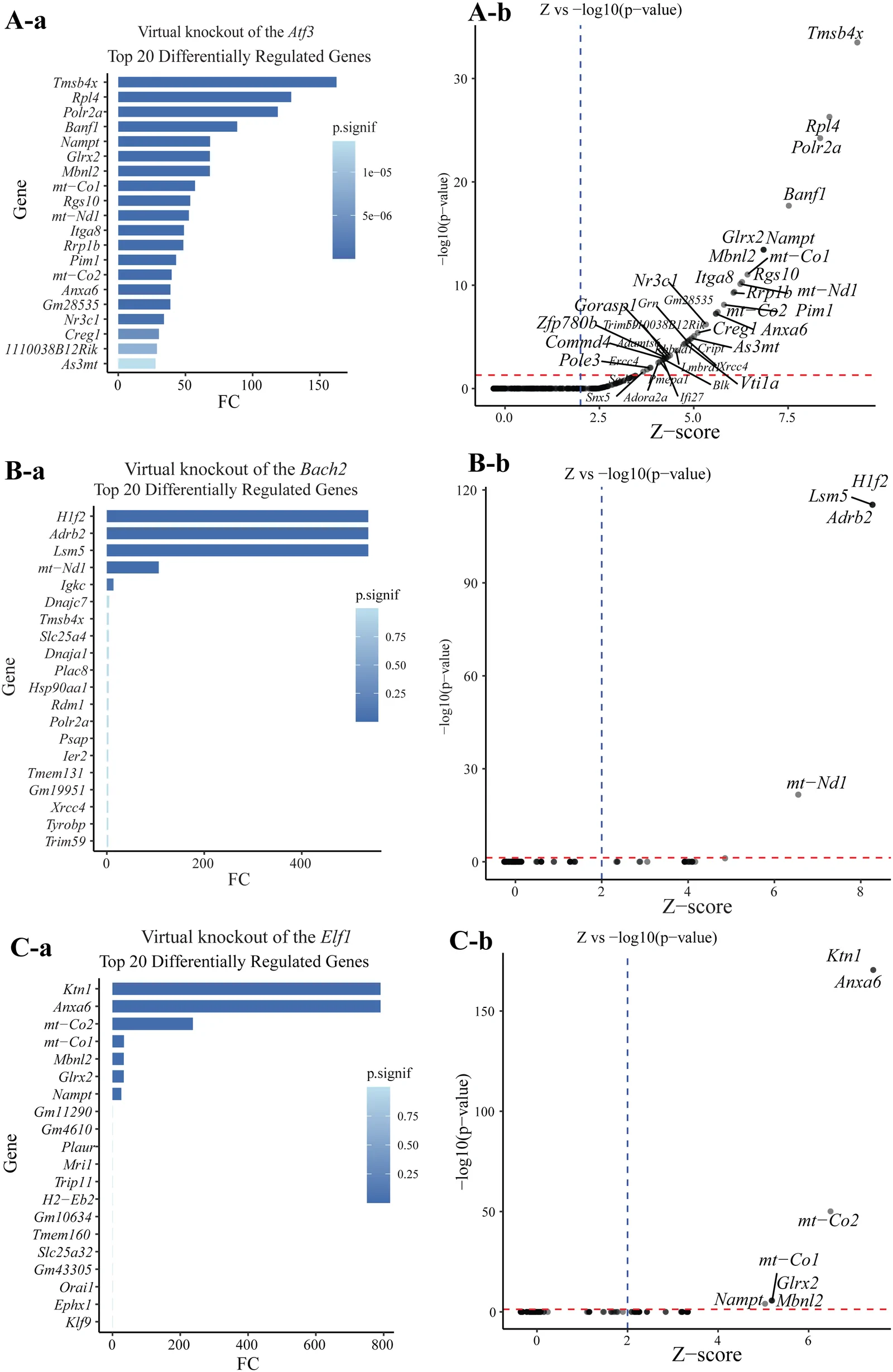
In silico virtual knockout analysis of senescence-associated transcription factors (*Atf3*, *Bach2*, *Elf1*) reveals their regulatory roles in *K. pintolopesii*-induced B cell dysfunction. **(A)** Atf3 virtual knockout: **(a)** Volcano plot of DEGs (FC ≥2 or ≤0.5, FDR < 0.05) and **(b)** KEGG pathway enrichment analysis, showing upregulation of cytoskeletal remodeling, ribosomal biogenesis, and mitochondrial respiration genes. **(B)** Bach2 virtual knockout: **(a)** Volcano plot of DEGs and **(b)** KEGG pathway enrichment analysis, revealing overexpression of neuro-immune metabolic regulators and spliceosomal components. **(C)** Elf1 virtual knockout: **(a)** Volcano plot of DEGs and **(b)** KEGG pathway enrichment analysis, showing upregulation of epigenetic modifiers, membrane trafficking genes, and mitochondrial genes. All three TFs act as transcriptional repressors, whose upregulation in *K. pintolopesii*-exposed B cells drives senescence and pro-inflammatory polarization.

Combined with UMAP clustering (**Figures 6A–C**), CytoTrace entropy analysis (**Figure 10B**), pseudotime trajectory (**Figures 10A, C**), feature gene heatmap (**Figure 6D**), and pseudotime density violin plot (**Figure 6F**), we systematically reconstructed the developmental trajectory of B cells. Under physiological conditions, naive B cells (NaiveB_Vpreb3, NaiveB_Ptma, NaiveB_Ccr1) serve as the origin of differentiation, which terminally differentiate into mature B cells (MatureB_Aff3, MatureB_Tex14) and memory B cells (MemoryB_Ighg2b) via a core transitional subset. Under physiological conditions, MZBCells_Cr2 functions as a core transitional hub with high differentiation potential, possessing dual molecular characteristics of both naive and terminally differentiated B cells. However, upon *K. pintolopesii* exposure, this subset undergoes pathological reprogramming toward a pro-senescence, pro-inflammatory state, as evidenced by the upregulation of senescence-associated transcription factors (Spib, Jun, Bhlhe40, Zfx) and activation of autoimmune-related signaling pathways. Verified by CytoTrace analysis (**Figure 10B**), MZBCells_Cr2 maintains high differentiation potential and stably connects the initial and terminal stages of B cell differentiation in both control and treated groups, fully meeting the criteria of a key transitional subset. Following intervention, the altered proportion of MZBCells_Cr2 directly drives the remodeling of terminal B cell subsets, confirming its central role in regulating B cell fate determination. Collectively, this study identifies a key target for the intervention of B cell-mediated immune disorders.

**Figure 10:**
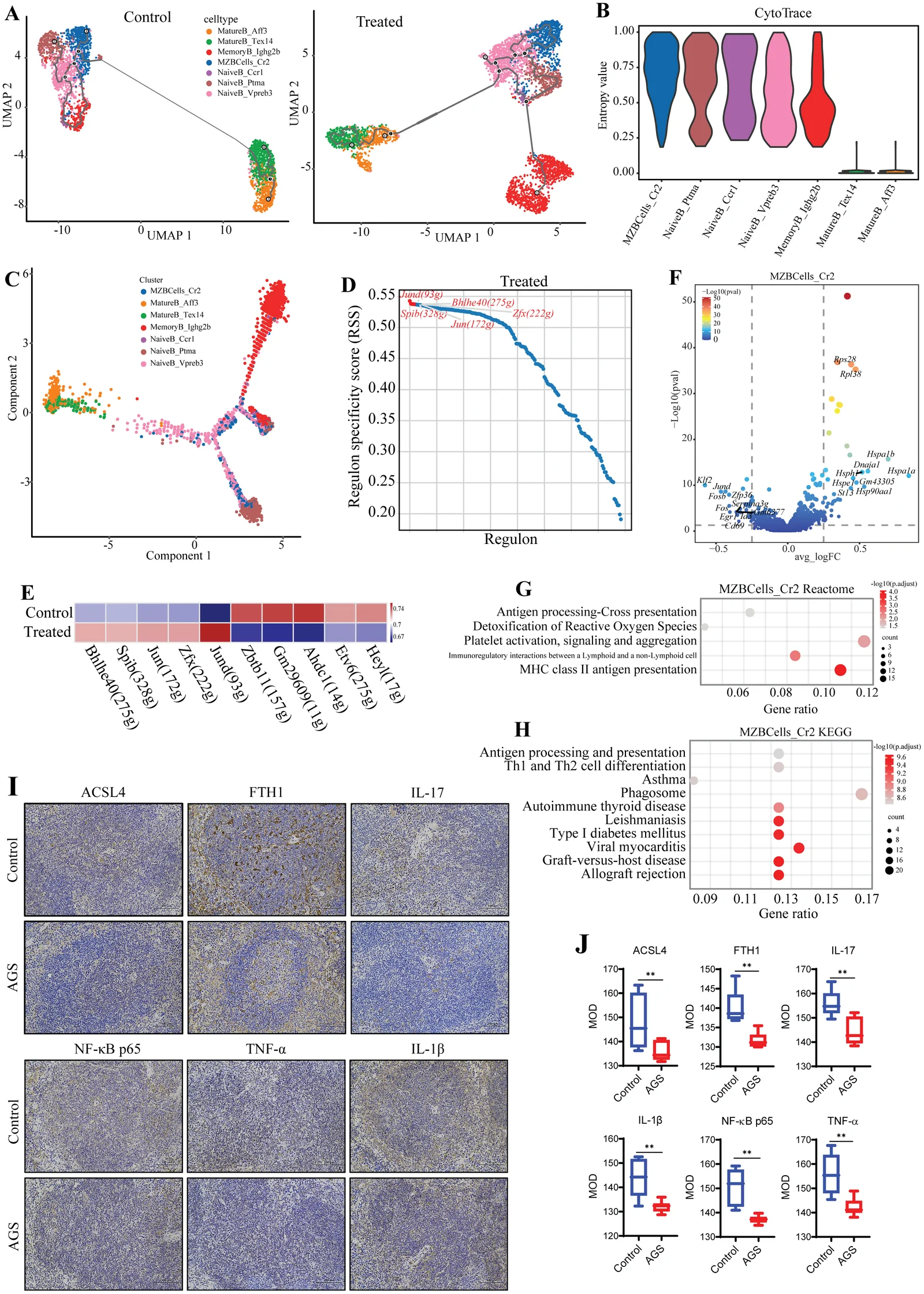
AGS treatment reverses *K. pintolopesii*-induced B cell senescence and ferroptotic/inflammatory responses in aged mice. **(A)** UMAP visualization of splenic B cell subsets in control and AGS-treated aged mice, showing partial restoration of the B cell compartment with reduced enrichment of the pro-senescent marginal zone B cell (MZBCells_Cr2) phenotype. **(B)** CytoTrace entropy analysis of B cells, demonstrating reduced cellular senescence and terminal differentiation in the AGS-treated group. **(C)** Pseudotime trajectory analysis of B cells, showing reduced enrichment of B cells at the MZBCells_Cr2 terminal branch in the AGS-treated group. **(D)** Region-specific score (RSS) analysis of transcription factors (TFs) along the pseudotime trajectory, indicating downregulation of pro-senescent TFs (e.g., *Bhlhe40*, *Zbtb11*) at the trajectory terminus following AGS treatment. **(E)** Heatmap of differential TF expression in B cells, showing restored expression of mature B cell TFs and suppressed expression of senescence/activation-associated TFs in the AGS-treated group compared to the control. **(F)** Volcano plot of differentially expressed genes (DEGs) in MZBCells_Cr2 between control and AGS-treated groups, highlighting downregulation of pro-inflammatory and senescence-related genes. **(G, H)** Reactome and KEGG pathway enrichment analyses of DEGs in MZBCells_Cr2, showing that AGS treatment reverses the activation of antigen presentation, ROS detoxification, and inflammatory disease pathways induced by *K. pintolopesii*. **(I)** Representative immunohistochemical (IHC) staining of key markers related to ferroptosis (ACSL4, FTH1) and inflammation (IL-1β, IL-17, NF-κB p65, TNF-α) in spleen tissues from control and AGS-treated aged mice. **(J)** Quantification of the mean optical density (MOD) of the IHC staining in **(I)**, showing significantly reduced expression of ferroptosis and inflammatory markers in the AGS-treated group compared to controls. Data are presented as box-and-whisker plots, with n = 6 mice per group. Statistical significance was determined by Student’s t-test (**p < 0.01).

MZBCells_Cr2, defined as a marginal zone B cell subset and the core transitional hub of B cell differentiation, exhibits high differentiation potential as validated by CytoTrace analysis. Upon *K. pintolopesii* exposure, the transcriptional regulatory network of MZBCells_Cr2 undergoes systematic reprogramming, with significant upregulation of core transcription factors (regulons) including *Spib*, *Jun*, *Bhlhe40*, and *Zfx* (**Figures 10D, E**), all of which are critical for B cell fate decision. Transcriptomic profiling revealed that MZBCells_Cr2 is remarkably activated, characterized by the upregulation of ribosomal proteins (Rps28, Rpl38), heat shock proteins (Hsp family), and the activation marker Cd69 (**Figure 10F**). Pathway enrichment analysis further demonstrated that MZBCells_Cr2 displays enhanced antigen presentation (via the MHC class II pathway), Th1/Th2 cell differentiation, and activation of autoimmune-related signaling pathways (**Figures 10G, H**), indicating a functional shift from a quiescent transitional state to an activated immunomodulatory state. Mechanistically, *K. pintolopesii* exposure specifically targets MZBCells_Cr2, the key hub of B cell differentiation, through the recognition of pathogen-associated molecular patterns (PAMPs), triggering innate immune activation, transcriptional reprogramming, and remodeling of the splenic immune microenvironment. This cascade of events ultimately drives the directional differentiation of B cells toward memory B cells, inhibits their terminal differentiation into mature B cells, and systematically reshapes the entire developmental trajectory of splenic B cells. Our findings uncover a novel mechanism by which commensal fungi regulate B cell development, thereby providing a potential therapeutic target for the intervention of fungal-associated immune diseases.

All these in silico perturbations validate that *Atf3*, *Bach2*, and *Elf1* act as transcriptional repressors whose upregulation in *K. pintolopesii*-exposed B cells directly mediates the shift toward a senescent, pro-inflammatory marginal zone phenotype, by de-repressing metabolic, inflammatory, and differentiation programs while suppressing B cell lineage maintenance and receptor signaling, ultimately impairing humoral immune competence, while these need more wet experiments to confirm.

#### 3.7.3 Molecular mechanisms: oxidative stress, iron homeostasis disorder, transcriptional reprogramming, and TCR repertoire remodeling

*K. pintolopesii*-induced splenic aging stems from three synergistic molecular dysregulations, closely linked to ferro-aging and ferroptosis (interconnected pathways potentially mediating aging together), validated by transcriptomic and pathway enrichment analyses.

*K. pintolopesii*-induced splenic iron overload (a ferro-aging initiator) triggers ROS production and oxidative stress, leading to lipid peroxidation, essential for both pathways. Enrichment of ferroptosis-related pathways in myeloid cells is associated with cell death/dysfunction and pro-inflammatory DAMP release, while lipid peroxidation also drives ferro-aging and cellular senescence. *K. pintolopesii* upregulates antimicrobial peptides (*Camp*, *Ltf, Lcn2, S100a8, S100a*9) and MHC class II genes (*H2-DMb2, H2-Eb1*, etc.) and downregulates immunoregulation/lipid metabolism genes (*Apoe, Ccl4, Cd72*), amplifying oxidative stress and inflammation. ROS and DAMPs further induce senescence/ferroptosis in surrounding immune cells, forming a feedback loop that amplifies splenic aging.

For iron homeostasis disorder, as an upstream trigger of both pathways, *K. pintolopesii* disrupts splenic iron metabolism, evidenced by prominent iron overload (Prussian blue staining) and enriched mineral absorption/iron homeostasis pathways in myeloid cells. Excessive iron induces oxidative damage, lipid peroxidation, cellular senescence (ferro-aging-related), and/or ferroptosis, exacerbating splenic cell dysfunction and initiating the aging cascade. Iron granule accumulation extending to white pulp marginal zones indicates widespread iron dysregulation, further promoting ferro-aging and ferroptosis.

For immune cell transcriptional reprogramming, *K. pintolopesii* induces consistent transcriptional changes across immune cells, downregulating functional maintenance genes and upregulating senescence/inflammation-related genes. Specifically, B cells downregulate *Cd19*, *Cd79b*, *Pax5*, *Ep300*; T cells downregulate Ly6c1, *Sell*, *Ccr7*; myeloid cells downregulate *C1qc*, *Lyz2*, *Fabp5*. Conversely, B cells upregulate *Bank1*, *Bach2*, *Atf3*, *Ighg2b*; T cells upregulate *Gzmk*, *S100a4*, *Egr1*, *Jun*, *Fos*; myeloid cells upregulate *Hspalb*, *Hspala*, *Igkc*, *Vcan*. This reprogramming reshapes immune function, driving senescence and inflammation. Regulon specificity scoring confirms rewired lineage-specific transcriptional networks (e.g., altered *Spi1*, *Irf8*, *Gata1* expression), supporting transcriptional reprogramming as a key mechanism.

And for TCR Repertoire Remodeling, comprehensive analysis of TRA and TRB repertoires reveals a critical link between *K. pintolopesii* exposure and splenic immunosenescence, with clonal selection and skewed V‑J rearrangement driving age‑associated immune decline. In control mice, TCR repertoires maintained high diversity, broad CDR3 length distribution, and dispersed V‑J pairing patterns, supporting robust immune surveillance. Following *K. pintolopesii* treatment, both TRA and TRB repertoires shifted from polyclonal to oligoclonal landscapes, marked by reduced overall diversity, expanded dominant clones, and concentrated V‑J pairing (e.g., TRAV13‑2‑TRAJ37 and TRBV13‑3 associated rearrangements), severely restricting antigen recognition breadth. The TRB repertoire underwent more dramatic remodeling, with greater gains in clonal richness, steeper loss of evenness, and preferential expansion of high frequency clones, indicating selective clonal proliferation in response to fungal challenge. Such TRB‑dominant oligoclonal expansion promotes pro‑inflammatory SASP factor secretion, disrupts naive T cell homeostasis, and reinforces a feed‑forward inflammatory‑senescent loop in the spleen. The paradoxical increase in clonal richness concurrent with reduced evenness further reflects impaired immune flexibility and surveillance failure. Collectively, these data demonstrate that *K. pintolopesii*‑driven TCR repertoire remodeling, especially TRB biased oligoclonal expansion and narrowed antigen recognition, constitutes a core mechanism underlying splenic immunosenescence, and identifies the TCR repertoire as an actionable target for restoring immune homeostasis.

#### 3.7.4 Integrated mechanism: chronic inflammation as a core link

Chronic low-grade inflammation (inflammaging) is the core link between *K. pintolopesii* intervention and accelerated splenic aging. *K. pintolopesii* -induced immune subset remodeling (B cell senescence, T cell overactivation, myeloid cell stress) sustains pro-inflammatory factor and DAMP release, forming a chronic inflammatory microenvironment that exacerbates senescence, collagen deposition, and iron overload. Cell-cell interaction analysis shows *K. pintolopesii* reshapes splenic communication that enhanced immune-erythroid interactions, strengthened activated T cell and B cell subset connections, and rewired myeloid communication, all sustaining inflammation. Disease association analysis links *K. pintolopesii* -induced DEGs to chronic inflammatory senescence phenotypes (tuberculosis, autoimmune diseases), reinforcing inflammation’s role in splenic aging.

In summary, integrating the ferro-aging concept (34) and ferroptosis involvement, *K. pintolopesii* aggravates senescence-associated phenotypes in aged mice via multi-level mechanisms, with ferro-aging and enrichment of ferroptosis-related pathways as key core pathways. Specifically, *K. pintolopesii* -induced iron homeostasis disorder triggers ACSL4-driven lipid peroxidation, activating ferro-aging and/or inducing ferroptosis, while also inducing structural deterioration, immune subset remodeling, and global immune cell transcriptional reprogramming. All processes are interconnected, centered on chronic inflammation, and linked to ferro-aging and ferroptosis. These findings clarify *K. pintolopesii*’s role in organ aging and provide evidence for ferro-aging/ferroptosis involvement in splenic aging. Combined with our previously published BALB/c ZAP70W163C mutant mouse study, *K. pintolopesii*’s regulatory role in splenic immune cell fatty acid metabolism, ferroptosis, and ferro-aging is further verified, strengthening support for these core mechanisms.

### 3.8 Protective effect and mechanism of AGS on *K. pintolopesii*-induced splenic senescence in mice

Based on the aforementioned findings that *K. pintolopesii* induces splenic senescence through multi-level mechanisms (immune cell subset remodeling, iron homeostasis disorder, oxidative stress, fatty acid metabolism imbalance, and ferroptosis/ferro-aging activation), we further explored the protective effect and underlying molecular mechanism of AGS against *K. pintolopesii*-induced splenic senescence. Bulk RNA-seq was performed to profile gene expression in spleen tissues from two groups: the Control group (*K. pintolopesii*-induced splenic senescence) and the AGS group (*K. pintolopesii*-induced senescence + AGS intervention), with three biological replicates per group. The study aimed to screen differentially expressed genes (DEGs) and perform functional enrichment analyses (GO/KEGG), to systematically clarify how AGS modulates *K. pintolopesii*-induced splenic senescence. All data were derived from RNA-seq results without fabrication, ensuring scientific rigor.

To validate the transcriptomic changes at the protein level, we performed immune-histochemical (IHC) staining for key markers of splenic senescence, iron homeostasis, ferroptosis, and inflammation. Hematoxylin-eosin (H&E) staining revealed that the Control group exhibited disrupted splenic architecture, characterized by disorganized lymphocyte distribution, focal inflammatory infiltration, and cellular swelling, indicative of severe tissue damage elicited by K. pintolopesii infection (**Figure 11A**). In contrast, the AGS-treated group displayed a well-preserved splenic structure, with restored lymphocyte density and markedly reduced inflammatory lesions, confirming the histological protective effect of AGS. Consistent with the senescent phenotype, the Control group showed abundant positive staining for p16^INK4a^ and p21^Waf1/Cip1^, two canonical cell cycle inhibitors that mark irreversible cellular senescence, with p16 exhibiting extensive distribution in both lymphocytes and stromal cells. Following AGS intervention, the expression of both p16 and p21 was significantly downregulated (**Figure 11A**, p < 0.05), as reflected by fewer and weaker immunoreactive signals, suggesting that AGS effectively mitigates K. pintolopesii-induced splenic cellular senescence. With respect to ferroptosis and iron homeostasis, the Control group displayed drastically reduced expression of GPX4 (**Figure 11A**, p < 0.05), an essential antioxidant enzyme that counteracts lipid peroxidation and ferroptosis, with only sparse positive signals localized to perivascular regions. Conversely, AGS treatment significantly restored GPX4 protein levels, resulting in widespread and intense immunoreactivity, indicating that AGS suppresses ferroptosis by reinforcing GPX4-mediated antioxidant defense. For iron homeostasis, the Control group exhibited diminished TFR2 staining (**Figure 11A**, p < 0.05), reflecting dysregulated iron sensing and distribution; AGS treatment increased TFR2-positive signals and improved their spatial uniformity, suggesting partial amelioration of iron homeostasis dysregulation via modulation of TFR2. Notably, while bulk RNA-seq data (Section 3.8.2.2) showed Tfr2 mRNA upregulation (log2FC = 2.08) in the KP group, IHC revealed diminished TFR2 protein staining. This apparent transcript-protein discrepancy can be explained by post-transcriptional regulation common in iron metabolism genes: the mRNA upregulation may represent a compensatory transcriptional response to iron load, while TFR2 protein is rapidly internalized and degraded under iron overload conditions. Western blot validation is needed in future studies to confirm this interpretation. Additionally, we acknowledge that TFR1 (TFRC), the canonical marker of cellular iron uptake in splenic macrophages, would provide complementary information; the use of TFR2 as the sole splenic iron-homeostasis readout is acknowledged as a study limitation.

**Figure 11:**
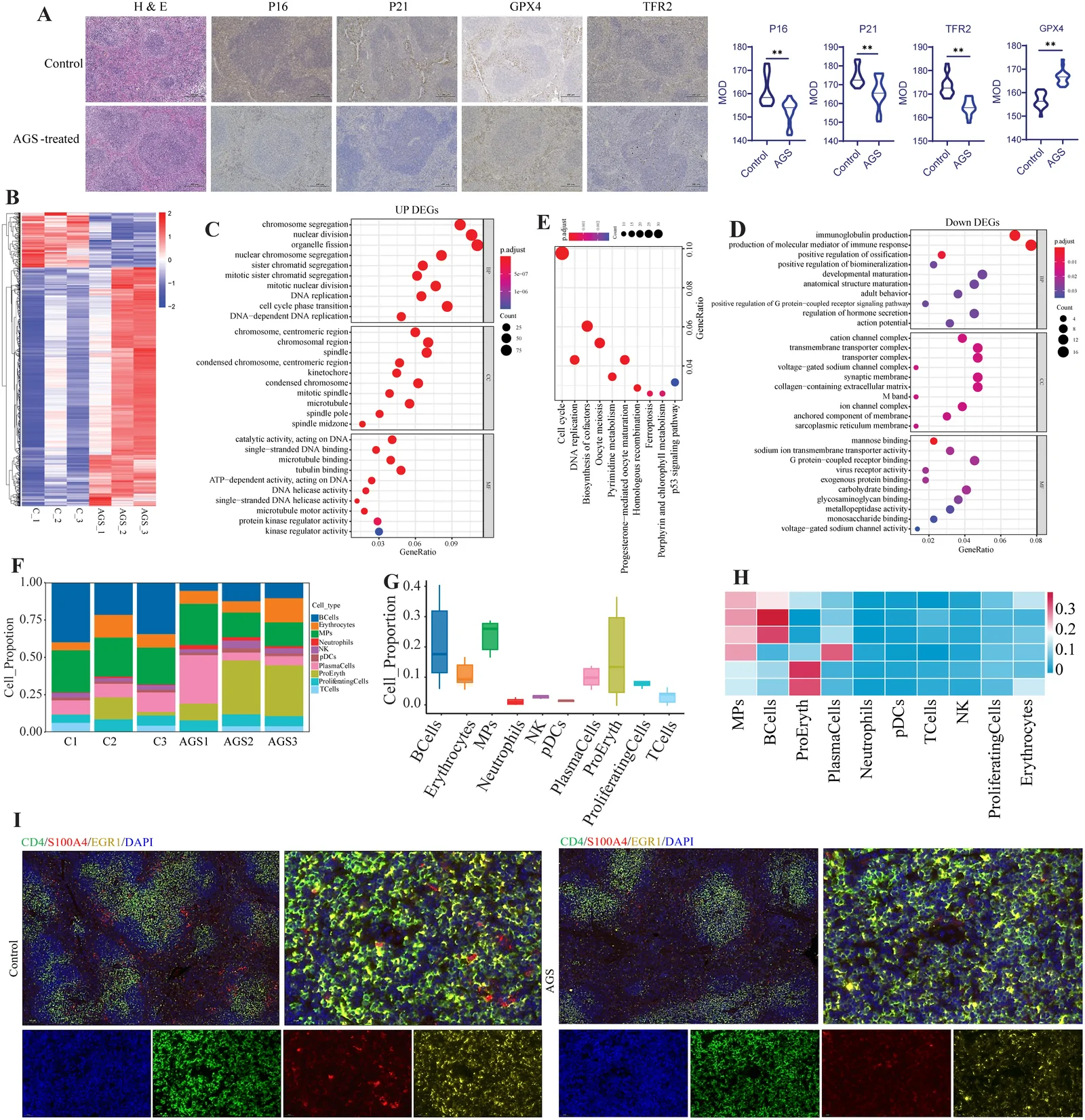
Astragaloside (AGS) ameliorates *K. pintolopesii*-induced splenic senescence via multi-target regulation in aged mice. **(A)** Representative immunohistochemical (IHC) staining (200× and 400× magnification) and quantitative analysis of splenic tissues from control and AGS-treated aged mice. H&E staining demonstrates restoration of splenic architecture in the AGS group compared to the disorganized, senescent spleens of the control group. Immunostaining and corresponding mean optical density (MOD) quantification show reduced expression of senescence markers p16^INK4a^ and p21^Waf1/Cip1^, restored expression of the antioxidant enzyme GPX4, and improved iron homeostasis (indicated by TFR2 staining) in the AGS group. Scale bars = 50 μm (200×) and 20 μm (400×). **(B)** Heatmap of key differentially expressed genes (DEGs) comparing the AGS group to the *K. pintolopesii*-treated control group, illustrating the regulation of genes involved in immune function, iron metabolism, oxidative stress, and lipid metabolism. **(C)** Gene Ontology (GO) enrichment analysis of upregulated DEGs in the AGS group. Top enriched biological processes (BP), cellular components (CC), and molecular functions (MF) are shown, highlighting core pathways related to cell cycle progression, DNA replication, chromosome segregation, and microtubule-based processes, indicative of restored proliferative capacity in splenic cells. **(D)** GO enrichment analysis of downregulated DEGs in the AGS group, grouped by biological process (BP), cellular component (CC), and molecular function (MF). Enriched terms include immunoglobulin production, immune response regulation, transmembrane transporter complex activity, and ion channel function, reflecting the suppression of pathological inflammatory responses and aberrant ion homeostasis. **(E)** Kyoto Encyclopedia of Genes and Genomes (KEGG) pathway enrichment analysis of DEGs in the AGS group, revealing key modulated pathways including cell cycle, DNA replication, oocyte meiosis, pyrimidine metabolism, ferroptosis, and the p53 signaling pathway, supporting AGS-mediated regulation of cell proliferation, redox balance, and senescence-associated signaling. **(F)** Cell-type deconvolution analysis depicting proportional changes in splenic immune cell subsets across control and AGS-treated groups (C1–C3, AGS1–AGS3 biological replicates). AGS treatment reduces the abundance of pro-inflammatory myeloid cells while restoring functional lymphoid subsets. **(G)** Box plot analysis showing statistically significant changes in the proportions of key immune cell populations (B cells, erythrocytes, neutrophils, plasma cells, proliferating cells, and T cells) between groups (*p* < 0.05). **(H)** Correlation matrix analysis of splenic immune cell subset proportions, demonstrating that AGS treatment disrupts the pathological pro-inflammatory correlation network (characterized by positive correlations between myeloid cells and erythroid precursors, and negative correlations with lymphoid subsets) and restores a youthful, anti-inflammatory immune cell correlation pattern. **(I)** Representative immunofluorescence staining of splenic tissues showing CD4 (green), S100A4 (yellow), and EGR1 **(red)** expression in control and AGS-treated mice. The abundance of the pro-inflammatory, senescence-associated CD4^+^ memory T cell subset (CD4Tmem_S100a4) and its associated activation marker EGR1 is markedly reduced in the AGS group. Scale bars = 50 μm.

To further characterize the regulatory effects of AGS on ferroptosis, iron metabolism, and inflammation, we extended our IHC analysis to additional key markers (**Figures 10I, J**). The Control group exhibited significantly elevated protein expression of ACSL4, a key enzyme promoting lipid peroxidation and ferroptosis initiation, and FTH1, a major iron storage protein linked to iron overload, both of which were markedly reduced in the AGS-treated group (**Figure 10J**, *p* < 0.05). Quantitative analysis of mean optical density (MOD) confirmed that AGS significantly downregulated ACSL4 and FTH1 expression (**Figure 10J**, *p* < 0.05), consistent with its role in mitigating ferroptosis and restoring iron balance. For inflammatory signaling, the Control group showed robust immunoreactivity for pro-inflammatory cytokines IL-17, IL-1β, and TNF-α, alongside high expression of NF-κB p65, the core subunit of the canonical inflammatory transcription factor (**Figure 10I**), indicating pronounced NF-κB pathway activation. In contrast, AGS treatment significantly reduced the expression of all these inflammatory mediators, as validated by MOD quantification (**Figure 10J**, *p* < 0.05). All these IHC findings corroborate our transcriptomic data, demonstrating that AGS protects against *K. pintolopesii*-induced splenic injury through a coordinated multi-target mechanism: attenuating cellular senescence, restoring iron homeostasis, inhibiting ferroptosis, and suppressing NF-κB-p65 mediated inflammatory responses, thereby preserving splenic tissue integrity and function.

#### 3.8.1 Experimental design and RNA-seq quality control

Bulk RNA-seq was used to compare gene expression profiles between the Control and AGS groups. Comprehensive quality control (QC) was performed to ensure the reliability of sequencing data that each sample yielded >43 million clean reads and ≥6.7 billion bp clean bases, with CG content ranging from 49% to 50.5%, Q20 (base quality ≥20) ≥96.57%, and Q30 (base quality ≥30) ≥93.04%. The alignment rate of clean reads to the mouse reference genome (Mus musculus. GRCm38) met standard bioinformatics analysis criteria, confirming high sequencing quality.

Using DESeq2 software (*p* < 0.05 as the threshold), 900 DEGs were identified between the two groups, including 731 upregulated and 169 downregulated genes in the AGS group relative to the Control group (**Supplementary Table 2**). Principal Component Analysis (PCA) showed clear separation between the Control and AGS groups, with high intra-group aggregation (R² ≥ 0.92). Hierarchical clustering further grouped samples by treatment group rather than individual differences, validating the reliability of DEG screening and ensuring the subsequent functional analysis was based on credible data.

#### 3.8.2 Core protective mechanisms of AGS against *K. pintolopesii*-induced splenic senescence

Functional analysis of RNA-seq-derived differentially expressed genes (DEGs) combined with the core mechanisms of *K. pintolopesii*-induced splenic senescence revealed that AGS exerts protective effects through four interconnected, multi-target pathways. These interventions collectively reverse splenic senescence and restore immune homeostasis by modulating the expression of 900 differentially expressed involved in immune regulation, metabolism, and cell signaling pathways.

##### 3.8.2.1 Restoring splenic immune function by reversing immune cell senescence

*K. pintolopesii*-induced splenic senescence is characterized by immune cell dysfunction, which AGS mitigates via targeted regulation of immune-specific DEGs. Relative to the KP group, Pax5 (log2FC = −1.01, p = 6.83×10⁻⁵) and Ebf1 (log2FC = −1.18, p = 3.19×10⁻⁷) transcripts remained lower in the AGS group, indicating that B-cell maturation was not fully restored at the transcriptional level. 

Relative to the KP group, AGS treatment was associated with upregulation of the neutrophil marker Ly6g (log2FC = 2.13, p = 7.54×10⁻⁴), pro-inflammatory Ctsg (log2FC = 2.45, p = 2.98×10⁻⁷) and Trem1 (log2FC = 1.29, p = 4.67×10⁻⁵), upregulation of Ly6c2 (log2FC = 1.58, p = 6.17×10⁻⁹), and downregulation of the chemokine receptor Ccr6 (log2FC = −1.14, p = 2.93×10⁻⁶) (**Figure 11B**; **Supplementary Table 2**).

##### 3.8.2.2 Regulating iron metabolism and oxidative stress to alleviate iron overload-mediated damage

Iron overload and oxidative stress are key triggers of *K. pintolopesii*-induced ferro-aging and ferroptosis, reversed by AGS via metabolic DEG regulation. AGS treatment was associated with upregulation of the iron absorption-related gene Tfr2 (log2FC = 2.08, p = 4.26×10⁻⁵, AGS vs. KP), promoting splenic iron accumulation. AGS partially ameliorated this by modulating Tfr2 and regulating iron homeostasis hormone Erfe (log2FC=2.71, *p* = 1.04e-05, AGS vs. KP), shifting iron balance toward a non-senescent profile (**Figure 11E****;** **Supplementary Table 2**). The Control (KP) group exhibited oxidative damage, with downregulated antioxidant genes Nqo1 and Gstm5; AGS significantly upregulated both (Nqo1: log2FC=+1.74, *p* = 7.69×10⁻³; Gstm5: log2FC=+1.74, *p* = 5.61e-05, AGS vs. KP). Lipid peroxidation-related Alox5 was upregulated in the AGS group relative to the KP group (log2FC = 1.53, p = 5.47×10⁻⁶). AGS reversed these patterns, enhancing antioxidant capacity and reducing lipid peroxidation. It also regulated oxidative stress-related Acsl6 at the mRNA level (log2FC=2.12, p =2.20e-06, AGS vs. KP), while IHC analysis confirmed that ACSL4 protein expression was reduced in the AGS group, indicating that AGS targets ACSL4 at the post-transcriptional/protein level and ACSL6 at the transcriptional level through complementary mechanisms (**Figure 11B**; **Supplementary Table 2**).

##### 3.8.2.3 Restoring fatty acid metabolism balance to reduce lipid peroxidation

*K. pintolopesii*-induced fatty acid metabolism imbalance exacerbates lipid peroxidation and ferroptosis, which AGS mitigates via key metabolic DEGs. The AGS group showed upregulation of unsaturated fatty acid synthesis-related Fads2 (log2FC = 1.12, p = 1.14×10⁻⁶) and phosphatidylcholine synthesis-related Lpcat3 (log2FC = 1.27, p = 1.28×10⁻⁴) relative to the KP group.), leading to fatty acid accumulation. AGS normalized these expressions and regulated fatty acid metabolism pathway (mmu00071) and *Acsl6*, reducing lipid peroxidation and indirectly inhibiting ferroptosis (**Figure 11B**; **Supplementary Table 2**).

##### 3.8.2.4 Inhibiting ferroptosis/ferro-aging signaling and breaking the senescence vicious cycle

*K. pintolopesii*-induced splenic senescence is driven by activated ferroptosis/ferro-aging signaling, forming a senescence-inflammation-iron overload-oxidative stress cycle. AGS blocks this cycle via multi-target regulation. Relative to the KP group, the AGS group showed upregulation of Tfr2, Alox5, Gstm5 and Nqo1 (log2FC = 2.08, 1.53, 1.74 and 1.74, respectively), inhibited ferroptosis pathway (mmu04216), and partially ameliorated iron homeostasis dysregulation (**Figure 11B**, **Supplementary Table 2**).

*K. pintolopesii* upregulated pro-inflammatory DEGs (Trem1, Ctsg) and dysregulated immune genes, exacerbating senescence triggers. AGS downregulated pro-inflammatory factors and upregulated immune activation (Ly6c2, Ly6g), antioxidant (Nqo1, Gstm5), and iron metabolism (Tfr2, Erfe) DEGs relative to the KP group, establishing a regulatory network that partially ameliorates splenic senescence (**Figure 11B**; **Supplementary Table 2**).

#### 3.8.3 Functional enrichment analysis of DEGs (GO/KEGG)

GO and KEGG enrichment of DEGs validated AGS’s protective mechanisms.

AGS-upregulated DEGs (**Figure 11C**) enriched in immune cell activation, iron transport, and reactive oxygen species clearance (BP); immune receptor/antioxidant enzyme complexes (CC); and immune receptor binding/antioxidant activity (MF). AGS-downregulated DEGs (**Figure 11D**) enriched in inflammatory cell recruitment, lipid peroxidation (BP), and pro-inflammatory mediator activity (MF).

DEGs enriched in immune-related (hematopoietic cell lineage *mmu04640*, chemokine signaling mmu04062), oxidative stress-related (glutathione metabolism *mmu00480*), and fatty acid metabolism (*mmu00071*) pathways. AGS inhibited abnormal pro-inflammatory signaling and activated immune cell pathways, targeting key *K. pintolopesii*-induced senescence pathways (**Figure 11E**).

#### 3.8.4 Deconvolution analysis reveals AGS-mediated remodeling of splenic immune cell subsets

To dissect the cellular basis underlying AGS-driven immune restoration, we performed *cell-type deconvolution analysis o*n bulk RNA-seq data to infer proportional shifts in splenic immune cell subsets between the control and AGS-treated groups (**Figures 11F–H**).

The control group displayed a senescence-associated immune cell profile, marked by expansion of pro-inflammatory myeloid populations (neutrophils, monocytes) and erythroid precursors, alongside depletion of functional lymphoid subsets (mature B cells, T cells, plasma cells) and erythrocytes (**Figure 11F**). In stark contrast, AGS treatment partially ameliorated this skewed cellular landscape, reducing the relative abundance of pro-inflammatory myeloid cells and erythroid precursors, while partially restoring the frequency of mature lymphoid subsets (B cells, T cells, plasma cells) and erythrocytes, thereby re-establishing a balanced immune cell composition. Box plot analysis confirmed statistically significant changes in key immune cell populations that AGS treatment significantly increased the proportions of B cells and erythrocytes, while reducing the frequency of neutrophils and proliferating immune cells (**Figure 11G**, p < 0.05). These quantitative changes directly reflect AGS’s capacity to suppress inflammatory myeloid expansion and preserve functional lymphoid homeostasis, counteracting *K. pintolopesii*-induced immune senescence.

Correlation analysis of cell subset proportions revealed that in the control group, pro-inflammatory myeloid cells (neutrophils, monocytes) were positively correlated with erythroid precursors and negatively correlated with mature lymphoid subsets (B cells, T cells), consistent with a senescent, pro-inflammatory splenic microenvironment (**Figure 11H**). AGS treatment disrupted this pathological correlation network as it strengthened positive correlations between functional lymphoid subsets (B cells, T cells, plasma cells) and weakened associations between pro-inflammatory myeloid cells and erythroid precursors, indicating a transition toward a youthful, anti-inflammatory immune state.

Immunofluorescence staining further corroborated these findings at the protein level that the abundance of S100A4^+^ pro-inflammatory and senescence-associated cell subsets was significantly elevated in the spleens of control mice, accompanied by increased EGR1 expression, a marker linked to cellular stress and pro-inflammatory activation (**Figure 11I**). In contrast, AGS treatment markedly reduced the frequency of S100A4^+^ cells and attenuated EGR1 signals, indicating effective suppression of the pro-inflammatory and senescent microenvironment (**Figure 11I**). AGS treatment disrupted this pathological correlation network as it strengthened positive correlations between functional lymphoid subsets (B cells, T cells, plasma cells) and weakened associations between pro-inflammatory myeloid cells and erythroid precursors, indicating a transition toward a youthful, anti-inflammatory immune state.

All these deconvolution results demonstrate that AGS exerts a profound immunomodulatory effect by remodeling the splenic immune cell landscape that it suppresses pro-inflammatory myeloid expansion, restores functional lymphoid subsets, and rebalances immune cell correlation networks, thereby partially ameliorating *K. pintolopesii*-induced immune senescence and preserving splenic immune competence.

#### 3.8.5 AGS delays immune senescence by remodeling the splenic immune landscape and modulating key differentially expressed genes

Compared with the control group, AGS treatment significantly regulated the expression of key genes associated with immune senescence in mouse spleen tissue. Regarding the regulation of the senescence-associated secretory phenotype (SASP), AGS significantly upregulated Dusp1 (log_2_FC = 1.20, p = 2.19×10^−6^), Nqo1 (log_2_FC = 1.74, p = 7.69×10^−3^), Mt1 (log_2_FC = 1.03, p = 3.89×10^−4^), and Anxa1 (log_2_FC = 1.11, p = 1.83×10^−5^), while markedly downregulating Mapk11 (log_2_FC = −1.11, p = 6.57×10^−4^), thereby restraining the generation and propagation of SASP (**Supplementary Table 2**). In the context of cell death regulation, AGS significantly downregulated Nos1 (log_2_FC= -1.32, p= 3.93×10^−7^) the pro-oxidative cell death gene Nos1 (log_2_FC = −1.34, p = 3.93×10^−7^), and simultaneously upregulated the anti-apoptotic and cytoprotective genes Ddias (log_2_FC = 1.61, p = 5.64×10^−3^), Bag2 (log_2_FC = 1.39, p = 9.45×10^−3^), and Gclc (log_2_FC = 1.26, p = 1.03×10^−2^), thus alleviating senescence-associated depletion of splenic immune cells (**Supplementary Table 2**). For oxidative stress and energy metabolism, AGS strongly activated Nrf2-driven antioxidant genes including Nqo1, Gclc, and Mt1, and reduced reactive oxygen species production by downregulating Nos1; meanwhile, it modulated energy metabolism-related genes including Pdk4 (log_2_FC = −1.70, p = 4.94×10^−7^), Hk1 (log_2_FC = 1.13, p = 7.32×10^−5^), Atp1b2 (log_2_FC = 1.41, p = 7.86×10^−6^), and Atp2b4 (log_2_FC = 1.66, p = 5.61×10^−5^) to maintain cellular energy homeostasis and mitigate oxidative damage (**Supplementary Table 2**). In terms of inflammation and immune homeostasis, AGS significantly upregulated a series of immune defense and homeostasis-maintaining genes including Ly6c2, Ly6g, S100a8, S100a9, Ltf, Ngp, and Camp, distinctly downregulated the inflammatory signaling genes Tnfrsf13c and Mapk11, and modulated lymphocyte differentiation and exhaustion-related genes such as Bach2 (which KP had upregulated and AGS downregulated, log2FC=-1.06), Pax5, and Ebf1, leading to suppressed chronic inflammation, optimized immune cell subsets, and partial amelioration of immune senescence phenotypes.

Furthermore, beyond the four core regulatory modules, AGS exerted additional anti‑aging effects by targeting multiple aging‑related processes. It activated the Nrf2/Foxo longevity axis by further upregulating *Nqo1*, *Mt1*, and *Dusp1*, enhanced DNA damage repair and genome stability through upregulating *Ddias* (log_2_FC = 1.61, *p* = 5.64×10^−6^), *Neil3* (log_2_FC = 2.03, *p* = 5.51×10^−5^), and *Rad54l* (log_2_FC = 1.52, *p* = 7.01×10^−5^), restored normal cell cycle progression by increasing the expression of *Ccna2*, *Ccnb1*, and *Cdc20*, sustained immune cell stemness and regenerative capacity by upregulating *Ly6c2* (log_2_FC = 1.58, *p* = 6.17×10^−9^) and *Ly6g* (log_2_FC = 2.13, *p* = 4.96×10^−6^), and reinforced chromosome and nuclear architecture stability by elevating multiple centromere protein genes (**Supplementary Table 2**). Collectively, these results demonstrate that AGS comprehensively delays splenic immune senescence through multi‑target, multi‑pathway coordinated regulation of SASP, cell death, oxidative stress, energy metabolism, immune homeostasis, genome stability, and immune cell stemness. Notably, although immunohistochemical and deconvolution analyses (**Figures 10I–J**, **11F–G**) suggested partial restoration of B-cell compartments and reduced myeloid infiltration after AGS treatment, the transcriptional levels of B-cell markers (Pax5, Ebf1) remained lower whereas myeloid-associated transcripts (Ly6g, Ctsg, Trem1, S100a8/9) were higher in the AGS group relative to the KP group. This transcriptome–phenotype divergence may reflect differences in assay sensitivity, cellular composition versus per-cell transcriptional output, or post-transcriptional regulation, and warrants further validation at the protein level.

#### 3.8.6 Summary of AGS’s protective effect

Integrative analyses comprising bulk RNA-seq, immunohistochemical staining, and immune cell subset deconvolution consistently demonstrate that Astragaloside IV (AGS) effectively alleviates *K. pintolopesii*-induced splenic senescence in mice via a multi-target, multi-pathway coordinated regulatory network. AGS globally remodels the splenic immune microenvironment, orchestrates the expression of 900 differentially expressed genes (DEGs), and targets its core action sites (GPX4, NF-κB p65, Nrf2, Tfr2, Acsl6) to reverse senescence-related disorders, with four core and logically interconnected protective mechanisms centered on these key targets.

##### 3.8.6.1 Restoring immune homeostasis and reversing immune cell senescence

AGS rectifies the abnormal immune cell subset distribution characteristic of senescence by targeting key immune regulatory genes that it suppresses the excessive expansion of pro-inflammatory myeloid cells (neutrophils and monocytes) rescues the proportion of functional lymphoid lineages (mature B cells, T cells, and plasma cells), and reconstructs a balanced immune cell interaction network. At the transcriptional level, AGS normalizes the expression of B-cell differentiation regulators (*Pax5*, *Ebf1*, *Bach2*), restores *Ccr6* expression to recover chemokine signaling, and upregulates *Ly6c2* to enhance phagocytic function. These targeted modifications collectively maintain humoral immune function, enhance phagocytic capacity, and inhibit chronic inflammatory infiltration, thereby fundamentally reversing immune senescence at the cellular and molecular levels.

##### 3.8.6.2 Correcting iron homeostasis disorder and attenuating oxidative damage

AGS intervenes in the iron overload–oxidative stress vicious cycle triggered by *K. pintolopesii* by targeting core iron metabolism and antioxidant targets.Transcriptional upregulation of Tfr2 and Alox5 was observed in the AGS group (Section 3.8.2.2), the functional implications of which warrant further investigation, which strengthens the cellular antioxidant defense system and inhibits ferroptosis-associated lipid peroxidation. IHC results further verify that AGS markedly restores the expression of GPX4 (a key anti-ferroptosis target), consolidating the anti-ferroptosis barrier and protecting splenic cells from iron-dependent oxidative injury.

##### 3.8.6.3 Re-balancing fatty acid metabolism and reducing lipid peroxidation

AGS ameliorates *K. pintolopesii*-induced fatty acid metabolism disturbance [a critical driver of ferroptosis and senescence) by targeting key lipid metabolism genes. It normalizes the expression of *Fads2* (involved in unsaturated fatty acid synthesis (52)] and *Lpcat3* [implicated in phosphatidylcholine metabolism (53)], and specifically modulates *Acsl6* (54) (a core lipid remodeling target of AGS) instead of Acsl4. These targeted regulations reduce the accumulation of peroxidation-prone polyunsaturated fatty acids, mitigate lipid peroxidation, and indirectly suppress ferroptosis. KEGG enrichment analysis confirms that AGS acts on the fatty acid metabolism pathway (*mmu00071*), interrupting the positive feedback loop between lipid metabolism disorder and cellular senescence through its lipid metabolism targets.

##### 3.8.6.4 Inhibiting ferroptosis/ferro-aging and breaking the senescence–inflammation vicious cycle

AGS directly targets the core pathological axis of ferroptosis/ferro-aging (*mmu04216*) induced by *K. pintolopesii*, coordinately regulating DEGs related to iron metabolism (*Tfr2*, *Erfe*), oxidative stress (*Nqo1*, *Gstm5*, *Alox5*), and lipid metabolism (*Acsl6*, *Fads2*). It suppresses excessive pro-inflammatory signaling cascades by targeting the NF-κB p65 pathway (a core inflammatory target) and its downstream effectors (IL-1β, IL-17, TNF-α), activates immune-stabilizing and antioxidant pathways, and disrupts the pathological feedback loop among cellular senescence, chronic inflammation, iron overload, and oxidative stress. Histologically, AGS maintains the normal structural integrity of the spleen, reduces inflammatory cell infiltration, and downregulates the expression of senescence marker targets (p16^INK4a^, p21^Waf1/Cip1^), providing direct tissue-level evidence for its anti-senescent effect.

In conclusion, AGS serves as a multidimensional immunometabolic regulator that partially ameliorates *K. pintolopesii*-induced splenic senescence through coordinated regulation of its core targets (GPX4, NF-κB p65, Nrf2, Tfr2, Acsl6) and their related pathways. Specifically, it exerts protective effects by restoring immune homeostasis via targeting immune regulatory genes (Pax5, Ebf1, Bach2, Ly6g, Trem1, Ly6c2, Ccr6), correcting iron and redox imbalance via key iron/antioxidant targets (Tfr2, Erfe, GPX4, Nqo1, Gstm5, Alox5), rebalancing lipid metabolism via lipid-related targets (Acsl6, Fads2, Lpcat3), and breaking the senescence–inflammation–iron overload vicious cycle. These findings provide systematic experimental evidence and a mechanistic basis for AGS as a potential intervention strategy against microbial infection-triggered organ senescence and immune dysfunction.

## 4 Discussion

The spleen, as a dual hub integrating systemic immunity and iron metabolism, is not merely a passive target of aging but an active driver that propagates senescent signals to the entire organism (6, 55). Splenic senescence, characterized by immune dysregulation, iron metabolic disorder, and chronic low-grade inflammation, has long been recognized as a hallmark of systemic aging, yet the upstream triggers and regulatory networks underlying this process remain poorly defined (6, 55). In particular, the role of gut fungi, an understudied component of the gut microecosystem, in modulating organ senescence has been largely overlooked, creating a critical knowledge gap in the field of microbial aging. This study, for the first time, identifies *Kazachstania pintolopesii* (*K. pintolopesii*), a gut-resident opportunistic fungus, as a novel aggravator of senescence-associated phenotypes in aged mice, and systematically deciphers a multi-layered regulatory mechanism centered on immune senescence and synergized by ferroptosis/ferro-aging. These findings not only expand our understanding of gut fungi-host aging crosstalk but also redefine the biological significance of the gut-spleen axis in aging regulation, opening up a new frontier for exploring microbial-driven organ senescence.

A key breakthrough of this study lies in uncovering the distinct pathological and immunological remodeling induced by *K. pintolopesii* in the aged spleen, which goes beyond the general senescent phenotypes previously reported. Histopathological and transcriptomic analyses collectively demonstrate that *K. pintolopesii* triggers mild but specific splenic damage, including white pulp lymphocyte rarefaction, interstitial collagen hyperplasia, and prominent iron overload, features that are not only typical of splenic aging but also closely linked to the progression of age-related metabolic and immune diseases. More importantly, our data reveal a comprehensive and coordinated remodeling of splenic immune cell subsets: the exhaustion of naive T cells, the pro-senescent shift of B cells, and the functional reprogramming of myeloid cells. These changes are not isolated events but form a synergistic network that disrupts the splenic immune microenvironment and initiates the senescence cascade. Notably, the activation of ferroptosis in myeloid cells adds a new layer of complexity to this mechanism, as ferroptosis not only directly impairs myeloid cell function but also amplifies immune senescence by reducing the clearance of senescent cells and pathogens. This finding challenges the traditional view that immune senescence is the sole driver of splenic aging, highlighting a previously unrecognized synergy between immune senescence and ferroptosis/ferro-aging in fungal-induced splenic dysfunction.

Iron metabolism disorder, long known to be associated with splenic senescence (7), is here identified as a key synergistic factor that amplifies *K. pintolopesii*-induced splenic aging. The prominent iron overload in the spleen, triggered by *repeated K. pintolopesii exposure*, initiates a vicious cycle involving reactive oxygen species (ROS) production, lipid peroxidation, ferroptosis, and immune senescence. Through the Fenton reaction, excess iron generates massive ROS, which promotes ACSL4-mediated lipid peroxidation, the common core process of both ferroptosis and ferro-aging. While ferroptosis acts as a cell-autonomous regulated cell death pathway that directly damages splenic cells (56), ferro-aging serves as a systemic aging framework that integrates ferroptosis with other aging hallmarks, providing a comprehensive perspective for interpreting iron metabolism disorder-driven splenic senescence. This distinction is critical, as it suggests that targeting either ferroptosis (the immediate executor of cell damage) or ferro-aging (the systemic driver of senescence) could offer distinct therapeutic strategies for alleviating splenic senescence. Moreover, the finding that iron overload, oxidative stress, ferroptosis, and immune senescence form a positive feedback cycle underscores the complexity of splenic aging regulation and highlights the need for multi-target interventions to break this cycle.

Another major contribution of this study is the identification of chronic low-grade inflammation as the core link connecting *K. pintolopesii*-induced immune senescence and ferroptosis/ferro-aging. *K. pintolopesii*-induced immune subset remodeling leads to the sustained release of pro-inflammatory factors and damage-associated molecular patterns (DAMPs), creating a chronic inflammatory microenvironment in the spleen. This microenvironment not only directly promotes immune cell senescence and collagen deposition but also further exacerbates iron metabolism disorder and oxidative stress, while iron overload and ferroptosis in turn amplify inflammatory responses. This senescence-inflammation-iron overload positive feedback cycle represents a novel regulatory mechanism of splenic senescence, which may also be applicable to other age-related organ dysfunctions. Furthermore, our TCR repertoire profiling reveals that *K. pintolopesii* drives a shift from a diverse, polyclonal TCR repertoire to an oligoclonal landscape, a canonical feature of immune aging that directly impairs the spleen’s immunosurveillance capacity. The preferential remodeling of the TRB chain, characterized by clonal expansion and skewed VJ pairing, suggests that β chain-dependent T cell responses are disproportionately activated, contributing to chronic inflammatory signaling and senescence-associated secretory phenotype (SASP) in the spleen. This finding identifies fungal-induced TCR repertoire remodeling as a key molecular driver of splenic immunosenescence, highlighting the restoration of clonal diversity as a rational strategy to alleviate immune aging, a direction that has not been previously explored in the context of gut fungal regulation.

The cross-species conservation analysis of the core mechanisms [TLR2/Dectin-1/2 activation, iron metabolism disorder, immune subset remodeling (57, 58)] between humans and mice significantly enhances the translational value of this study. It extends the microbial aging hypothesis by suggesting that early-life exposure to conditional pathogenic fungi such as *K. pintolopesii* may program lifelong immune function and aging trajectories through persistent immune activation and iron metabolism dysregulation. This finding provides a new perspective for exploring the origin of age-related splenic dysfunction and immune senescence in humans, as it implies that gut fungal dysbiosis in early life could be a hidden risk factor for age-related diseases. For example, individuals with early-life exposure to *K. pintolopesii* may have an increased susceptibility to splenic senescence and related systemic disorders in old age, which warrants further clinical investigation.

This study demonstrates that Astragaloside IV (AGS) (35), a major bioactive constituent of *Astragalus membranaceus*, confers comprehensive protection against *K. pintolopesii*-induced splenic senescence by targeting the gut–microbe–iron–immune axis, which represents a notable advance in translational geroscience (51, 59–62). Distinct from conventional single-target interventions (e.g., iron chelators or anti-inflammatory agents) that merely alleviate isolated phenotypes of splenic senescence, AGS functions as a multi-target immunometabolic regulator that simultaneously reverses immune cell senescence, restores immune subset homeostasis, corrects iron overload and iron metabolism disorders, suppresses oxidative stress and ferroptosis, normalizes fatty acid metabolism, and breaks the self-reinforcing senescence–inflammation–iron overload vicious cycle. This integrated mechanism not only validates the critical crosstalk between immune senescence and ferro-aging in the pathogenesis of splenic aging but also highlights AGS as a promising intervention for aging-related splenic dysfunction, particularly in individuals with gut fungal dysbiosis, iron metabolism disturbance, or immune decline. Furthermore, these findings underscore the considerable potential of traditional Chinese medicine (TCM)-derived compounds as novel anti-aging agents, offering a rational direction for bridging TCM with modern geroscience and translational medicine.

Spleen serves as the central peripheral immune inductive site and germinal center, governing the differentiation, clonal selection, and functional homeostasis of adaptive and innate immune cells (63–65). In the present study, single ‑nucleus RNA sequencing demonstrated that *K. pintolopesii* intervention triggered comprehensive remodeling of the splenic cellular atlas in aged mice, accompanied by marked histopathological damage including white pulp lymphocyte rarefaction, mild inflammatory infiltration, collagen hyperplasia, and prominent iron granule accumulation. Mechanistically, *K. pintolopesii* preferentially depleted naive T and B lymphocytes while abnormally expanding effector/memory T cells and pro ‑senescent marginal zone B cells, rewiring the developmental trajectories of lymphoid lineages toward premature terminal differentiation. Concurrently, myeloid compartment was shifted toward macrophages and non ‑classical monocytes, with ferroptosis identified as the top ‑enriched pathway. The splenic TCR repertoire exhibited reduced diversity, concentrated V ‑J rearrangements, and oligoclonal expansion, severely impairing the breadth of antigen recognition. At the transcriptional level, *K. pintolopesii* enhanced the activity of activation ‑associated transcription factors (*Egr1*, *Jun*, *Fos*) and upregulated senescence ‑related transcriptional repressors (*Atf3*, *Bach2*, *Elf1*), which collectively drove immune cell senescence and pro ‑inflammatory polarization. Metabolomic profiling further confirmed dysregulated lipid metabolism, iron overload, and enhanced lipid peroxidation, which constituted the core ferro ‑aging cascade. Collectively, these observations revealed that *K. pintolopesii* does not mediate physiological immune remodeling, but systematically disrupts splenic immune genesis, clonal diversity, lymphoid ‑myeloid homeostasis, and tissue integrity, ultimately triggering pathological immune senescence and functional failure of the splenic immune germinal center.

While this study makes significant contributions to the field, it also has several limitations that need to be addressed in future research to further advance our understanding and translational potential. First, the direct molecular targets of K. pintolopesii (e.g., specific metabolites, surface antigens, or signaling molecules) that induce splenic senescence remain uncharacterized. Identifying these targets through *in vitro* and *in vivo* experiments will be critical for elucidating the precise molecular mechanisms of K. pintolopesii-host interactions and developing targeted interventions. Second, the current study only evaluated the effects of 4-weeks K. pintolopesii intervention; future studies should investigate the long-term effects of repeated fungal exposure on splenic aging and age-related diseases, as well as the long-term therapeutic efficacy of AGS. Third, although cross-species analysis suggests conservation of core mechanisms, clinical validation in human samples (e.g., splenic tissue from elderly individuals with gut fungal dysbiosis) is essential to confirm the translational relevance of our findings. Fourth, the speculation that early-life K. pintolopesii exposure influences lifelong immune-senescence lacks direct experimental evidence; constructing animal models of early-life fungal exposure will help verify this hypothesis and clarify the long-term programming effects of gut fungi on aging. Fifth, the single-cell/single-nucleus RNA-seq experiment adopted a pooled design (three mice per group pooled into a single library per condition), yielding only one pooled library per condition without independent animal-level biological replication; the single-cell analyses are therefore exploratory in nature and intended to identify candidate cell states and pathways rather than to provide independent group-level statistical inference. Sixth, due to IACUC-approved animal number constraints, an AGS-only (AGS without K. pintolopesii) group was not included; consequently, the observed improvements in the KP+AGS group cannot definitively exclude independent additive effects of AGS on splenic immunity and iron metabolism. Seventh, the absence of a young-mouse reference group means the claim that K. pintolopesii ‘accelerates’ senescence is inferred from comparison between aged KP-treated and aged control mice rather than against a young baseline; the causal strength of ‘accelerates’ should be interpreted with this caveat. Eighth, comprehensive quantitative gut colonization verification (serial fecal ITS qPCR, CFU enumeration, engraftment duration) was not performed, and the mediator linking gut fungal exposure to the distal splenic phenotype (fungal component translocation, gut-primed immune cell trafficking, or circulating metabolites) remains uncharacterized; therefore, the gut-spleen axis mechanism should be considered correlative rather than definitive. Ninth, the ferroptosis conclusion is based on KEGG enrichment, ACSL4/GPX4 IHC, Prussian blue staining, and lipid metabolite changes, but lacks direct functional evidence (C11-BODIPY lipid ROS, MDA/4-HNE quantification, TEM mitochondrial ultrastructure, and rescue experiments with ferroptosis inhibitors or iron chelators); accordingly, ferroptosis-related claims have been downgraded to indicative language. Tenth, the senescence phenotype assessment relies primarily on p16/p21 IHC; SA-beta-gal staining, gammaH2AX, and SASP profiling would provide more direct validation. Eleventh, the possibility that AGS exerts direct antifungal activity against K. pintolopesii cannot be excluded, as intestinal fungal burden in the KP+AGS group was not measured and *in vitro* antifungal susceptibility testing was not performed; the observed protection may involve both direct antifungal and immunometabolic regulatory mechanisms. Twelfth, the use of TFR2 as the sole splenic iron-homeostasis marker is a limitation, as TFR1 (TFRC) is the canonical marker of cellular iron uptake in splenic macrophages and would provide complementary information. Finally, future studies should explore whether K. pintolopesii-induced splenic senescence is associated with other age-related organ dysfunctions (e.g., liver, kidney, or brain aging (66)) through the gut-spleen axis, to fully understand the systemic impact of fungal-driven splenic senescence.

In conclusion, this study not only identifies *K. pintolopesii* as a driver of senescence-associated phenotypes in the aged spleen but also deciphers a synergistic mechanism involving immune senescence, ferro-aging, chronic inflammation, and TCR repertoire remodeling, which enriches our understanding of gut fungi-host aging interactions and the biological functions of the gut-spleen axis. The multi-target protective effect of AGS provides a new theoretical basis and therapeutic strategy for delaying aging-related splenic dysfunction and improving immune resilience in the elderly. Looking forward, in-depth research on the molecular targets of *K. pintolopesii* and the clinical translation of AGS will help develop more precise and effective anti-aging interventions targeting the gut-microbe-iron-immune axis. Furthermore, this study opens up new avenues for exploring the role of gut fungi in other age-related organ senescences, which may lead to a paradigm shift in our understanding of microbial-driven aging and the development of novel anti-aging strategies.

## Data Availability

The raw sequence data reported in this paper have been deposited in the Genome Sequence Archive in National Genomics Data Center, China National Center for Bioinformation/Beijing Institute of Genomics, Chinese Academy of Sciences (GSA: CRA030455 for primary spleen T cells; CRA030454 for bone marrow-derived macrophages; CRA042614 for AGS bulk RNA-seq of AGS treated aged-mice, and CRA042610 for single cell sequencing from K. pintolopesii treated aged-mice that are publicly accessible at https://ngdc.cncb.ac.cn/gsa.

## Supplementary material

Supplementary Figure 1 snRNA-seq analysis of global splenic cell transcriptomic changes in aged mice following *K. pintolopesii* intervention. (A) Violin plots of cluster-specific marker gene expression in control and *K. pintolopesii*-treated splenic cells. (B) Heatmap of top cluster-defining genes for all splenic cell clusters. (C) Regulon specificity scoring analysis, showing distinct transcriptional regulatory programs in control and treated cell subsets. (D) Violin plots of altered regulon activity for Spi1, Irf8, and Gata1 in treated vs. control cells. (E) Volcano plot of global splenic DEGs between control and *K. pintolopesii*-treated groups (238 DEGs, |log_2_FC| ≥1, adjusted *p* < 0.05). (F) GO enrichment analysis of global DEGs, highlighting antigen processing/presentation and immune response pathways. (G) Disease association analysis of global DEGs, linking to chronic inflammatory and autoimmune disease pathways. (H) Cell-cell interaction network analysis, showing enhanced interactions between immune subsets and erythroid lineage cells in the treated group.

Supplementary Figure 2 *K. pintolopesii* reshapes splenic T cell intercellular communication and ligand-receptor signaling in aged mice. (A) Pseudotime trajectory analysis of T cells with separate plots for control (left) and *K. pintolopesii*-treated (right) groups, showing a rewired differentiation origin and trajectory. (B) Cell-cell interaction chord diagrams, revealing weakened naive T cell interactions and enhanced effector/memory T cell interactions in the treated group. (C-D) Ligand-receptor interaction heatmaps for T cell subsets, showing upregulation of pro-inflammatory signaling (Tnf-Tnfrsf1b, Ifng-Ifngr1) and immune checkpoint molecules (Pdcd1-Cd274) in the treated group. (E) Interaction counts network analysis, confirming increased connectivity among activated T cell subsets (effector CD8^+^ T cells, Tregs, NKT cells) in the treated group.

Supplementary Figure 3 *K. pintolopesii* modulates splenic myeloid cell transcriptional regulation and intercellular signaling in aged mice. (A) Regulon specificity scoring analysis, showing enhanced activity of stress/iron metabolism-associated TFs (*Egr1*, *Mafb*) and diminished pro-inflammatory TF activity (*Zbtb7a*) in treated myeloid cells. (B) Violin plots of key ferroptosis and iron metabolism gene expression in myeloid subsets from control and treated groups. (C-D) Cell-cell interaction chord diagrams, revealing weakened classical monocyte interactions and enhanced macrophage/non-classical monocyte interactions in the treated group. (E-F) Ligand-receptor interaction heatmaps for myeloid subsets, showing upregulation of complement activation (C1qb-Cd14) and oxidative stress response (Tnf-Tnfrsf1a) pathways in the treated group. (G) Interaction counts network analysis, confirming rewired myeloid cell communication in the treated group.

Supplementary Figure 4 *K. pintolopesii* remodels splenic B cell intercellular communication and ligand-receptor signaling in aged mice. (A) Cell-cell interaction chord diagrams, showing enhanced B cell interactions with T cells/neutrophils and diminished interactions with erythrocytes/monocytes in the treated group. (B-C) Ligand-receptor interaction heatmaps for B cell subsets, showing upregulation of pro-inflammatory/senescence-associated L-R pairs (ProstaglandinE2-PTGER, CD48-CD244) and activation-related ligands (APP-CD74) in the treated group. (D) Quantitative interaction counts analysis, confirming increased total B cell interactions and strengthened MZBCells_Cr2-MemoryB_Ighg2b interactions in the treated group, with reduced naive-mature B cell interactions.

Supplementary Table 1 Splenic metabolomic profiling in aged mice: relative abundance of metabolites in control (C1–C10) and K. pintolopesii-treated (T1–T10) groups.

Supplementary Table 2 Differentially expressed genes (n = 900; FDR < 0.05) identified by bulk RNA-seq in the spleens of aged mice: KP + AGS versus KP comparison.

## Glossary

AGS: Astragaloside IV
ACSL4: acyl-CoA synthetase long-chain family member 4
BMDMs: bone marrow-derived macrophages
cDC1: conventional type 1 dendritic cells
cDC2: conventional type 2 dendritic cells
CFU: colony-forming units
CytoTRACE: CytoTRACE
D50/D60: diversity at 50%/60% coverage
DAB: 3,3’-diaminobenzidine
DAPI: 4’,6-diamidino-2-phenylindole
DEG: differentially expressed gene
FC: fold change
FBS: fetal bovine serum
Foxo: forkhead box O
FPKM: Fragments Per Kilobase of transcript per Million mapped reads
FTH1: ferritin heavy chain 1
GEMs: gel beads-in-emulsion
GO: Gene Ontology
GPX4: glutathione peroxidase 4
GRN: gene regulatory network
GSA: Genome Sequence Archive
GSEA: Gene Set Enrichment Analysis
H&E: Hematoxylin-eosin
HRP: horseradish peroxidase
IHC: Immunohistochemical
IL: interleukin
IMGT: International ImMunoGeneTics information system
Inverse Simpson: inverse Simpson diversity index
IOD: integrated optical density
KEGG: Kyoto Encyclopedia of Genes and Genomes
Leica RM2255: Leica microtome
LM22: leukocyte signature matrix 22
MACS: Magnetic-activated cell sorting
mDCs: mature dendritic cells
MOD: mean optical density
Monocle3: Monocle3 software
MZBCells: marginal zone B cells
NF-κB: nuclear factor kappa-light-chain-enhancer of activated B cells
NKT: natural killer T cells
Nrf2: nuclear factor erythroid 2-related factor 2
PCA: Principal Component Analysis
PBS: phosphate-buffered saline
PAMPs: pathogen-associated molecular patterns
RIN: RNA integrity number
ROS: reactive oxygen species
RPMI: Roswell Park Memorial Institute
RSS: region-specific score
SASP: senescence-associated secretory phenotype
scRNA-seq: single-cell RNA sequencing
snRNA-seq: single-nucleus RNA sequencing
TCR: T cell receptor
Tfh: follicular helper T cells
TF: transcription factor
TLR: Toll-like receptor
TNF: tumor necrosis factor
TRA: T cell receptor alpha chain
TRB: T cell receptor beta chain
TFR2: transferrin receptor 2
UMAP: Uniform Manifold Approximation and Projection
V-J: variable-joining.

## References

[1] Cavey T Latour C Island M Leroyer P Guggenbuhl P Coppin H . Spleen iron, molybdenum, and manganese concentrations are coregulated in hepcidin-deficient and secondary iron overload models in mice. FASEB J. (2019) 33:11072–81. doi: 10.1096/fj.201801381rr 31298936

[2] Comita S Falco P Mezzanotte M Vujic Spasic M Roetto A . Lack of Hfe and TfR2 in macrophages impairs iron metabolism in the spleen and the bone marrow. Int J Mol Sci. (2024) 25:9142. doi: 10.3390/ijms25179142 PMC11395440 39273097

[3] Shi Q Chen Y Li Y Qin S Yang Y Gao Y . Cross-tissue multicellular coordination and its rewiring in cancer. Nature. (2025) 643:529–38. doi: 10.1038/s41586-025-09053-4 40437094 PMC12240829

[4] Bronte V Pittet MJ . The spleen in local and systemic regulation of immunity. Immunity. (2013) 39:806–18. doi: 10.1016/j.immuni.2013.10.010 24238338 PMC3912742

[5] Mebius RE Kraal G . Structure and function of the spleen. Nat Rev Immunol. (2005) 5:606–16. doi: 10.1038/nri1669 16056254

[6] Yousefzadeh MJ Flores RR Zhu Y Schmiechen ZC Brooks RW Trussoni CE . An aged immune system drives senescence and ageing of solid organs. Nature. (2021) 594:100–5. doi: 10.1038/s41586-021-03547-7 33981041 PMC8684299

[7] Ezuz D Ombashe H Watad L Rakhymzhanova A Pandey S Atar O . Heme and iron toxicity in the aged spleen impairs T cell immunity through iron deprivation. Nat Aging. (2025) 5:2247–62. doi: 10.1038/s43587-025-00981-4 41107597 PMC12618244

[8] Gaffke L Szczudlo Z Podlacha M Cyske Z Rintz E Mantej J . Impaired ion homeostasis as a possible associate factor in mucopolysaccharidosis pathogenesis: transcriptomic, cellular and animal studies. Metab Brain Dis. (2022) 37:299–310. doi: 10.1007/s11011-021-00892-4 34928474 PMC8784502

[9] Ha J Doguer C Flores SRL Wang T Collins JF . Progressive increases in dietary iron are associated with the emergence of pathologic disturbances of copper homeostasis in growing rats. J Nutr. (2018) 148:373–8. doi: 10.1093/jn/nxx070 29546308 PMC6669950

[10] He Z He W Hu C Liao J Deng W Sun H . Cross-species comparison illuminates the importance of iron homeostasis for splenic anti-immunosenescence. Aging Cell. (2023) 22:e13982. doi: 10.1111/acel.13982 37681451 PMC10652311

[11] Zhu B Yang C Hua S Li K Shang P Chen X . Lithium enhances ferroptosis sensitivity in melanoma cells and promotes CD8(+) T cell infiltration and differentiation. Free Radical Bio Med. (2025) 227:233–45. doi: 10.1016/j.freeradbiomed.2024.12.012 39645207

[12] Khan NJ Choudhury S Ismatsara M Sultana N Sumi SA Jabeen L . Number and diameter of white pulp of spleen in different age and sex groups of Bangladeshi people. Mymensingh Med J. (2022) 31:406–11. 35383758

[13] Lee M Kim M Han KH Yoon CS . Age-related changes in liver, kidney, and spleen stiffness in healthy children measured with acoustic radiation force impulse imaging. Eur J Radiol. (2013) 82:e290–4. doi: 10.1016/j.ejrad.2013.01.018 23433651

[14] Chen L Xu S Wang S Chen H Han Y . Xiaohuafuning tang intervenes liver-depression-and-spleen-deficiency syndrome chronic-atrophic-gastritis by reshaping amino acid metabolism through gut microbiota. Phytomedicine. (2025) 136:156346. doi: 10.1016/j.phymed.2024.156346 39740378

[15] Zhang Z Chen W Chan H Peng J Zhu P Li J . Polystyrene microplastics induce size-dependent multi-organ damage in mice: Insights into gut microbiota and fecal metabolites. J Hazard Mater. (2024) 461:132503. doi: 10.1016/j.jhazmat.2023.132503 37717443

[16] Bayat M Hassannejad N Ghazanfari H . Evaluation of intrinsic and acquired immune cells infiltration in kidney and spleen of the mice infected with systemic candidiasis and treated with chloroform fraction of Zataria Multiflora Boiss. Microb Pathogenesis. (2018) 114:444–8. doi: 10.1016/j.micpath.2017.11.032 29183758

[17] Lin G Ou W Yang J Chen D Wang Y Wu A . Correlation of intestinal bacteria, fungi and dietary nutrient intake in NAFLD patients with spleen deficiency syndrome. Front Cell Infect Mi. (2025) 15:1586212. doi: 10.3389/fcimb.2025.1586212 40861494 PMC12375650

[18] Mansano ESB de Morais GR Moratto EM Sato F Baesso ML Medina AN . Combination of histopathology and FT-Raman spectroscopy for the study of experimental paracoccidioidomycosis in the spleen. Photochem Photobiol. (2018) 94:88–94. doi: 10.1111/php.12840 28881447

[19] Chen X Li P Liu M Zheng H He Y Chen M . Gut dysbiosis induces the development of pre-eclampsia through bacterial translocation. Gut. (2020) 69:513–22. doi: 10.1136/gutjnl-2019-319101 31900289

[20] Spadoni I Zagato E Bertocchi A Paolinelli R Hot E Di Sabatino A . A gut-vascular barrier controls the systemic dissemination of bacteria. Science. (2015) 350:830–4. doi: 10.1126/science.aad0135 26564856

[21] Liu J Lai X Yu R Ding H Bai H Yang Z . Progranulin aggravates lethal Candida albicans sepsis by regulating inflammatory response and antifungal immunity. PloS Pathog. (2022) 18:e1010873. doi: 10.1371/journal.ppat.1010873 36121866 PMC9521894

[22] Takagi J Singh-Babak SD Lohse MB Dalal CK Johnson AD . Candida albicans white and opaque cells exhibit distinct spectra of organ colonization in mouse models of infection. PloS One. (2019) 14:e218037. doi: 10.1371/journal.pone.0218037 31170229 PMC6553767

[23] Botschuijver S Roeselers G Levin E Jonkers DM Welting O Heinsbroek SEM . Intestinal fungal dysbiosis is associated with visceral hypersensitivity in patients with irritable bowel syndrome and rats. Gastroenterology. (2017) 153:1026–39. doi: 10.1053/j.gastro.2017.06.004 28624575

[24] Yu K Choi I Kim M Pyung YJ Lee J Choi Y . Florfenicol-induced dysbiosis impairs intestinal homeostasis and host immune system in laying hens. J Anim Sci Biotechnol. (2025) 16:56. doi: 10.1186/s40104-025-01186-w 40223090 PMC11995664

[25] Chen X Zhang J Wang Y Hu Q Zhao R Zhong L . Structure and immunostimulatory activity studies on two novel Flammulina velutipes polysaccharides: revealing potential impacts of -->6)-alpha-D-Glcp(1--> on the TLR-4/MyD88/NF-kappaB pathway. Food Funct. (2024) 15:3507–21. doi: 10.1039/d3fo05468c 38465397

[26] Fiers WD Leonardi I Iliev ID . From birth and throughout life: Fungal microbiota in nutrition and metabolic health. Annu Rev Nutr. (2020) 40:323–43. doi: 10.1146/annurev-nutr-013120-043659 32680437 PMC7529963

[27] Taubel M Jalanka J Kirjavainen PV Tuoresmaki P Hyvarinen A Skevaki C . Fungi in early-life house dust samples and the development of asthma: A birth cohort study. Ann Am Thorac Soc. (2023) 20:1456–64. doi: 10.1513/annalsats.202303-187oc PMC10559140 37535826

[28] Zhang H Wei Y Jia H Chen D Tang X Wang J . Immune activation of characteristic gut mycobiota Kazachstania pintolopesii on IL-23/IL-17R signaling in ankylosing spondylitis. Front Cell Infect Mi. (2022) 12:1035366. doi: 10.3389/fcimb.2022.1035366 36605130 PMC9808786

[29] Zhang H Li L Tan D Lin C Zhang Y Chen D . Kazachstania pintolopesii triggers an immune-endothelial-fibroblast cascade and drives inflammatory arthritis and tissue fibrosis in genetically susceptible hosts. Front Cell Infect Mi. (2025) 15:1738184. doi: 10.3389/fcimb.2025.1738184 41459160 PMC12738364

[30] Gao Y Wang T Nan N Tian F Tan L Yan H . Fungal commensal promotes intestinal repair via its secreted peptide in mice. Nat Microbiol. (2026) 11:476–91. doi: 10.1038/s41564-025-02233-y 41513998

[31] Han G Yunker R Hasan MH Bruce A Baldaro K Pacia J . A host-adapted commensal fungus from pet store mice drives type 2 immunity and cross-kingdom protection. In: bioRxiv [preprint]. Cold Spring Harbor, NY: Cold Spring Harbor Laboratory (2025). doi: 10.1101/2025.04.30.651489 PMC13486987 42621152

[32] James SA Parker A Purse C Telatin A Baker D Holmes S . The cynomolgus macaque intestinal mycobiome is dominated by the Kazachstania genus and K. pintolopesii species. J Fungi. (2022) 8:1054. doi: 10.3390/jof8101054 36294619 PMC9605169

[33] Liao Y Gao IH Kusakabe T Lin W Grier A Pan X . Fungal symbiont transmitted by free-living mice promotes type 2 immunity. Nature. (2024) 636:697–704. doi: 10.1038/s41586-024-08213-2 39604728 PMC11733984

[34] Liu L Zheng Z You W Yang P Wen Y Qiao Y . Vitamin C inhibits ACSL4 to alleviate ferro-aging in primates. Cell Metab. (2026) 38(4):673–93.e17. doi: 10.1016/j.cmet.2026.02.010 41819088

[35] Liang J Yang C Li P Zhang M Xie X Xie X . Astragaloside IV inhibits AOM/DSS-induced colitis-associated tumorigenesis via activation of PPARgamma signaling in mice. Phytomedicine. (2023) 121:155116. doi: 10.1016/j.phymed.2023.155116 37776619

[36] Wang J Wang L Feng X Xu Y Zhou L Wang C . Astragaloside IV attenuates fatty acid-induced renal tubular injury in diabetic kidney disease by inhibiting fatty acid transport protein-2. Phytomedicine. (2024) 134:155991. doi: 10.1016/j.phymed.2024.155991 39217653

[37] Wang J Pu X Zhuang H Guo Z Wang M Yang H . Astragaloside IV alleviates septic myocardial injury through DUSP1-Prohibitin 2 mediated mitochondrial quality control and ER-autophagy. J Adv Res. (2025) 75:561–80. doi: 10.1016/j.jare.2024.10.030 39550027 PMC12789773

[38] Wu S Zhou M Zhou H Han L Liu H . Astragaloside IV-loaded biomimetic nanoparticles target IkappaBalpha to regulate neutrophil extracellular trap formation for sepsis therapy. J Nanobiotechnol. (2025) 23:155. doi: 10.1186/s12951-025-03260-x 40022068 PMC11869569

[39] Xia D Li W Tang C Jiang J . Astragaloside IV, as a potential anticancer agent. Front Pharmacol. (2023) 14:1065505. doi: 10.3389/fphar.2023.1065505 36874003 PMC9981805

[40] Liang Y Chen B Liang D Quan X Gu R Meng Z . Pharmacological effects of Astragaloside IV: A review. Molecules. (2023) 28:6118. doi: 10.3390/molecules28166118 37630371 PMC10458270

[41] Chen S Zhou Y Chen Y Gu J . fastp: an ultra-fast all-in-one FASTQ preprocessor. Bioinformatics. (2018) 34:i884–90. doi: 10.1093/bioinformatics/bty560 30423086 PMC6129281

[42] Chen S . fastp 1.0: An ultra-fast all-round tool for FASTQ data quality control and preprocessing. Imeta. (2025) 4:e70078. doi: 10.1002/imt2.70078 PMC12527978 41112039

[43] Dobin A Davis CA Schlesinger F Drenkow J Zaleski C Jha S . STAR: ultrafast universal RNA-seq aligner. Bioinformatics. (2013) 29(1):15–21. doi: 10.1093/bioinformatics/bts635 PMC3530905 23104886

[44] Hao Y Hao S Andersen-Nissen E Mauck WM 3rd Zheng S Butler A . Integrated analysis of multimodal single-cell data. Cell. (2021) 184(13):3573–3587. doi: 10.1016/j.cell.2021.04.048 PMC8238499 34062119

[45] Cao J Spielmann M Qiu X Huang X Ibrahim DM Hill AJ . The single-cell transcriptional landscape of mammalian organogenesis. Nature. (2019) 566(7745):496–502. doi: 10.1038/s41586-019-0969-x PMC6434952 30787437

[46] Kim D Paggi JM Park C Bennett C Salzberg SL . Graph-based genome alignment and genotyping with HISAT2 and HISAT-genotype. Nat Biotechnol. (2019) 37(8):907–15. doi: 10.1038/s41587-019-0201-4 PMC7605509 31375807

[47] Tang H Finn RD Thomas PD . TreeGrafter: Phylogenetic tree-based annotation of proteins with Gene Ontology terms and other annotations. Bioinformatics. (2019) 35:518–20. doi: 10.1093/bioinformatics/bty625 30032202 PMC6361231

[48] Kanehisa M Goto S . KEGG: Kyoto encyclopedia of genes and genomes. Nucleic Acids Res. (2000) 28:27–30. doi: 10.1093/nar/28.1.27 10592173 PMC102409

[49] Fan Z Turiel G Ardicoglu R Ghobrial M Masschelein E Kocijan T . Exercise-induced angiogenesis is dependent on metabolically primed ATF3/4(+) endothelial cells. Cell Metab. (2021) 33:1793–807. doi: 10.1016/j.cmet.2021.07.015 34358431 PMC8432967

[50] Hu S Li R Gong D Hu P Xu J Ai Y . Atf3-mediated metabolic reprogramming in hepatic macrophage orchestrates metabolic dysfunction-associated steatohepatitis. Sci Adv. (2024) 10:eado3141. doi: 10.1126/sciadv.ado3141 39047111 PMC11268416

[51] Liu S Li Z Lan S Hao H Baz AA Yan X . The dual roles of activating transcription factor 3 (ATF3) in inflammation, apoptosis, ferroptosis, and pathogen infection responses. Int J Mol Sci. (2024) 25:824. doi: 10.3390/ijms25020824 38255898 PMC10815024

[52] Cai J Zhou X Zhuang Y Cui L Ma R Chen Y . Reprogramming of fatty acid metabolism via PPARalpha-orchestrated FADS2 in keratinocytes modulates skin inflammation in psoriasis. Adv Sci. (2025) 12:e17049. doi: 10.1002/advs.202417049 40878384 PMC12561275

[53] Lagrost L Masson D . The expanding role of lyso-phosphatidylcholine acyltransferase-3 (LPCAT3), a phospholipid remodeling enzyme, in health and disease. Curr Opin Lipidol. (2022) 33:193–8. doi: 10.1097/mol.0000000000000820 35165232

[54] Di Y Wang Z Xiao J Zhang X Ye L Wen X . ACSL6-activated IL-18R1-NF-kappaB promotes IL-18-mediated tumor immune evasion and tumor progression. Sci Adv. (2024) 10:eadp719. doi: 10.1126/sciadv.adp0719 PMC11409972 39292786

[55] Palacio L Goyer M Maggiorani D Espinosa A Villeneuve N Bourbonnais S . Restored immune cell functions upon clearance of senescence in the irradiated splenic environment. Aging Cell. (2019) 18:e12971. doi: 10.1111/acel.12971 31148373 PMC6612633

[56] Dixon SJ Olzmann JA . The cell biology of ferroptosis. Nat Rev Mol Cell Bio. (2024) 25:424–42. doi: 10.1038/s41580-024-00703-5 38366038 PMC12187608

[57] Li T Xie Y Shi L Sun Y Wen J Deng Z . TSC1 suppresses macrophage necroptosis for the control of infection by fungal pathogen Candida albicans. Immunohorizons. (2021) 5:90–101. doi: 10.4049/immunohorizons.2000093 33597177

[58] Yu M Song X Liu B Luan T Liao S Zhao Z . The emerging role of mast cells in response to fungal infection. Front Immunol. (2021) 12:688659. doi: 10.3389/fimmu.2021.688659 34149729 PMC8209461

[59] Shi J Xuan Y Zhang Q Chen J Zhu W Zhang H . Astragaloside IV attenuates chronic prostatitis by activating Keap1/Nrf2/HO-1 pathway: Suppressing ferroptosis and enhancing antioxidant defense. J Inflammation Res. (2025) 18:10061–79. doi: 10.2147/jir.s527722 40761383 PMC12318839

[60] Borowicz KK Jach ME . Astragalus membranaceus-can it delay cellular aging? Nutrients. (2025) 17:1299. doi: 10.3390/nu17081299 40284164 PMC12029721

[61] Xiao H Cao Y Wang Z Liu C . Recent advances in Astragaloside IV modulate immune response and skin cells promoting wound healing. Tissue Eng Part B Rev. (2026). doi: 10.1177/19373341251381830 41051944

[62] Zhang J Wu C Gao L Du G Qin X . Astragaloside IV derived from Astragalus membranaceus: A research review on the pharmacological effects. Adv Pharmacol. (2020) 87:89–112. doi: 10.1016/bs.apha.2019.08.002 32089240

[63] Chan KL Poller WC Swirski FK Russo SJ . Central regulation of stress-evoked peripheral immune responses. Nat Rev Neurosci. (2023) 24:591–604. doi: 10.1038/s41583-023-00729-2 37626176 PMC10848316

[64] Pasciuto E Burton OT Roca CP Lagou V Rajan WD Theys T . Microglia require CD4 T cells to complete the fetal-to-adult transition. Cell. (2020) 182:625–40. doi: 10.1016/j.cell.2020.06.026 32702313 PMC7427333

[65] Zhu X Huang J Dong W Tang H Xu S Wu Q . Somatosensory cortex and central amygdala regulate neuropathic pain-mediated peripheral immune response via vagal projections to the spleen. Nat Neurosci. (2024) 27:471–83. doi: 10.1038/s41593-023-01561-8 38291284

[66] Liu X Wang S Wu X Zhao Z Jian C Li M . Astragaloside IV alleviates depression in rats by modulating intestinal microbiota, T-immune balance, and metabolome. J Agr Food Chem. (2024) 72:259–73. doi: 10.1021/acs.jafc.3c04063 38064688

